# Improved Synthesis of 1-*O*-Acyl-β-d-Glucopyranose Tetraacetates

**DOI:** 10.3390/molecules22040662

**Published:** 2017-04-21

**Authors:** Yu Chen, Huan Lu, Yanyu Chen, Wansheng Yu, Hui Dai, Xianhua Pan

**Affiliations:** 1 School of Perfume and Aroma Technology, Shanghai Institute of Technology, 100 Haiquan Rd., Shanghai 201418, China; chenyu@sit.edu.cn (Y.C.); 156071203@mail.sit.edu.cn (Y.C.); 2 Shanghai Research Institute of Fragrance and Flavor Industry, 480 Nanning Rd., Shanghai 200232, China; aijiudu@sina.com (H.L.); yu102658@126.com (W.Y.); ddai_hui@126.com (H.D.)

**Keywords:** glucosyl esters, glucosyl bromide, aromatic acids, aliphatic acids

## Abstract

An improved synthesis of 1-*O*-acyl glucosyl esters that avoids the use of expensive Ag reagents as well as the hydrolysis of unstable glucosyl bromides is reported. Notably, β-configuration products were obtained exclusively in good yields.

## 1. Introduction

Numerous glycosyl esters have been investigated because of their biologically activity. Compounds such as tuliposide-A and tuliposide-B show bacteriotoxic and fungitoxic effects [1,2]. Some saturated fatty acid glycosyl esters were examined for antitumor activity [3]. In addition, glycosyl esters have also been used in cosmetics, detergents, oral-care products and medical supplies as flavor precursors.

**Scheme 1:**
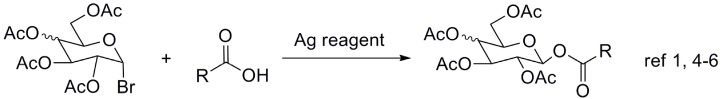
Glycosylation of carboxylic acids promoted by Ag catalysts.

The fact that few 1-*O*-acyl glycosyl esters have been found in Nature, has led to the development of various synthetic methods to access these compounds. The Koenigs-Knorr reaction using glycosyl bromide and an acid is the most attractive. Several publications have disclosed the glycosylation of carboxylic acids promoted by Ag catalysts through Koenigs-Knorr reaction (**1a**) [4,5,6]. However, the need for expensive Ag catalysts (at least one equivalent) has limited its application (Scheme 1).

**Scheme 2:**
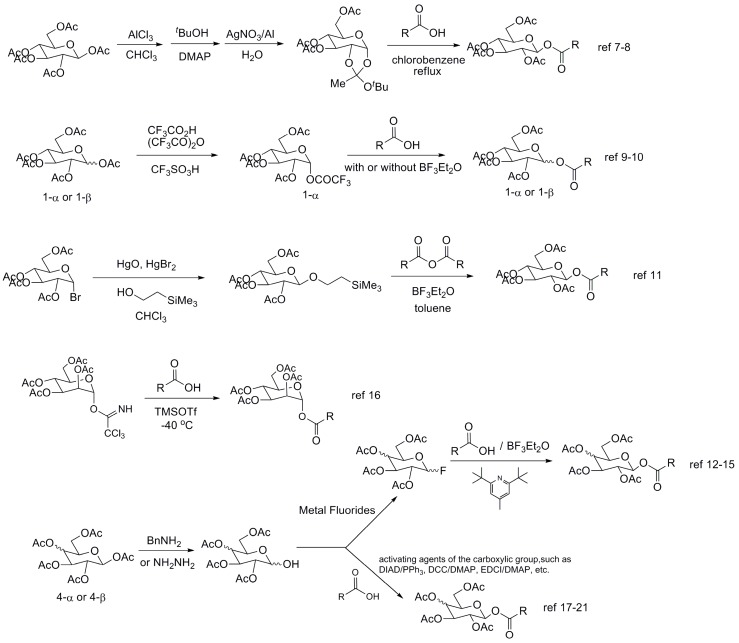
Other alternative methods.

Therefore, other alternative methods have been reported (Scheme 2), involving compounds such as orthoesters [7,8], trifluoroacetates [9,10], TMSET glycosides [11], glucosyl fluorides [12,13,14,15], trichloroacetimidates [16], etc. In addition, the activation of the carboxylic acid group using the Mitsunobu protocol [17], DCC [18,19,20] or EDCI [21] were also explored. However several drawbacks including troublesome preparation of the intermediates, the use of toxic reagents or the harsh conditions of these methods, make the reactions challenging.

## 2. Results and Discussion

Among the 1-*O*-acyl glycosyl esters, 1-*O*-acyl glucosyl esters are the most important and common. The formation of 1-*O*-acyl glucosyl esters by condensation of acids with glucosyl bromide in aqueous/DCM in the presence of an inorganic base seemed to be a good choice [22], but in our hands this reaction gave low yields for most substrates when run on a larger scale (1 g), with lactol **4** (as an α/β mixture) being formed during the condensation. The reason was found to be the hydrolysis of the glucosyl bromide **1** in the presence of H_2_O. Herein, we describe the improvement of this synthesis and preparation of a series of glucosyl esters.

We started to study this reaction with benzoic acid (**2**) which was reacted with α-glucosyl bromide **1** in the presence of tricaprylylmethylammonium chloride (a mixture of C_8_-C_10_ species in which C_8_ is dominant, sold under the brand name Aliquat 336^®^) as the phase transfer catalyst (PTC). From Table 1, we can see that the reaction was greatly influenced by water. The more water added, the more compound **4** was formed in the reaction (Table 1, entries 1–3). When only DCM was used as the solvent, product **3** was obtained in high yield, with less than 5% of the lactol **4** (Table 1, entry 4). Considering 0.5 equiv. of water would be formed in the reaction with K_2_CO_3_ itself, 4 Å molecular sieves (4 Å MS) were added, which increased the yield by 6% (Table 1, entry 5). It was found that K_2_CO_3_ was the best base after comparing different ones according to the yield and the cost (Table 1, entries 6–10). The reaction was completely suppressed when NaOH or Et_3_N were used as the base with recycled compound **1**, probably because of the instability of the PTC in the presence of stronger base (Table 1, entry 9) or due to the weaker basicity of Et_3_N (Table 1, entry 10). Notably, compared and in contrast with the known data [9,10,11,23,24] the β-configuration product was exclusively obtained through SN_2_ substitution,.

Next, the PTC and the solvent were varied. From Table 2, it seems that the reaction did not happen without a PTC. Only 10% mol of a PTC such as tetrabutylammonium bromide (TBAB), tetraethylammonium bromide (TEAB), benzyltriethylammonium chloride (BTEAC), hexadecyl- trimethylammonium bromide (CTMAB) led the reaction to give the product in high yield (Table 2, entries 1–4).

In the comparison of the solvents, DCM proved to be the best solvent (Table 2, entries 6–8). The role of the PTC is unclear, but it seems to increase the solubility of carboxylate formed at the beginning of the reaction, due to the quite low solubility of the latter.

Next, various acids were chosen to verify the scope of this reaction (Table 3 and Table 4). Aromatic acids with different kind of substituent groups at different positions on benzene ring, gave the desired product in 80–99% yield. For example, electron-donating groups, such as methoxy, benzyloxy or methyl could all make the reaction happen smoothly (Table 3, entries 1–5). Electron-withdrawing group also produced the corresponding products in 85–99% yield (Table 3, entries 6–8). Similarly, β-naphtoic acid gave product **22** quantitatively (Table 3, entry 9). In the comparison experiments, the yield decreased evidently because compound **1** is sensitive to hydrolysis as described before when the reaction was conducted in the presence of water (Table 3, entry 1, 3, 7 and 9). The β-configuration of the products was confirmed by 2D-NMR data of compound **8** [25]

Not only aromatic acids, but aliphatic acids could be used in the reaction too. The results are listed in Table 4. Phenylacetic acid (**23**) and 2,4,5-trifluorophenylacetic acid (**25**) provided the corresponding product in no less than 95% yield (Table 4, entries 1–2). Good results were also obtained using other aliphatic acids. For example, isobutyric acid (**27**) and isovaleric acid (**29**) gave the products in more than 90% yield respectively. Lower yield was obtained for pivalic acid (**31**), probably due to the steric hindrance (Table 4, entries 3–5).

In addition, a long chain glucosyl ester was prepared in good yield from acid **33** (Table 4, entry 6). Satisfactorily, this reaction could be also be extended to aliphatic acids with olefins and rings (Table 4, entries 7–11). For the same reason as before, the results were not good when water was added in the comparison sample due to the hydrolysis of **1** (Table 4, entry 1, 4, 6 and 10).

It is noteworthy that when we tried to prepare two 1-*O*-acyl-β-d-glucopyranose tetraacetates on a large scale (**3** and **24**, more than 100 g), these could be purified without column chromatography. It seems that this method could be applicable in industrial manufacture due to the high yields generally obtained. The scaled-up synthesis of other compounds and the study of other kinds of glycosylation are now underway.

## 3. Materials and Methods

### 3.1. General Methods

All solvents and reagents, except for compound **1**, were purchased from the commercial supplier Tansoole (Shanghai, China) and were used without further purification. Compound **1** was prepared according to the known method [26]. 4 Å MS were activated at 600 °C for one-day and kept in a dessicator. The progress of the reactions was assessed by thin-layer chromatography (TLC) with GF_254_ silica-gel precoated sheets using EtOAc/hexane as eluent. Column chromatography was performed on silica gel (200–300 mesh) using EtOAc/hexane or EtOAc/petroleum ether as eluent. ^1^H (400 MHz) and ^13^C (100 MHz) NMR spectra were recorded on an Avance 400 spectrometer (Bruker, Karlsruhe, Germany) in CDCl_3_ using tetramethylsilane (TMS) as internal standards. 2D-NMR was recorded on a Bruker Avance 500 spectrometer. *J* values were given in Hertz. Mass spectra a high resolution mass spectra were recorded on an ESQUIRE-LC mass spectrometer (Agilent, Palo Alto, CA, USA). Elemental analysis was performed on an Elemental Vario-III CHN analyzer (Elementar Analysensysteme GmbH, Hanau, Germany). Optical rotations were measured on a WZZ-2S polarimeter (Suoguang Electric Tech Co., Shanghai, China) in DCM, with concentrations denoted in g/100 mL. Melting points were determined on a SGW-X4 melting point instruments (Shenguang Instrument Co., Ltd., Shanghai, China).

### 3.2. General Procedure for the Synthesis of 1-O-Acyl-β-d-Glucopyranose Tetraacetates

A mixture of glucosyl bromide **1** (1.03 g, 2.5 mmol), acid (5.0 mmol), K_2_CO_3_ (0.69 g, 5.0 mmol), TEAB (0.05 g, 0.25 mmol) and 4 Å MS (0.25 g) in 35 mL DCM was stirred 24–48 h at room temperature. Next, the insoluble substances, made up of the slightly soluble potassium carboxylate, 4 Å MS and other salts, were filtered off. The filtrate was washed with water, and the separated organic layer was then washed with 25% aqueous K_2_CO_3_ to removed any remaining potassium carboxylate. After drying over MgSO_4_ and concentration in vacuo, the residue was purified via silica gel column chromatography using EtOAc/hexane or EtOAc/petroleum ether (1:10 to 1:1) as eluents to yield the desired product.

### 3.3. Scaled-Up Synthesis of Compound ***3***

A mixture of glucosyl bromide **1** (150.0 g, 0.36 mol), benzoic acid **2** (89.0 g, 0.73 mol), K_2_CO_3_ (100.7 g, 0.73 mol), TEAB (7.5 g, 36 mmol) and 4 Å MS (36.0 g) in 5 L DCM was stirred 24 h at room temperature. Next, the insoluble substances, made up of the slightly soluble potassium benzoate, 4 Å MS and other salts, were filtered off. The filtrate was washed by water, and the separated organic layer was then washed with 25% aqueous K_2_CO_3_ to remove any remaining potassium benzoate. After drying over MgSO_4_ and concentration in vacuo, the crude was purified in refluxing EtOH to give **3** as a white solid in 89% yield after cooling down.

### 3.4. Scaled-Up Synthesis of Compound ***24***

A mixture of glucosyl bromide **1** (150.0 g, 0.36 mol), phenylacetic acid **23** (99.3 g, 0.73 mol), K_2_CO_3_ (100.7 g, 0.73 mol), TEAB (7.5 g, 36 mmol) and 4 Å MS (36.0 g) in 5 L DCM was stirred 26 h at room temperature. After work-up as described in Section 3.3, compound **24** was obtained as a white solid in 78% yield after cooling down.

*1-O-Benzoyl-2,3,4,6-tetra-O-acetyl-β-d-glucopyranose* (**3**). White solid, m.p. 143–144 °C; [α${]}_{\text{D}}^{20}$ = +55.6 (*c* = 0.5, DCM); ^1^H-NMR: δ = 1.99 (s, 3H), 2.04 (s, 3H), 2.05 (s, 3H), 2.07 (s, 3H), 3.93–3.97 (m, 1H), 4.14 (dd, *J* = 2.0, 12.8 Hz, 1H), 4.33 (dd, *J* = 4.4, 12.4 Hz, 1H), 5.18–5.23 (m, 1H), 5.34–5.37 (m, 2H), 5.93–5.95 (m, 1H) [9,11,27], 7.46 (t, *J* = 8.0 Hz, 1H), 7.61 (t, *J* = 7.2 Hz, 1H), 8.05 (dd, *J* = 1.2, 8.0 Hz, 2H); ^13^C-NMR: δ = 20.45, 20.49, 20.51, 20.58, 61.4, 67.9, 70.1, 72.6, 72.7, 92.2 [23,28], 128.4, 128.6, 130.1, 133.9, 164.4, 169.3, 169.4, 170.0, 170.5; ESI-MS (*m*/*z*) 475 [M + Na]^+^; HRMS calcd. for C_21_H_24_O_11_ 452.1330, found 452.1321; Anal. Calcd. for C_21_H_24_O_11_ (%): C, 55.75; H, 5.35. Found: C, 55.85; H, 5.26.

*1-O-(2-Methoxybenzoyl)-2,3,4,6-tetra-O-acetyl-β-d-glucopyranose* (**6**). White solid, m.p. 89–90 °C; [α${]}_{\text{D}}^{20}$ = +71.2 (*c* = 0.5, DCM); ^1^H-NMR: δ = 2.01 (s, 3H), 2.04 (s, 3H), 2.05 (s, 3H), 2.07 (s, 3H), 3.91 (s, 3H), 3.91–3.94 (m, 1H), 4.14 (dd, *J* = 2.0, 12.8 Hz, 1H), 4.33 (dd, *J* = 4.4, 12.4 Hz, 1H), 5.17–5.22 (m, 1H), 5.31–5.33 (m, 2H), 5.95–5.97 (m, 1H), 6.97–7.01 (m, 2H), 7.50–7.55 (m, 1H), 7.87 (dd, *J* = 1.6, 8.0 Hz, 2H); ^13^C-NMR: δ = 20.44, 20.46, 20.5, 20.6, 55.7, 61.5, 67.8, 70.2, 72.6, 72.8, 91.8, 112.0, 117.4, 120.1, 132.4, 134.8, 160.1, 163.2, 169.2, 169.3, 170.0, 170.4; ESI-MS (*m*/*z*) 505 [M + Na]^+^; Anal. Calcd. for C_22_H_26_O_12_ (%): C, 54.77; H, 5.43. Found: C, 54.90; H, 5.30.

*1-O-(3,4-Dimethoxybenzoyl)-2,3,4,6-tetra-O-acetyl-β-d-glucopyranose* (**8**). White solid, m.p. 135–136 °C; [α${]}_{\text{D}}^{20}$ = +75.6 (*c* = 0.5, DCM); ^1^H-NMR: δ = 1.99 (s, 3H), 2.04 (s, 3H), 2.06 (s, 3H), 2.08 (s, 3H), 3.94 (s, 3H), 3.95 (s, 3H), 3.96–3.98 (m, 1H, H-5), 4.14 (dd, *J* = 2.0, 12.4 Hz, 1H, H-6), 4.33 (dd, *J* = 4.4, 12.8 Hz, 1H, H-6), 5.18–5.23 (m, 1H, H-4), 5.34–5.36 (m, 2H, H-3 and H-2), 5.88–5.90 (m, 1H, H-1), 6.91 (d, *J* = 8.4 Hz, 1H), 7.54 (d, *J* = 2.0 Hz, 1H), 7.70 (dd, *J* = 1.6, 8.0 Hz, 2H); ^13^C-NMR: δ = 20.5, 20.6, 55.9, 56.0, 61.4 (C-6), 67.9 (C-4), 70.1 (C-2), 72.5 (C-3), 72.6 (C-5), 92.2 (C-1), 110.4, 112.2, 120.6, 124.5, 148.7, 153.8, 164.1, 169.3, 169.4, 170.0, 170.5; ESI-MS (*m*/*z*) 535 [M + Na]^+^; Anal. Calcd. for C_23_H_28_O_13_ (%): C, 53.91; H, 5.51. Found: C, 54.00; H, 5.65.

*1-O-(4-Benzyloxy-3-methoxybenzoyl)-2,3,4,6-tetra-O-acetyl-β-d-glucopyranose* (**10**). White solid, m.p. 126–127 °C; [α${]}_{\text{D}}^{20}$ = +27.5 (*c* = 0.5, DCM); ^1^H-NMR: δ = 1.98 (s, 3H), 2.04 (s, 3H), 2.05 (s, 3H), 2.07 (s, 3H), 3.92–3.96 (m, 1H), 3.94 (s, 3H), 4.14 (dd, *J* = 2.4, 12.4 Hz, 1H), 4.33 (dd, *J* = 4.4, 12.4 Hz, 1H), 5.17–5.21 (m, 1H), 5.2 (s, 3H), 5.33–5.35 (m, 2H), 5.87–5.89 (m, 1H), 6.91 (d, *J* = 8.8 Hz, 1H), 7.32–7.44 (m, 5H), 7.55 (d, *J* = 1.6 Hz, 1H), 7.62 (dd, *J* = 1.6, 8.4 Hz, 1H); ^13^C-NMR: δ = 20.6, 20.7, 56.1, 61.5, 67.9, 70.2, 70.7, 72.5, 72.7, 92.2, 112.5, 112.7, 120.9, 124.3, 127.2, 128.1, 128.7, 136.1, 149.2, 152.9, 164.2, 169.3, 169.4, 170.0, 170.6; ESI-MS (*m*/*z*) 611 [M + Na]^+^; Anal. Calcd. for C_29_H_32_O_13_ (%): C, 59.18; H, 5.48. Found: C, 59.01; H, 5.60.

*1-O-(3,4,5-Trimethoxybenzoyl)-2,3,4,6-tetra-O-acetyl-β-d-glucopyranose* (**12**). White solid, m.p. 55–56 °C; [α${]}_{\text{D}}^{20}$ = +26.9 (*c* = 0.5, DCM); ^1^H-NMR: δ = 1.97 (s, 3H), 2.02 (s, 3H), 2.03 (s, 3H), 2.05 (s, 3H), 3.85–3.95 (m, 1H), 3.88 (s, 9H), 4.12 (d, *J* = 12.8 Hz, 1H), 4.32 (dd, *J* = 4.4, 12.8 Hz, 1H), 5.16–5.20 (m, 1H), 5.30–5.36 (m, 2H), 5.83–5.85 (m, 1H), 7.28 (s, 2H); ^13^C-NMR: δ = 20.4, 20.5, 20.6, 56.2, 60.8, 61.4, 67.9, 70.2, 72.3, 72.6, 92.4, 107.3, 123.1, 142.9, 152.9, 164.0, 169.3, 169.4, 170.0, 170.5; ESI-MS (*m*/*z*) 565 [M + Na]^+^; Anal. Calcd. for C_24_H_30_O_14_ (%): C, 53.14; H, 5.57. Found: C, 53.31; H, 5.71.

*1-O-(2,5-Dimethylbenzoyl)-2,3,4,6-tetra-O-acetyl-β-d-glucopyranose* (**14**). Syrup; [α${]}_{\text{D}}^{20}$ = +78.3 (*c* = 0.5, DCM); ^1^H-NMR: δ = 1.94 (s, 3H), 1.97 (s, 3H), 1.98 (s, 3H), 2.00 (s, 3H), 2.28 (s, 3H), 2.48 (s, 3H), 3.84–3.88 (m, 1H), 4.07 (dd, *J* = 2.4, 12.4 Hz, 1H), 4.26 (dd, *J* = 4.4, 12.4 Hz, 1H), 5.11–5.16 (m, 1H), 5.25–5.28 (m, 2H), 5.86–5.88 (m, 1H), 7.07 (d, *J* = 8.0 Hz, 1H), 7.18 (d, *J* = 8.0 Hz, 1H), 7.69 (s, 1H); ^13^C-NMR: δ = 20.53, 20.56, 20.6, 20.7, 21.4, 21.5, 61.5, 67.9, 70.3, 72.7, 72.9, 91.9, 127.0, 131.7, 131.8, 133.9, 135.6, 138.4, 165.0, 169.3, 169.5, 170.2, 170.7; ESI-MS (*m*/*z*) 503 [M + Na]^+^; Anal. Calcd. for C_23_H_28_O_11_ (%): C, 57.50; H, 5.87. Found: C, 57.60; H, 5.79.

*1-O-(3-Bromobenzoyl)-2,3,4,6-tetra-O-acetyl-β-d-glucopyranose* (**16**). White solid, m.p. 119–120 °C; [α${]}_{\text{D}}^{20}$ = +50.7 (*c* = 0.5, DCM); ^1^H-NMR: δ = 2.00 (s, 3H), 2.05 (s, 3H), 2.06 (s, 3H), 2.08 (s, 3H), 3.92–3.97 (m, 1H), 4.14 (dd, *J* = 2.0, 12.8 Hz, 1H), 4.33 (dd, *J* = 4.4, 12.4 Hz, 1H), 5.17–5.22 (m, 1H), 5.33–5.35 (m, 2H), 5.92–5.94 (m, 1H), 7.35 (t, *J* = 8.0 Hz, 1H), 7.72–7.75 (m, 1H), 7.95–7.98 (m, 1H), 8.18 (t, *J* = 1.6 Hz, 1H); ^13^C-NMR: δ = 20.50, 20.53, 20.55, 20.6, 61.4, 67.8, 70.1, 72.5, 72.7, 92.5, 122.6, 128.6, 130.2, 130.4, 133.0, 136.9, 163.2, 169.3, 169.4, 170.0, 170.5; ESI-MS (*m*/*z*) 553 [M + Na]^+^; Anal. Calcd. for C_21_H_23_BrO_11_ (%): C, 47.47; H, 4.36. Found: C, 47.50; H, 4.41.

*1-O-(2-Chloro-4-fluorobenzoyl)-2,3,4,6-tetra-O-acetyl-β-d-glucopyranose* (**18**). White solid, m.p. 116–117 °C; [α${]}_{\text{D}}^{20}$ = +72.2 (*c* = 0.5, DCM); ^1^H-NMR: δ = 2.00 (s, 3H), 2.03 (s, 3H), 2.04 (s, 3H), 2.07 (s, 3H), 3.90–3.94 (m, 1H), 4.13 (dd, *J* = 2.0, 12.0 Hz, 1H), 4.32 (dd, *J* = 4.8, 12.4 Hz, 1H), 5.15–5.20 (m, 1H), 5.30–5.33 (m, 2H), 5.92–5.93 (m, 1H), 7.03–7.08 (m, 1H), 7.21 (dd, *J* = 6.8, 8.4 Hz, 1H), 7.97 (dd, *J* = 2.0, 6.0 Hz, 1H); ^13^C-NMR: δ = 20.5, 20.6, 61.4, 67.7, 70.1, 72.6, 72.8, 92.3, 114.4 (d, *J* = 21.7 Hz), 119.0 (d, *J* = 24.5 Hz), 123.6 (d, *J* = 3.4 Hz), 134.5 (d, *J* = 9.9 Hz), 137.1 (d, *J* = 10.7 Hz), 161.8, 164.7 (d, *J* = 257.1 Hz), 169.2, 169.3, 170.0, 170.5; ESI-MS (*m*/*z*) 527 [M + Na]^+^; HRMS calcd for C_21_H_22_ClFO_11_ 504.0808, found 504.0805; Anal. Calcd. for C_21_H_22_ClFO_11_ (%): C, 49.96; H, 4.39. Found: C, 49.62; H, 4.46.

*1-O-(3-Nitrobenzoyl)-2,3,4,6-tetra-O-acetyl-β-d-glucopyranose* (**20**). White solid, m.p. 109–110 °C; [α${]}_{\text{D}}^{20}$ = +32.9 (*c* = 0.5, DCM); ^1^H-NMR: δ = 1.99 (s, 3H), 2.03 (s, 3H), 2.04 (s, 3H), 2.07 (s, 3H), 3.93–3.97 (m, 1H), 4.13 (dd, *J* = 2.0, 12.0 Hz, 1H), 4.31 (dd, *J* = 4.4, 12.8 Hz, 1H), 5.17–5.21 (m, 1H), 5.33–5.35 (m, 2H), 5.94–5.96 (m, 1H), 7.68 (t, *J* = 8.0 Hz, 1H), 8.32–8.35 (m, 1H), 8.44–8.46 (m, 1H), 8.86–8.87 (m, 1H); ^13^C-NMR: δ = 20.4, 20.5, 20.6, 61.4, 67.8, 70.1, 72.3, 72.8, 92.8, 125.1, 128.2, 130.0, 130.2, 135.5, 148.3, 162.5, 169.2, 169.4, 170.0, 170.5; ESI-MS (*m*/*z*) 520 [M + Na]^+^; Anal. Calcd. for C_21_H_23_NO_13_ (%): C, 50.71; H, 4.66; N, 2.82. Found: C, 50.65; H, 4.78; N, 2.70.

*1-O-(2-Naphthoyl)-2,3,4,6-tetra-O-acetyl-β-d-glucopyranose* (**22**). White solid, m.p. 135–136 °C; [α${]}_{\text{D}}^{20}$ = +47.8 (*c* = 0.5, DCM); ^1^H-NMR: δ = 1.99 (s, 3H), 2.01 (s, 3H), 2.07 (s, 3H), 2.08 (s, 3H), 3.97–4.00 (m, 1H), 4.16 (dd, *J* = 2.0, 12.8 Hz, 1H), 4.35 (dd, *J* = 4.4, 12.4 Hz, 1H), 5.24 (t, *J* = 9.6 Hz, 1H), 5.35–5.44 (m, 2H), 6.01 (d, *J* = 8.0 Hz, 1H), 7.55–7.59 (m, 1H), 7.60–7.64 (m, 1H), 7.88–7.91 (m, 2H), 7.98 (d, *J* = 7.2 Hz, 1H), 8.04 (dd, *J* = 2.0, 8.8 Hz, 1H), 8.63 (d, *J* = 0.8 Hz, 1H); ^13^C-NMR: δ = 20.51, 20.56, 20.58, 20.6, 61.5, 67.9, 70.3, 72.7, 72.8, 92.4, 125.1, 125.6, 126.9, 127.8, 128.5, 128.8, 129.6, 132.2, 132.3, 135.9, 164.7, 169.4, 169.5, 170.1, 170.6; ESI-MS (*m*/*z*) 525 [M + Na]^+^; Anal. Calcd. for C_25_H_26_O_11_ (%): C, 59.76; H, 5.22. Found: C, 59.89; H, 5.15.

*1-O-(2-Phenylacetyl)-2,3,4,6-tetra-O-acetyl-β-d-glucopyranose* (**24**). White solid, m.p. 108–109 °C; [α${]}_{\text{D}}^{20}$ = +91.7 (*c* = 0.5, DCM); ^1^H-NMR: δ = 1.76 (s, 3H), 1.99 (s, 3H), 2.03 (s, 3H), 2.09 (s, 3H), 3.66 (s, 2H), 3.82–3.86 (m, 1H), 4.12 (dd, *J* = 2.0, 12.8 Hz, 1H), 4.30 (dd, *J* = 4.4, 12.4 Hz, 1H), 5.10–5.15 (m, 2H), 5.21 (t, *J* = 8.8 Hz, 1H), 5.69 (d, *J* = 7.6 Hz, 1H), 7.25–7.34 (m, 5H); ^13^C-NMR: δ = 20.2, 20.5, 20.6, 41.1, 61.4, 67.7, 69.9, 72.6, 72.7, 91.8, 127.4, 128.7, 129.2, 132.9, 169.0, 169.3, 169.4, 170.0, 170.5; ESI-MS (*m*/*z*) 489 [M + Na]^+^; HRMS calcd for C_22_H_26_O_11_ 466.1481, found 466.1477; Anal. Calcd. for C_22_H_26_O_11_ (%): C, 56.65; H, 5.62. Found: C, 56.78; H, 5.50; Anal. Calcd. for C_22_H_26_O_11_ (%): C, 56.65; H, 5.62. Found: C, 56.59; H, 5.68.

*1-O-(2-(2,4,5-Trifluorophenyl)acetyl)-2,3,4,6-tetra-O-acetyl-β-d-glucopyranose* (**26**). White solid, m.p. 100–101 °C; [α${]}_{\text{D}}^{20}$ = +49.4 (*c* = 0.5, DCM); ^1^H-NMR: δ = 1.99 (s, 3H), 2.01 (s, 3H), 2.03 (s, 3H), 2.09 (s, 3H), 3.67 (s, 2H), 3.82–3.87 (m, 1H), 4.12 (dd, *J* = 2.0, 12.8 Hz, 1H), 4.30 (dd, *J* = 4.4, 12.4 Hz, 1H), 5.10–5.15 (m, 2H), 5.25 (t, *J* = 8.8 Hz, 1H), 5.73 (d, *J* = 8.8 Hz, 1H), 6.91–6.98 (m, 1H), 7.07–7.13 (m, 1H); ^13^C-NMR: δ = 20.2, 20.4, 20.6, 33.5 (d, *J* = 1.9 Hz), 61.3, 67.6, 70.0, 72.5, 72.7, 92.2, 105.5 (dd, *J* = 20.5, 27.5 Hz), 116.5 (d, *J* = 17.5 Hz), 119.0 (dd, *J* = 5.6, 19.0 Hz), 146.6 (dd, *J* = 12.7, 243.1 Hz), 149.5 (d, *J* = 251.5 Hz), 156.0 (dd, *J* = 10.4, 243.5 Hz), 167.9, 169.0, 169.3, 170.0, 170.5; ESI-MS (*m*/*z*) 543 [M + Na]^+^; Anal. Calcd. for C_22_H_23_F_3_O_11_ (%): C, 50.77; H, 4.45. Found: C, 50.66; H, 4.50.

*1-O-Isobutyryl-2,3,4,6-tetra-O-acetyl-β-d-glucopyranose* (**28**). White solid, m.p. 108–109 °C; [α${]}_{\text{D}}^{20}$ = +49.2 (*c* = 0.5, DCM); ^1^H-NMR: δ = 1.16 (d, *J* = 7.2 Hz, 3H), 1.17 (d, *J* = 7.2 Hz, 3H), 2.02 (s, 6H), 2.04 (s, 3H), 2.09 (s, 3H), 2.57–2.64 (m, 1H), 3.83–3.87 (m, 1H), 4.12 (dd, *J* = 2.0, 12.4 Hz, 1H), 4.30 (dd, *J* = 4.4, 12.8 Hz, 1H), 5.12–5.19 (m, 2H), 5.26 (t, *J* = 8.8 Hz, 1H), 5.72 (d, *J* = 8.4 Hz, 1H); ^13^C-NMR: δ = 18.1, 18.7, 20.3, 20.4, 20.6, 33.7, 61.4, 67.8, 70.1, 72.6, 91.5, 169.0, 169.3, 169.9, 170.4, 174.9; ESI-MS (*m*/*z*) 441 [M + Na]^+^; Anal. Calcd. for C_18_H_26_O_11_ (%): C, 51.67; H, 6.26. Found: C, 51.79; H, 6.20.

*1-O-(3-Methylbutanoyl)-2,3,4,6-tetra-O-acetyl-β-d-glucopyranose* (**30**). White solid, m.p. 73–74 °C; [α${]}_{\text{D}}^{20}$ = +126 (*c* = 0.5, DCM); ^1^H-NMR: δ = 0.95 (d, *J* = 6.8 Hz, 6H), 2.02 (s, 6H), 2.04 (s, 3H), 2.08 (s, 3H), 2.10–2.12 (m, 1H), 2.25 (d, *J* = 6.4 Hz, 2H), 3.83–3.87 (m, 1H), 4.11 (dd, *J* = 2.0, 12.4 Hz, 1H), 4.30 (dd, *J* = 4.4, 12.8 Hz, 1H), 5.11–5.17 (m, 2H), 5.26 (t, *J* = 9.2 Hz, 1H), 5.74 (d, *J* = 8.4 Hz, 1H); ^13^C-NMR: δ = 20.3, 20.4, 20.5, 22.0, 25.4, 42.9, 61.4, 67.7, 70.1, 72.5, 72.7, 91.3, 168.9, 169.2, 169.9, 170.3, 170.8; ESI-MS (*m*/*z*) 455 [M + Na]^+^; Anal. Calcd. for C_19_H_28_O_11_ (%): C, 52.77; H, 6.53. Found: C, 52.90; H, 6.44.

*1-O-Pivaloyl-2,3,4,6-tetra-O-acetyl-β-d-glucopyranose* (**32**). White solid, m.p. 131–132 °C; [α${]}_{\text{D}}^{20}$ = +187 (*c* = 0.5, DCM); ^1^H-NMR: δ = 1.19 (s, 9H), 2.00 (s, 6H), 2.02 (s, 3H), 2.08 (s, 3H), 3.81–3.85 (m, 1H), 4.10 (dd, *J* = 2.4, 12.8 Hz, 1H), 4.29 (dd, *J* = 4.4, 12.0 Hz, 1H), 5.11–5.19 (m, 2H), 5.25 (t, *J* = 9.2 Hz, 1H), 5.66 (d, *J* = 8.8 Hz, 1H); ^13^C-NMR: δ = 20.3, 20.47, 20.48, 20.6, 26.6, 38.7, 61.4, 67.9, 70.0, 72.5, 72.6, 91.7, 169.0, 169.3, 170.0, 170.5, 176.4; ESI-MS (*m*/*z*) 455 [M + Na]^+^; Anal. Calcd. for C_19_H_28_O_11_ (%): C, 52.77; H, 6.53. Found: C, 52.89; H, 6.45.

*1-O-Dodecanoyl-2,3,4,6-tetra-O-acetyl-β-d-glucopyranose* (**34**). White solid, m.p. 53–54 °C; [α${]}_{\text{D}}^{20}$ = +102 (*c* = 0.5, DCM); ^1^H-NMR: δ = 0.88 (t, *J* = 9.2 Hz, 3H), 1.25–1.30 (m, 16H), 1.57–1.62 (m, 2H), 2.01 (s, 3H), 2.02 (s, 3H), 2.03 (s, 3H), 2.09 (s, 3H), 2.34–2.38 (m, 2H), 3.82–3.87 (m, 1H), 4.11 (dd, *J* = 2.0, 12.0 Hz, 1H), 4.30 (dd, *J* = 4.4, 12.0 Hz, 1H), 5.11–5.16 (m, 2H), 5.26 (t, *J* = 9.2 Hz, 1H), 5.73 (d, *J* = 8.4 Hz, 1H); ^13^C-NMR: δ = 14.0, 20.42, 20.46, 20.58, 20.59, 22.6, 24.5, 28.8, 29.1, 29.2, 29.3, 29.5, 31.8, 33.9, 61.4, 67.8, 70.2, 72.6, 72.7, 91.5, 169.1, 169.4, 170.0, 170.5, 171.7; ESI-MS (*m*/*z*) 553 [M + Na]^+^; Anal. Calcd. for C_26_H_42_O_11_ (%): C, 58.85; H, 7.98. Found: C, 58.98; H, 7.88.

*1-O-((E)-2-Methylpent-2-enoyl)-2,3,4,6-tetra-O-acetyl-β-d-glucopyranose* (**36**). Syrup; [α${]}_{\text{D}}^{20}$ = +39.5 (*c* = 0.5, DCM); ^1^H-NMR: δ = 1.05 (t, *J* = 8.0 Hz, 3H), 1.82 (s, 3H), 2.01 (s, 3H), 2.03 (s, 3H), 2.04 (s, 3H), 2.09 (s, 3H), 2.17–2.24 (m, 2H), 3.86–3.91 (m, 1H), 4.12 (dd, *J* = 2.0, 12.4 Hz, 1H), 4.31 (dd, *J* = 4.4, 12.4 Hz, 1H), 5.15 (t, *J* = 9.2 Hz, 1H), 5.21–5.32 (m, 2H), 5.75 (d, *J* = 8.0 Hz, 1H), 6.85 (dt, *J* = 1.2, 7.6 Hz, 1H); ^13^C-NMR: δ = 11.9, 12.7, 20.43, 20.47, 20.48, 20.6, 22.1, 61.4, 67.9, 70.1, 72.5, 72.6, 91.9, 125.7, 147.2, 165.7, 169.1, 169.3, 170.0, 170.5; ESI-MS (*m*/*z*) 467 [M + Na]^+^, HRMS calcd for C_20_H_28_O_11_ 444.1614, found 444.1618; Anal. Calcd. for C_20_H_28_O_11_ (%): C, 54.05; H, 6.35. Found: C, 53.95; H, 6.40.

*1-O-((E)-Oct-2-enoyl)-2,3,4,6-tetra-O-acetyl-β-d-glucopyranose* (**38**). Syrup; [α${]}_{\text{D}}^{20}$ = +124 (*c* = 0.5, DCM); ^1^H-NMR: δ = 0.86 (t, *J* = 7.2 Hz, 3H), 1.22–1.28 (m, 4H), 1.39–1.47 (m, 2H), 1.98 (s, 3H), 1.99 (s, 3H), 2.00 (s, 3H), 2.05 (s, 3H), 2.16–2.21 (m, 2H), 3.83–3.87 (m, 1H), 4.08 (dd, *J* = 2.0, 12.4 Hz, 1H), 4.27 (dd, *J* = 4.4, 12.4 Hz, 1H), 5.10–5.18 (m, 2H), 5.25 (t, *J* = 9.2 Hz, 1H), 5.75 (d, *J* = 7.6 Hz, 1H), 5.76–5.80 (m, 1H), 7.01–7.09 (m, 1H); ^13^C-NMR: δ = 13.8, 20.44, 20.48, 20.5, 20.6, 22.3, 27.4, 31.2, 32.3, 61.4, 67.8, 70.2, 72.6, 72.7, 91.6, 119.5, 153.1, 164.2, 169.2, 169.4, 170.0, 170.6; ESI-MS (*m*/*z*) 495 [M + Na]^+^; Anal. Calcd. for C_22_H_32_O_11_ (%): C, 55.92; H, 6.83. Found: C, 55.85; H, 6.90.

*1-O-((2E,6Z)-Nona-2,6-dienoyl)-2,3,4,6-tetra-O-acetyl-β-d-glucopyranose* (**40**). Syrup; [α${]}_{\text{D}}^{20}$ = +86.3 (*c* = 0.5, DCM); ^1^H-NMR: δ = 0.96 (t, *J* = 8.0 Hz, 3H), 2.01 (s, 3H), 2.02 (s, 3H), 2.02–2.05 (m, 2H), 2.04 (s, 3H), 2.08 (s, 3H), 2.19–2.23 (m, 2H), 2.25–2.29 (m, 2H), 3.85–3.89 (m, 1H), 4.11 (dd, *J* = 2.0, 12.4 Hz, 1H), 4.30 (dd, *J* = 4.4, 12.4 Hz, 1H), 5.12–5.21 (m, 2H), 5.27 (t, *J* = 9.2 Hz, 1H), 5.27–5.32 (m, 1H), 5.78 (d, *J* = 8.0 Hz, 1H), 5.80–5.86 (m, 1H), 7.04–7.11 (m, 1H); ^13^C-NMR: δ = 14.1, 20.4, 20.5, 20.6, 25.3, 32.4, 61.4, 67.8,70.2, 72.5, 72.7, 91.6, 120.0, 126.8, 133.0, 152.1, 164.0, 169.2, 169.3, 170.0, 170.5; ESI-MS (*m*/*z*) 507 [M + Na]^+^; Anal. Calcd. for C_23_H_32_O_11_ (%): C, 57.02; H, 6.66. Found: C, 57.12; H, 6.63.

*1-O-(Cyclopropanecarbonyl)-2,3,4,6-tetra-O-acetyl-β-d-glucopyranose* (**42**). White solid, m.p. 121–122 °C; [α${]}_{\text{D}}^{20}$ = +170.8 (*c* = 0.5, DCM); ^1^H-NMR: δ = 0.94–0.97 (m, 2H), 1.03–1.10 (m, 2H), 1.63–1.67 (m, 1H), 2.02 (s, 3H), 2.03 (s, 3H), 2.04 (s, 3H), 2.09 (s, 3H), 3.82–3.86 (m, 1H), 4.11 (dd, *J* = 2.0, 12.0 Hz, 1H), 4.30 (dd, *J* = 4.4, 12.4 Hz, 1H), 5.11–5.17 (m, 2H), 5.26 (t, *J* = 9.6 Hz, 1H), 5.72 (d, *J* = 8.0 Hz, 1H); ^13^C-NMR: δ = 9.3, 12.7, 20.4, 20.6, 61.4, 67.7, 70.2, 72.5, 72.6, 91.5, 169.1, 169.3, 169.9, 170.4, 172.8; ESI-MS (*m*/*z*) 439 [M + Na]^+^; Anal. Calcd. for C_18_H_24_O_11_ (%): C, 51.92; H, 5.81. Found: C, 51.99; H, 5.75.

*1-O-(Cyclohexanecarbonyl)-2,3,4,6-tetra-O-acetyl-β-d-glucopyranose* (**44**). White solid, m.p. 111–112 °C; [α${]}_{\text{D}}^{20}$ = +96 (*c* = 0.5, DCM); ^1^H-NMR: δ = 1.20–1.49 (m, 6H), 1.62–1.65 (m, 1H), 1.68–1.77 (m, 1H), 1.85–1.90 (m, 2H), 2.02 (s, 6H), 2.04 (s, 3H), 2.09 (s, 3H), 2.33–2.39 (m, 1H), 3.83–3.87 (m, 1H), 4.11 (dd, *J* = 2.0, 12.0 Hz, 1H), 4.30 (dd, *J* = 4.4, 12.4 Hz, 1H), 5.11–5.18 (m, 2H), 5.26 (t, *J* = 9.6 Hz, 1H), 5.72 (d, *J* = 8.4 Hz, 1H); ^13^C-NMR: δ = 20.3, 20.4, 20.6, 24.9, 25.3, 25.5, 28.1, 28.7, 42.5, 61.4, 67.8, 70.1, 72.5, 72.6, 91.4, 169.1, 169.3, 170.0, 170.5, 173.8; ESI-MS (*m*/*z*) 481 [M + Na]^+^; Anal. Calcd. for C_21_H_30_O_11_ (%): C, 55.02; H, 6.60. Found: C, 55.16; H, 6.52.

## 4. Conclusions

The formation of 1-*O*-acyl glucosyl esters by condensation of acids with glucosyl bromide was developed on a large scale in DCM without water. A diverse array of 1-*O*-acyl glucosyl esters were prepared in good yields, which seems to indicate that our reaction conditions could be applied to a broad substrate scope. In addition, scaled-up preparations were also successfully attempted.

## Figures and Tables

**Table 1:**
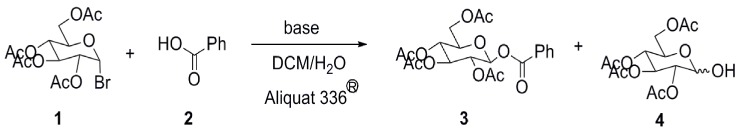
The influence of water and the screening of base for the reaction of **1** with **2** ^a^.

Entry	Base	H_2_O	3 ^b^	4 ^b^
1	K_2_CO_3_	5 mL (278 mmol) ^c^	35%	55%
2	K_2_CO_3_	2.5 mL (139 mmol)	54%	35%
3	K_2_CO_3_	0.5 mL (27.8 mmol)	78%	12%
4	K_2_CO_3_	-	88%	4%
5	K_2_CO_3_	- ^d^	94%	trace
6	Na_2_CO_3_	- ^d^	80%	trace
7	NaHCO_3_	- ^d^	69%	trace
8	Cs_2_CO_3_	- ^d^	90%	trace
9	NaOH	- ^d^	NR	trace
10	Et_3_N	- ^d^	NR	trace

^a^ The reaction was conducted with **1** (2.5 mmol), **2** (5 mmol), base (5 mmol) and Aliquat 336^®^ (0.25 mmol) in 35 mL DCM with or without H_2_O. ^b^ Isolated yield. ^c^ About 115 equiv. of H_2_O to glucosyl bromide was used according to reference 22. ^d^ 0.25 g 4 Å molecular sieve was added.

**Table 2:**
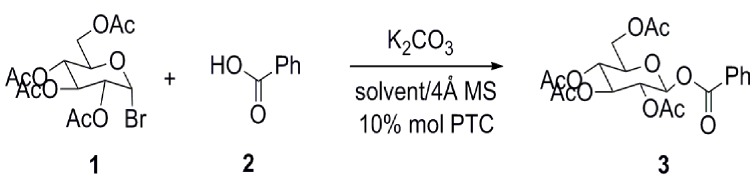
Variation of PTC and the solvent ^a^.

Entry	PTC	Solvent	3 ^b^
1	TBAB	DCM	99%
2	TEAB	DCM	99%
3	BEAC	DCM	96%
4	CMAB	DCM	97%
5	-	DCM	NR
6	TEAB	THF	<10%
7	TEAB	CH_3_CN	78%
8	TEAB	DMF	<10%

^a^ The reaction was conducted with **1** (2.5 mmol), **2** (5 mmol), K_2_CO_3_ (5 mmol), PTC (0.25 mmol) and 0.25 g 4 Å MS in 35 mL solvent. ^b^ Isolated yield.

**Table 3:** The reaction of glucosyl bromide **1** with aromatic acids ^a^.

Entry	Aromatic Acids	Product	Yield ^b^ (%)
1	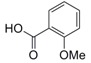 **5**	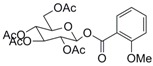 **6**	95 ^c^
2	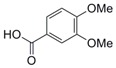 **7**	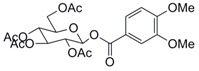 **8**	98
3	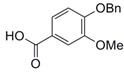 **9**	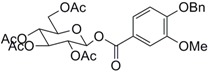 **10**	91 ^c^
4	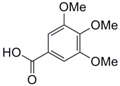 **11**	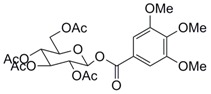 **12**	80
5	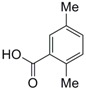 **13**	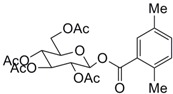 **14**	96
6	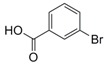 **15**	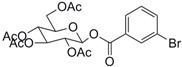 **16**	99
7	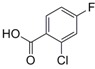 **17**	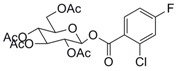 **18**	85 ^c^
8	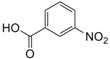 **19**	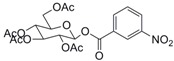 **20**	90
9	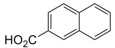 **21**	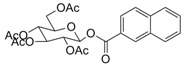 **22**	99 ^c^

^a^ The reaction was conducted with **1** (2.5 mmol), aromatic acid (5 mmol), K_2_CO_3_ (5 mmol), TEAB (0.25 mmol) and 0.25 g 4 Å MS in 35 mL DCM. ^b^ Isolated yield. ^c^ 28–54% yields of these compounds were obtained when 5 mL H_2_O was added in the reaction mixture.

**Table 4:** The reaction of glucosyl bromide **1** with aliphatic acids ^a^.

Entry	Aliphatic Acids	Product	Yield ^b^ (%)
1	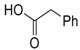 **23**	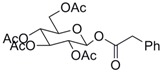 **24**	95 ^c^
2	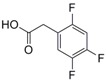 **25**	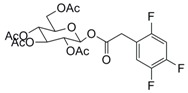 **26**	97
3	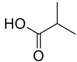 **27**	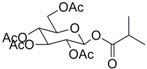 **28**	91
4	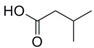 **29**	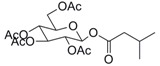 **30**	99 ^c^
5	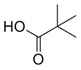 **31**	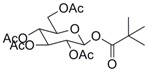 **32**	72
6	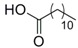 **33**	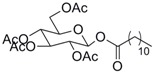 **34**	92 ^c^
7	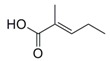 **35**	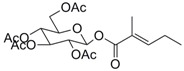 **36**	96
8	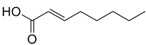 **37**	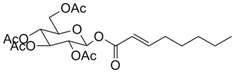 **38**	79
9	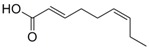 **39**	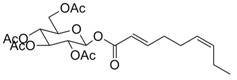 **40**	94
10	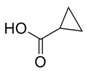 **41**	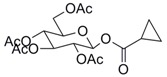 **42**	99 ^c^
11	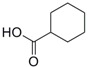 **43**	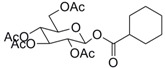 **44**	78

^a^ The reaction was conducted with **1** (2.5 mmol), aliphatic acid (5 mmol), K_2_CO_3_ (5 mmol), TEAB (0.25 mmol) and 0.25 g 4 Å MS in 35 mL DCM. ^b^ Isolated yield. ^c^ 40–58% yields of these compounds were obtained when 5 mL H_2_O was added to the reaction mixture.

## Supplementary Materials

Supplementary materials can be accessed online.
