# Supplementary material for: Improved Synthesis of 1-O-Acyl-β-d-Glucopyranose Tetraacetates
Source: Molecules. 2017 Apr 21;22(4):662. doi: 10.3390/molecules22040662 (PMC6154585; doi:10.3390/molecules22040662)
Supplement: Supplementary file 1 [file molecules-22-00662-s001.pdf]

Improved synthesis of 1-*O*-Acyl-β-D-glucopyranose Tetraacetates

Yu Chen,[1,2] *Huan Lu,*[2] *Yanyu Chen,*[1] *Wansheng Yu,*[2] *Hui Dai,*[2] *Xianhua Pan*[1,2,*]

1) School of Perfume and Aroma Technology, Shanghai Institute of Technology, 100 Haiquan Rd., Shanghai, 201418, P.R. China.

2) Shanghai Research Institute of Fragrance and Flavor Industry, 480 Nanning Rd., Shanghai, 200232, P.R. China.

Copies of ^{1}H NMR and ^{13}C NMR data

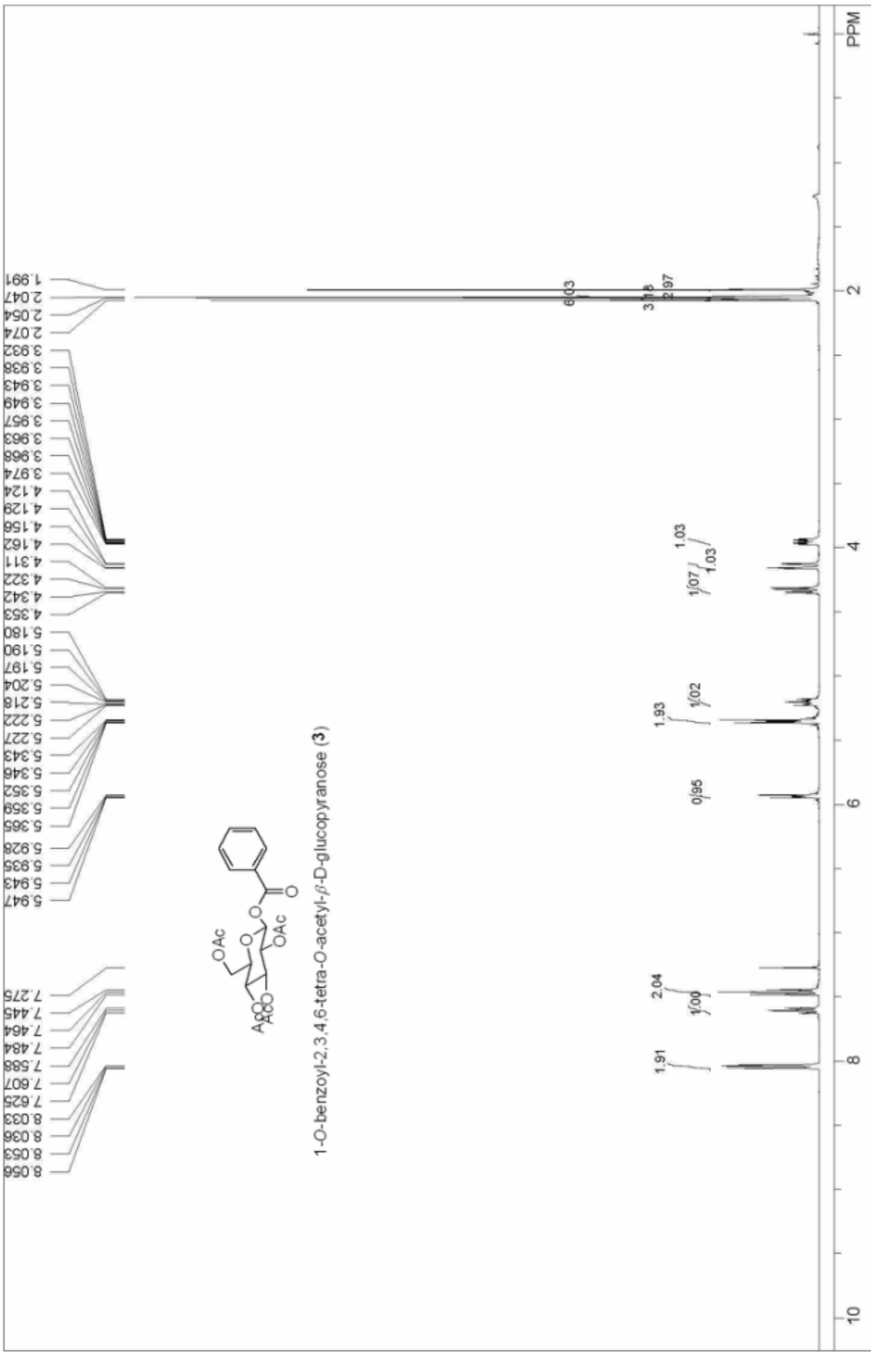

Figure S1. ^{1}H NMR spectrum of compound **3**

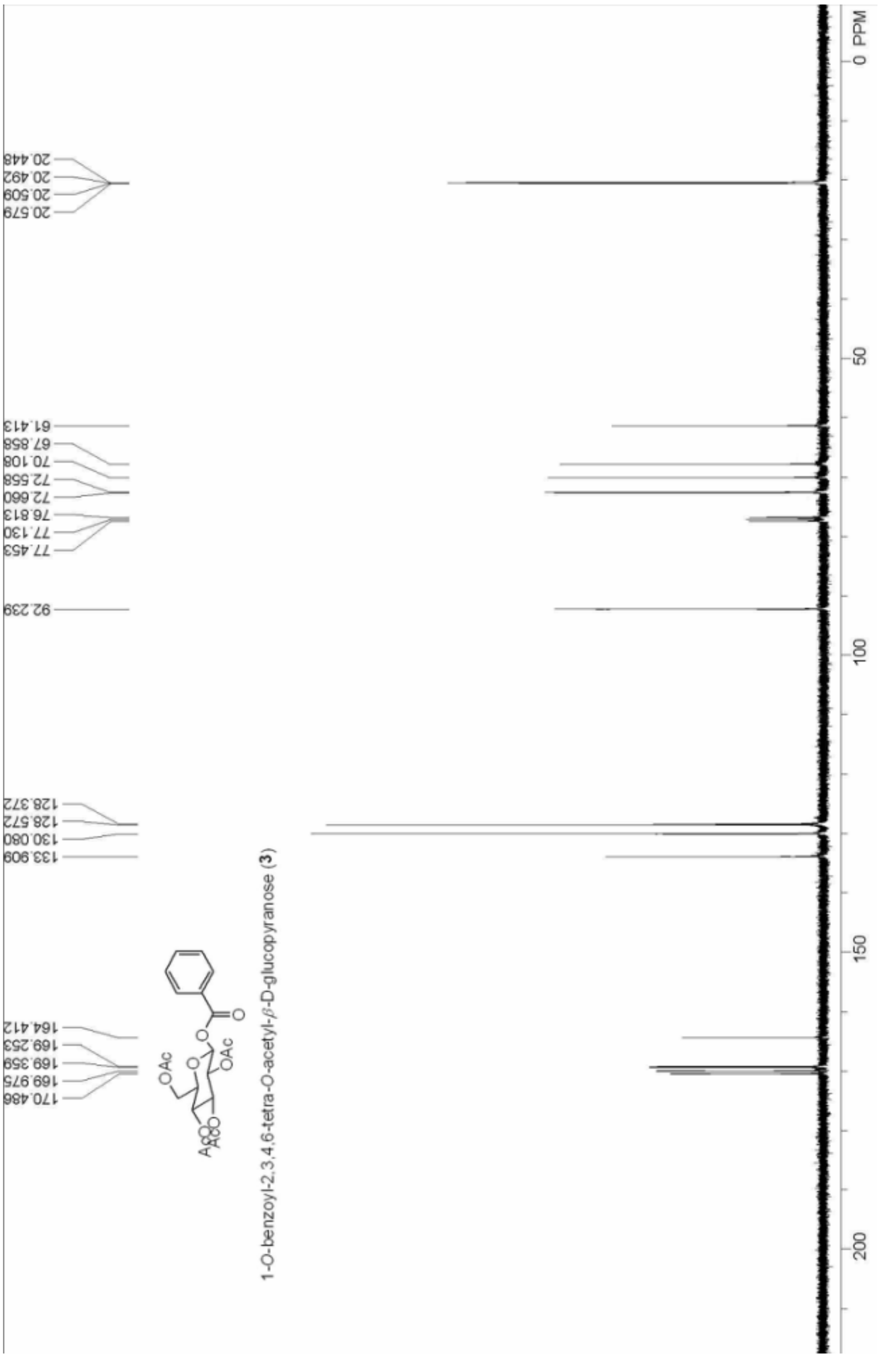

Figure S2. ^{13}C NMR spectrum of compound **3**

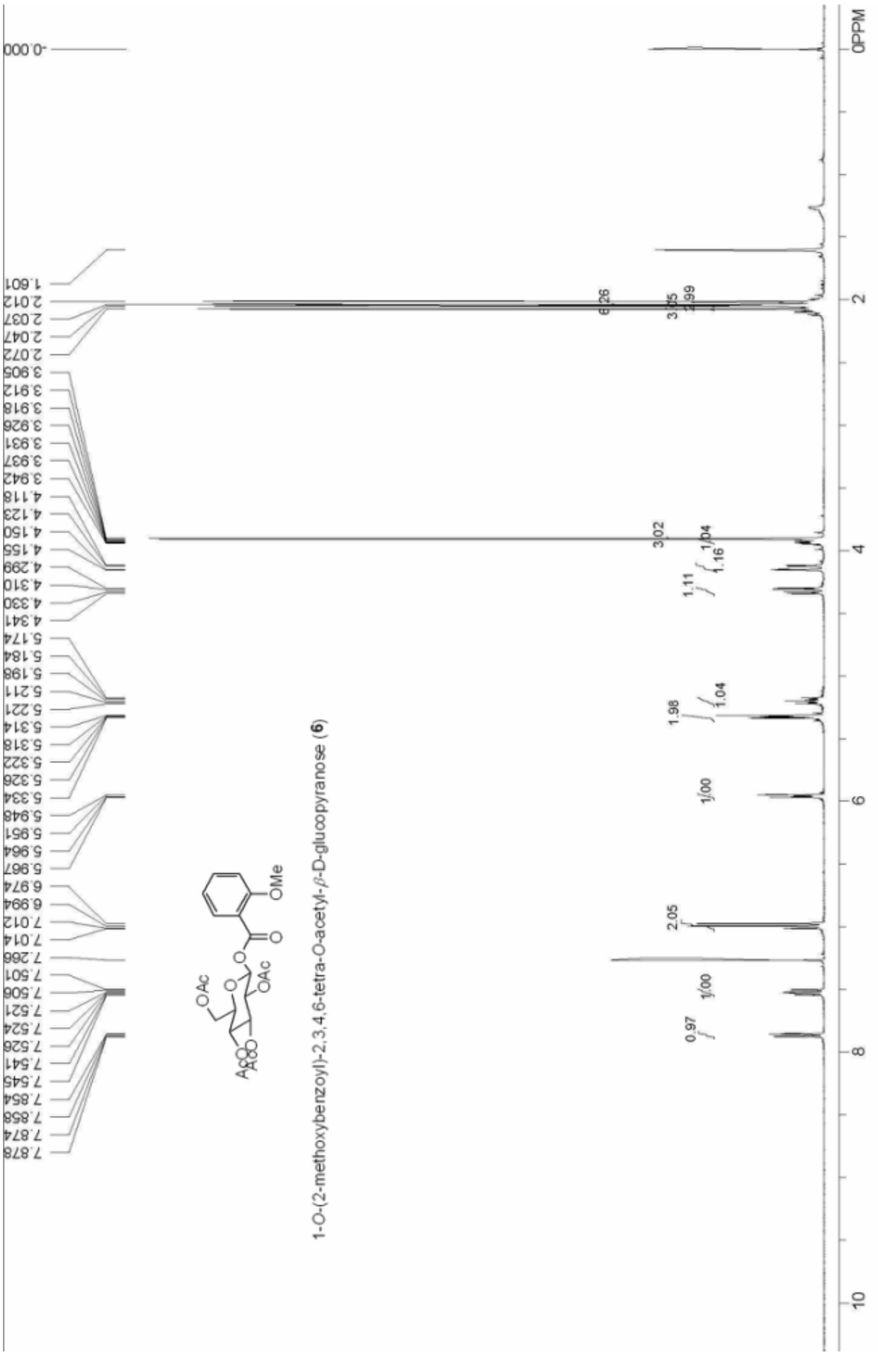

Figure S3. ^{1}H NMR spectrum of compound **6**

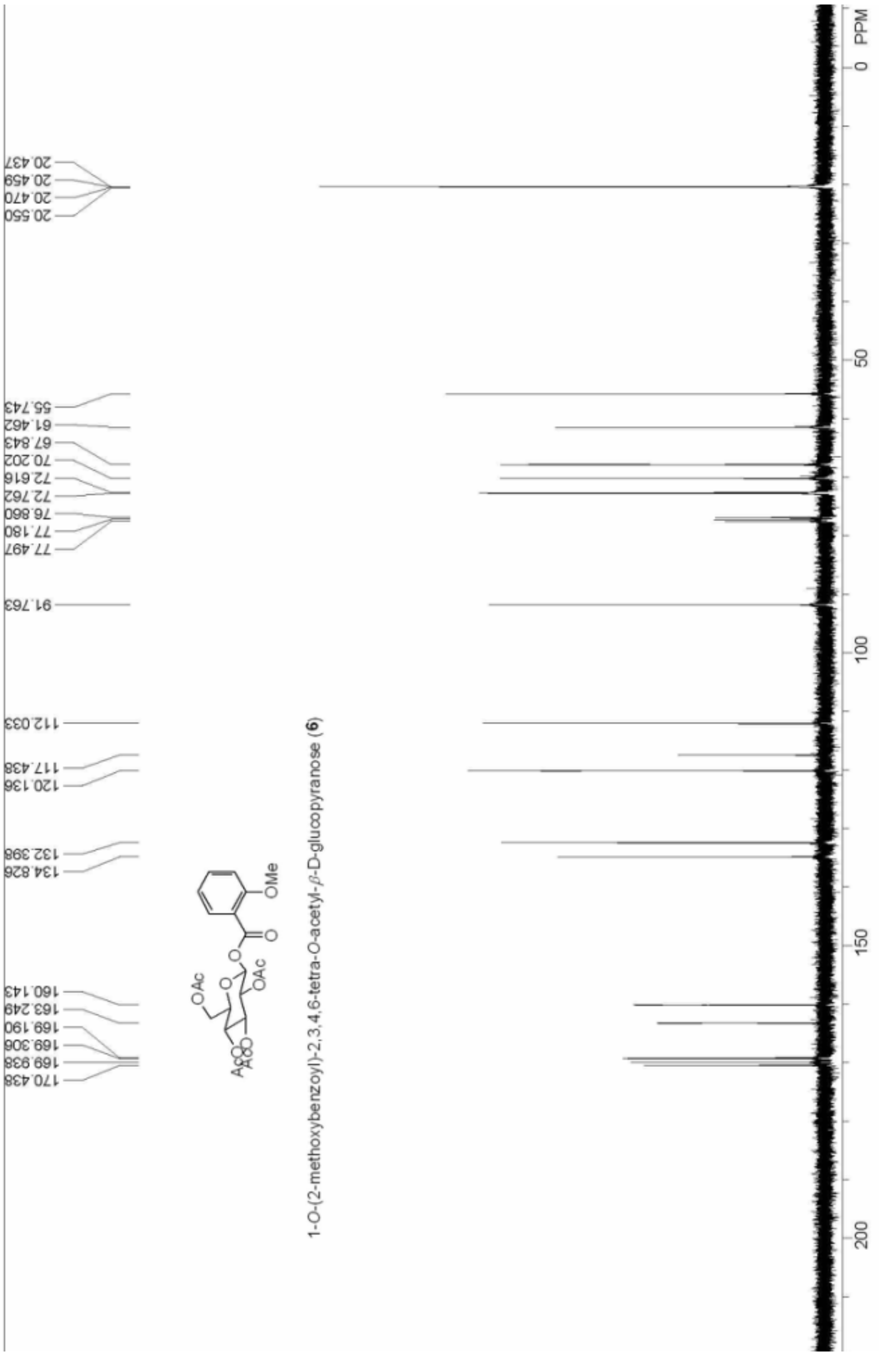

Figure S4. ^{13}C NMR spectrum of compound **6**

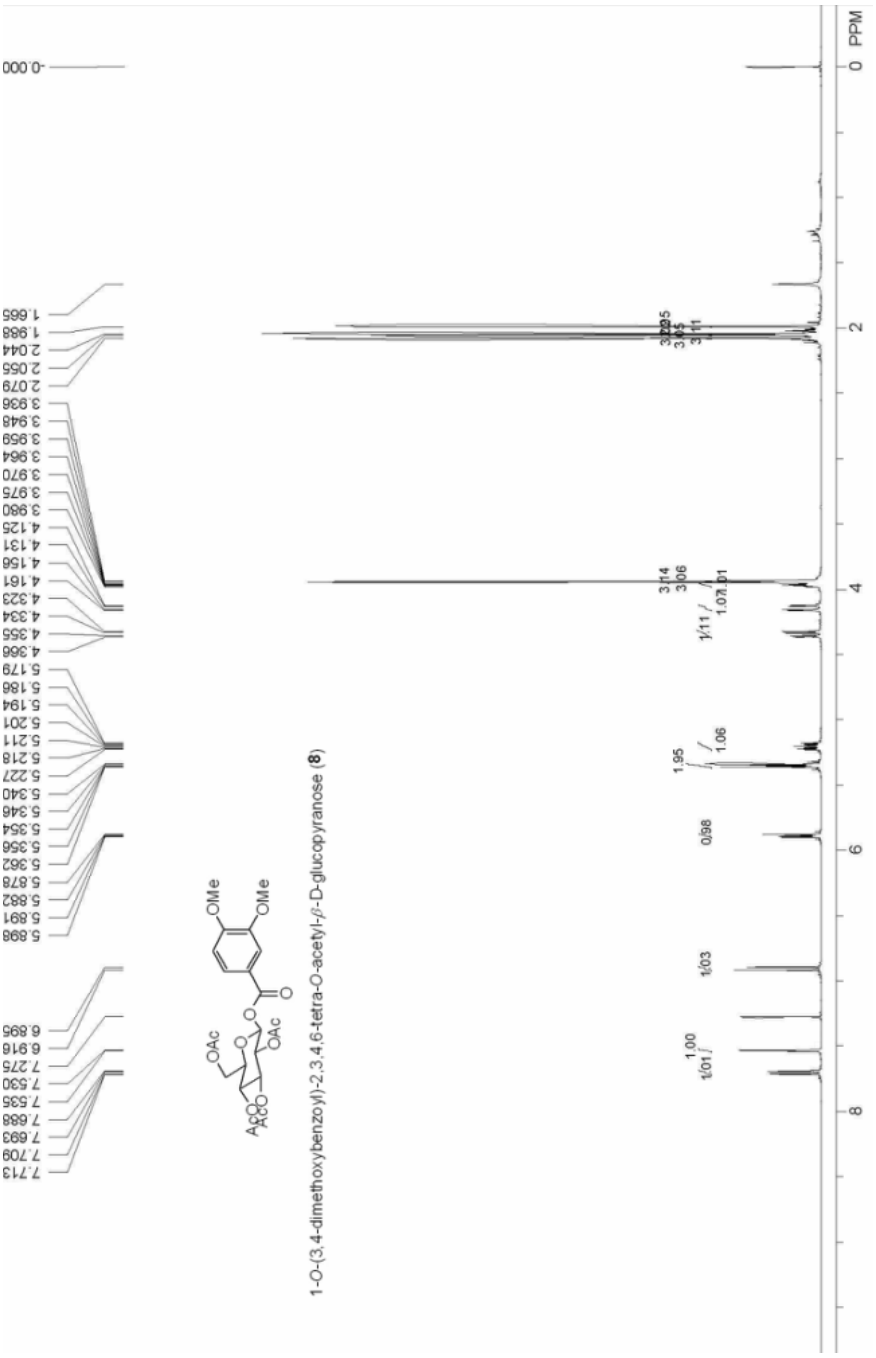

Figure S5. ^{1}H NMR spectrum of compound **8**

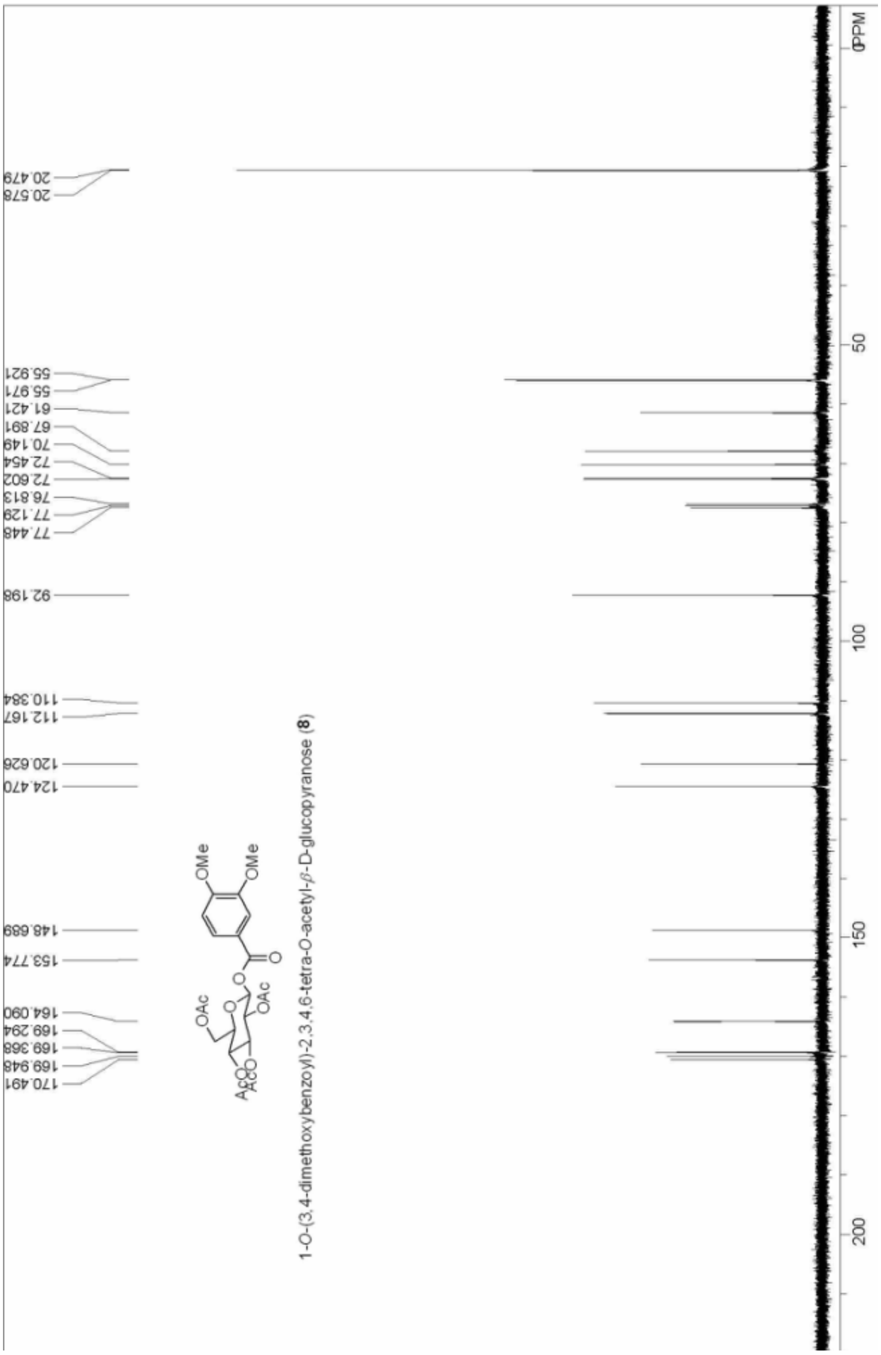

Figure S6. ^{13}C NMR spectrum of compound **8**

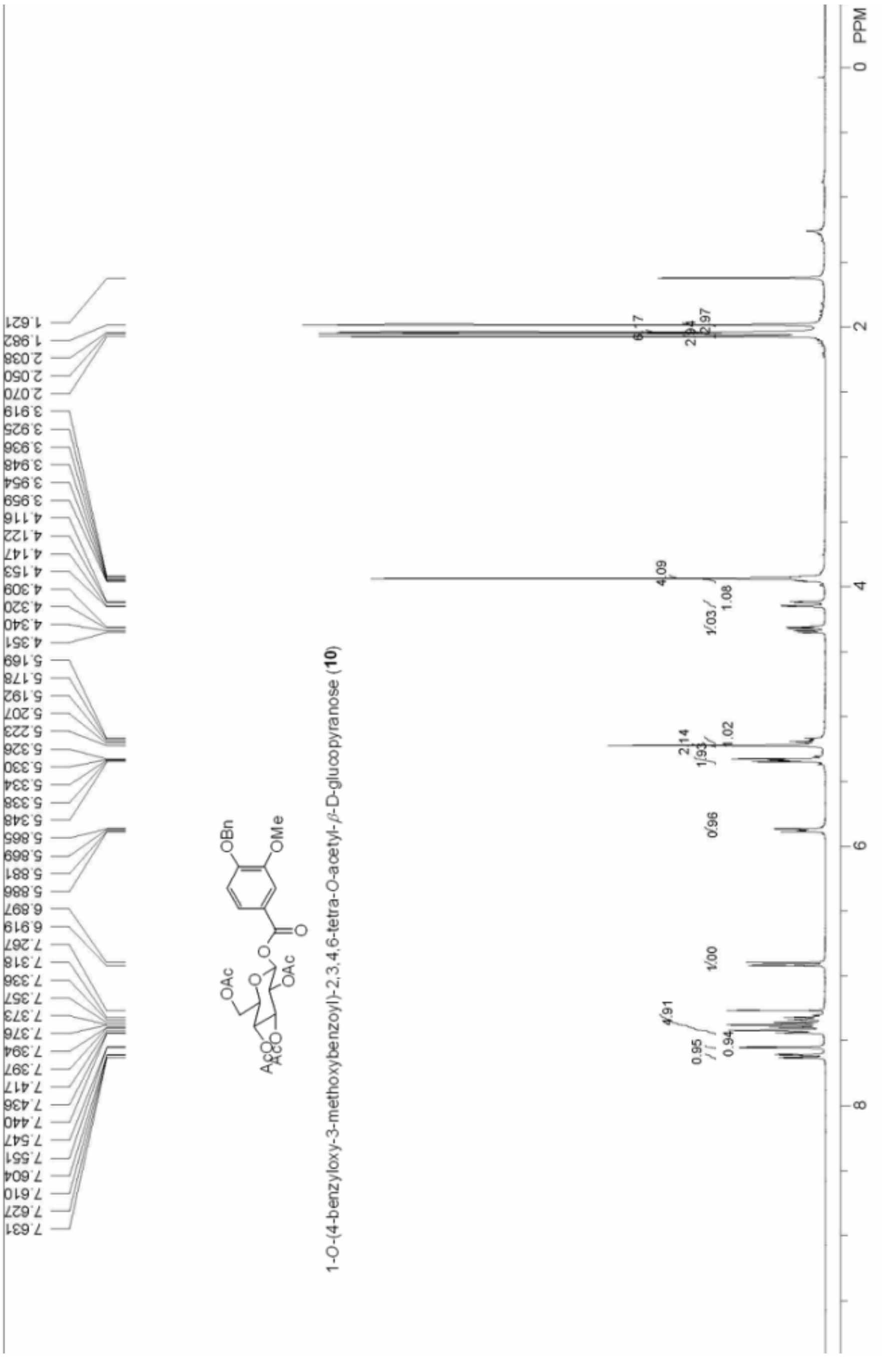

Figure S7. ^{1}H NMR spectrum of compound **10**

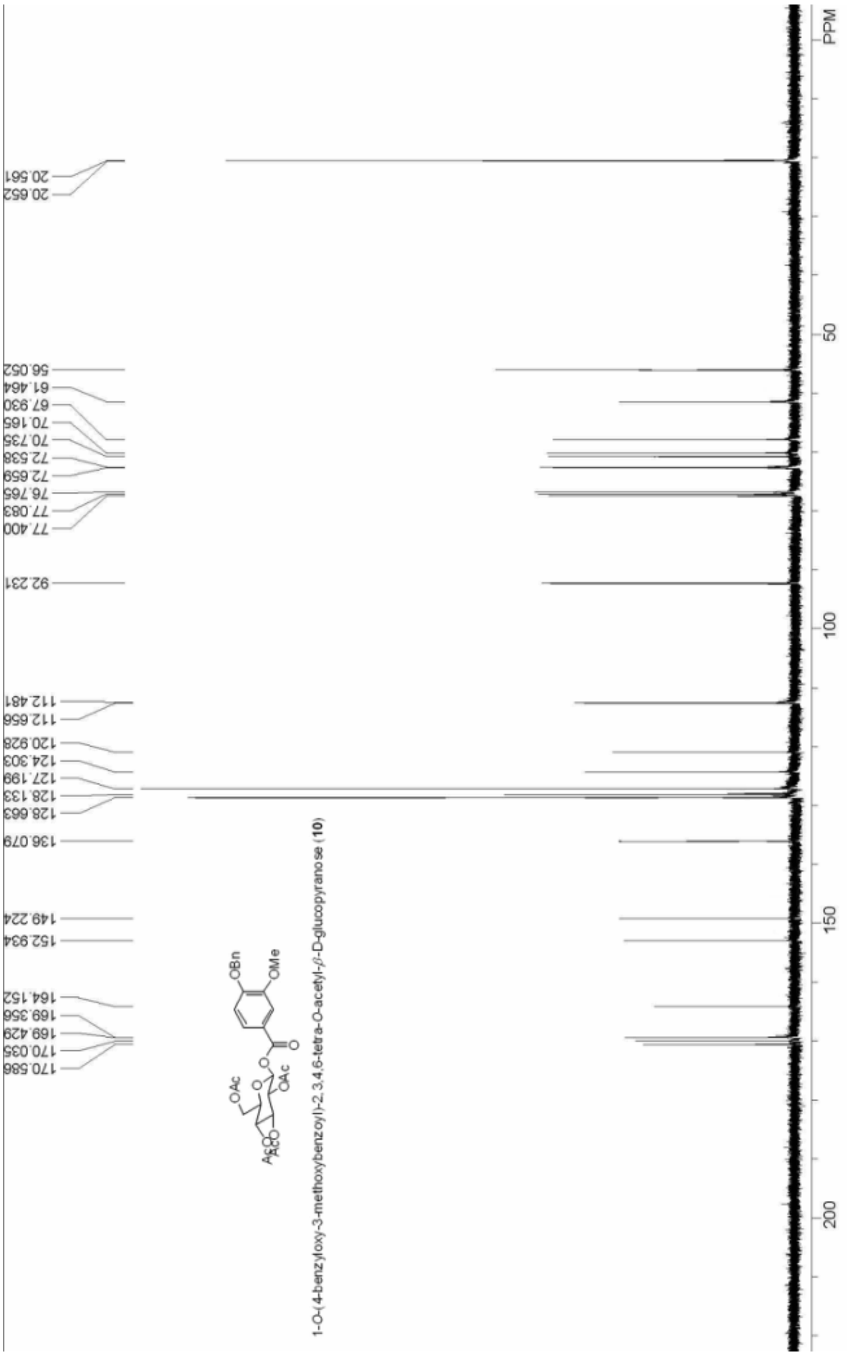

Figure S8. ^{13}C NMR spectrum of compound **10**

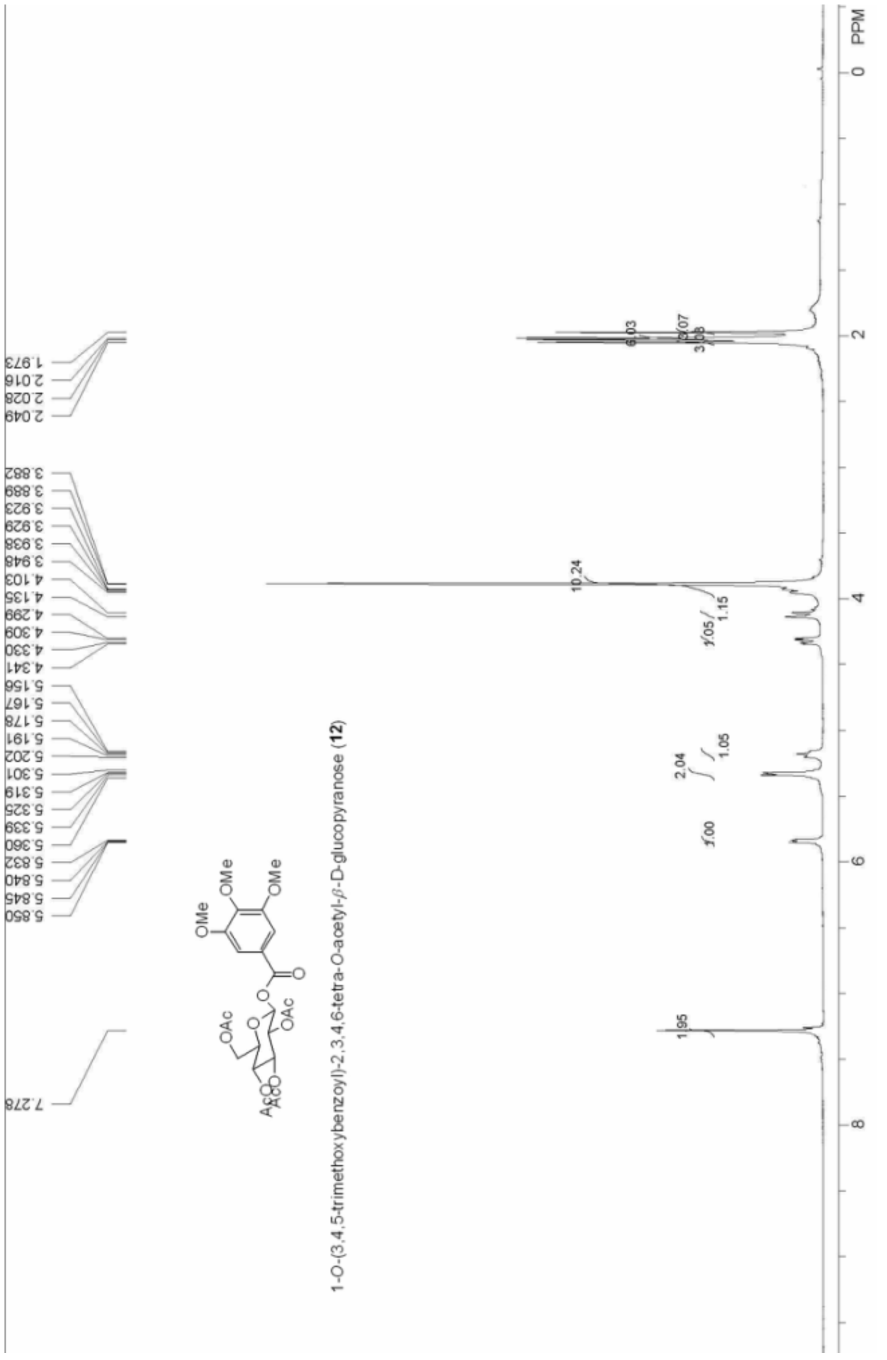

Figure S9. ^{1}H NMR spectrum of compound **12**

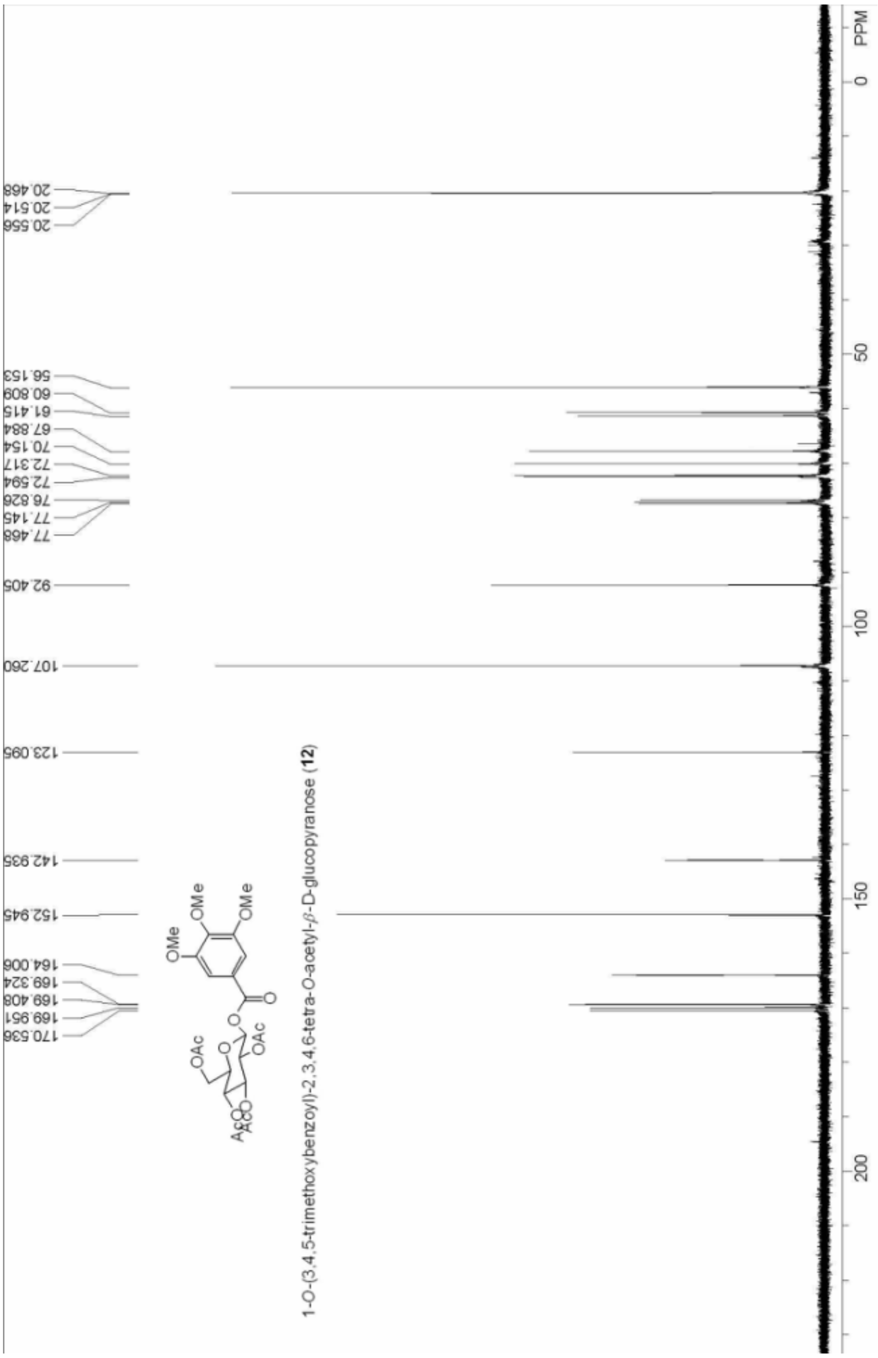

Figure S10. ^{13}C NMR spectrum of compound **12**

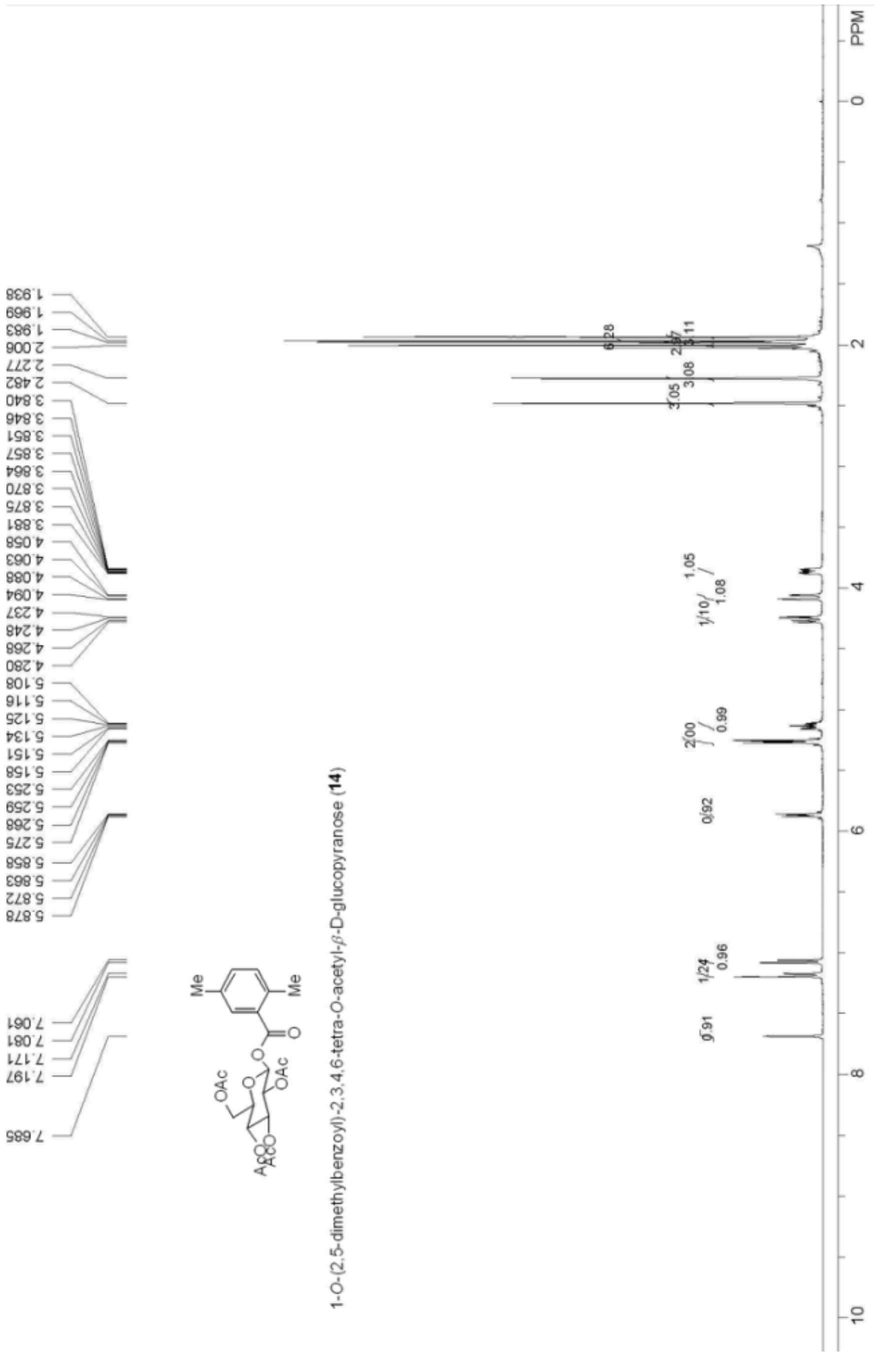

Figure S11. ^{1}H NMR spectrum of compound **14**

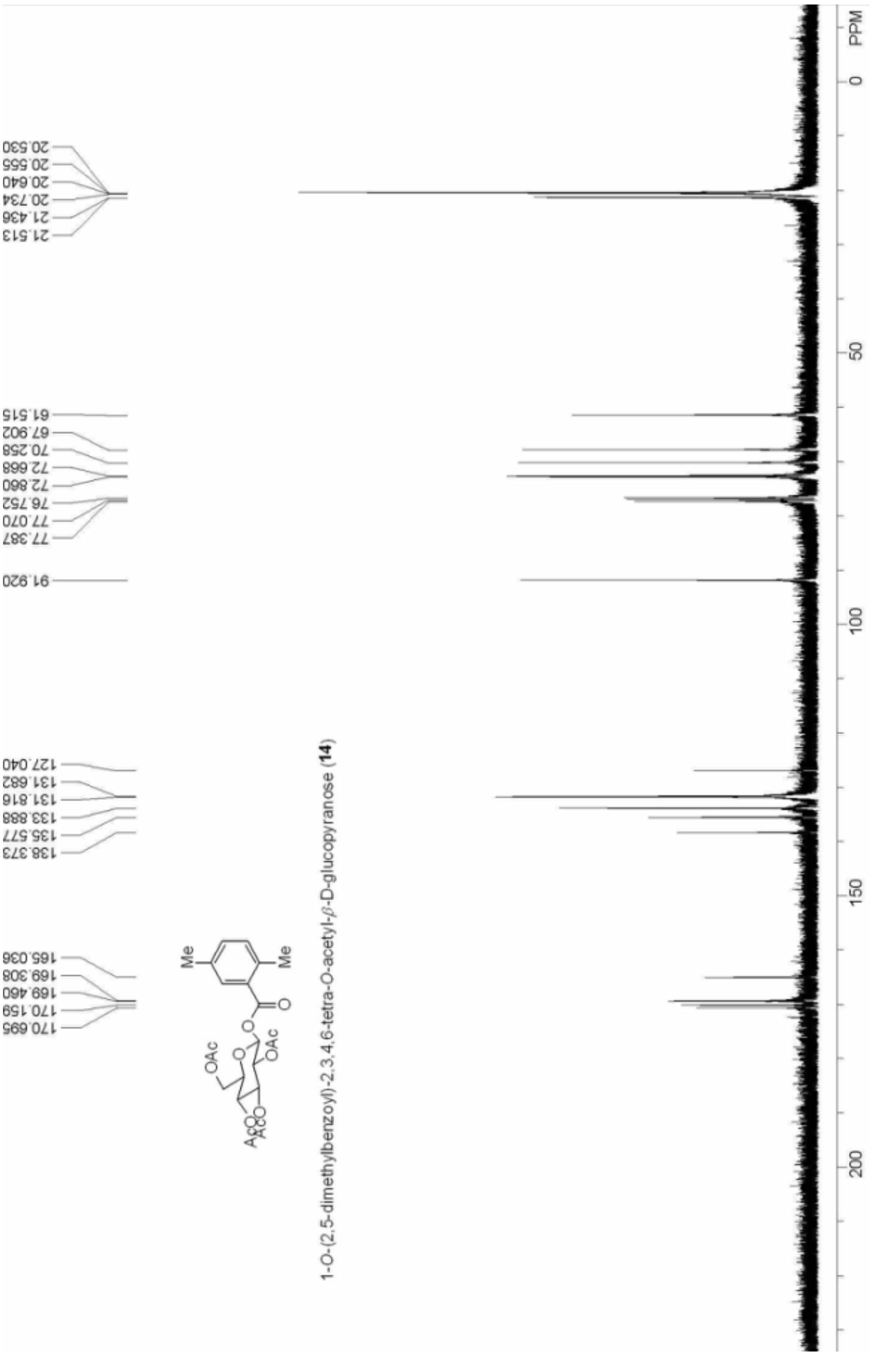

Figure S12. ^{13}C NMR spectrum of compound **14**

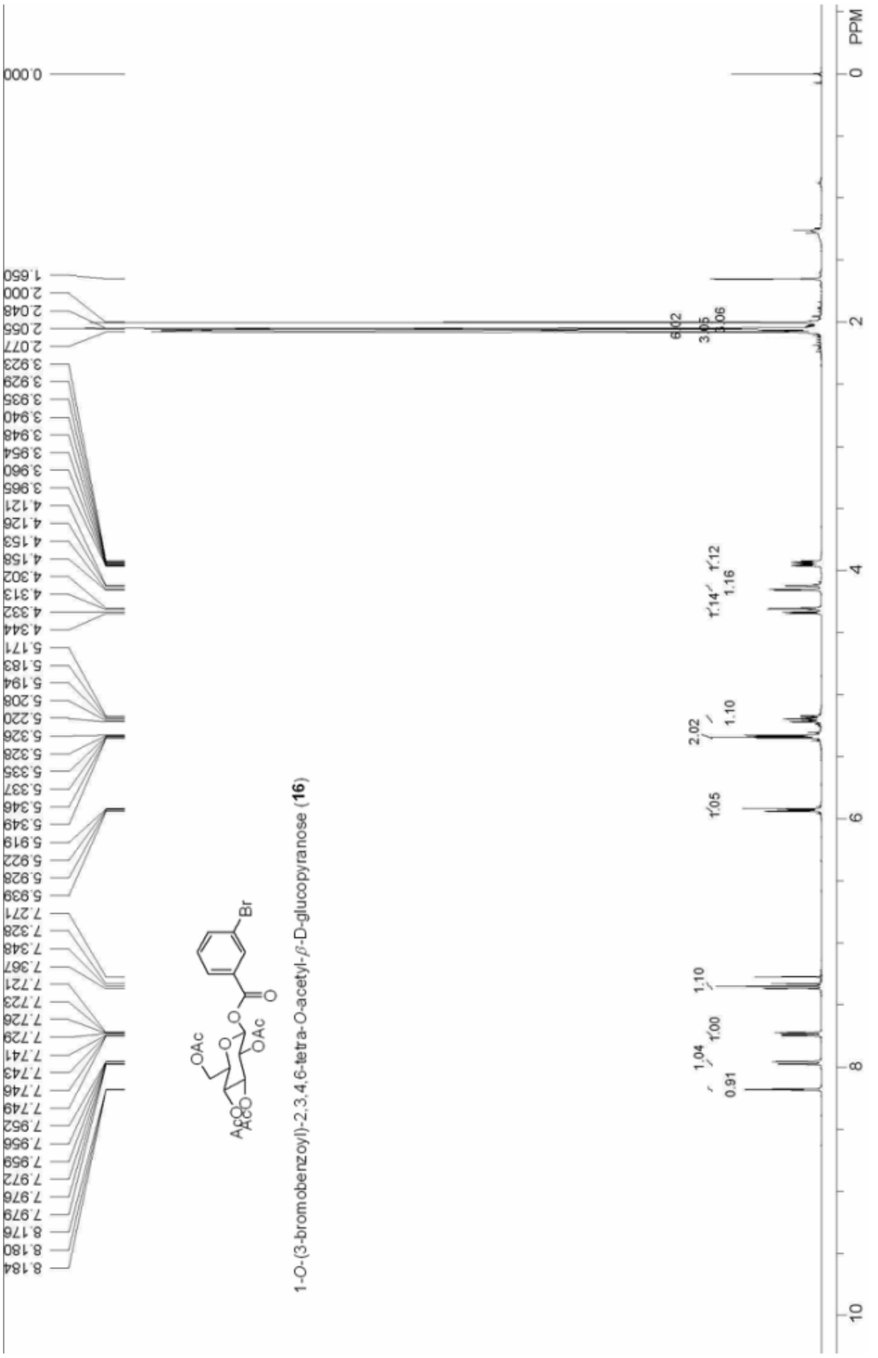

Figure S13. ^{1}H NMR spectrum of compound **16**

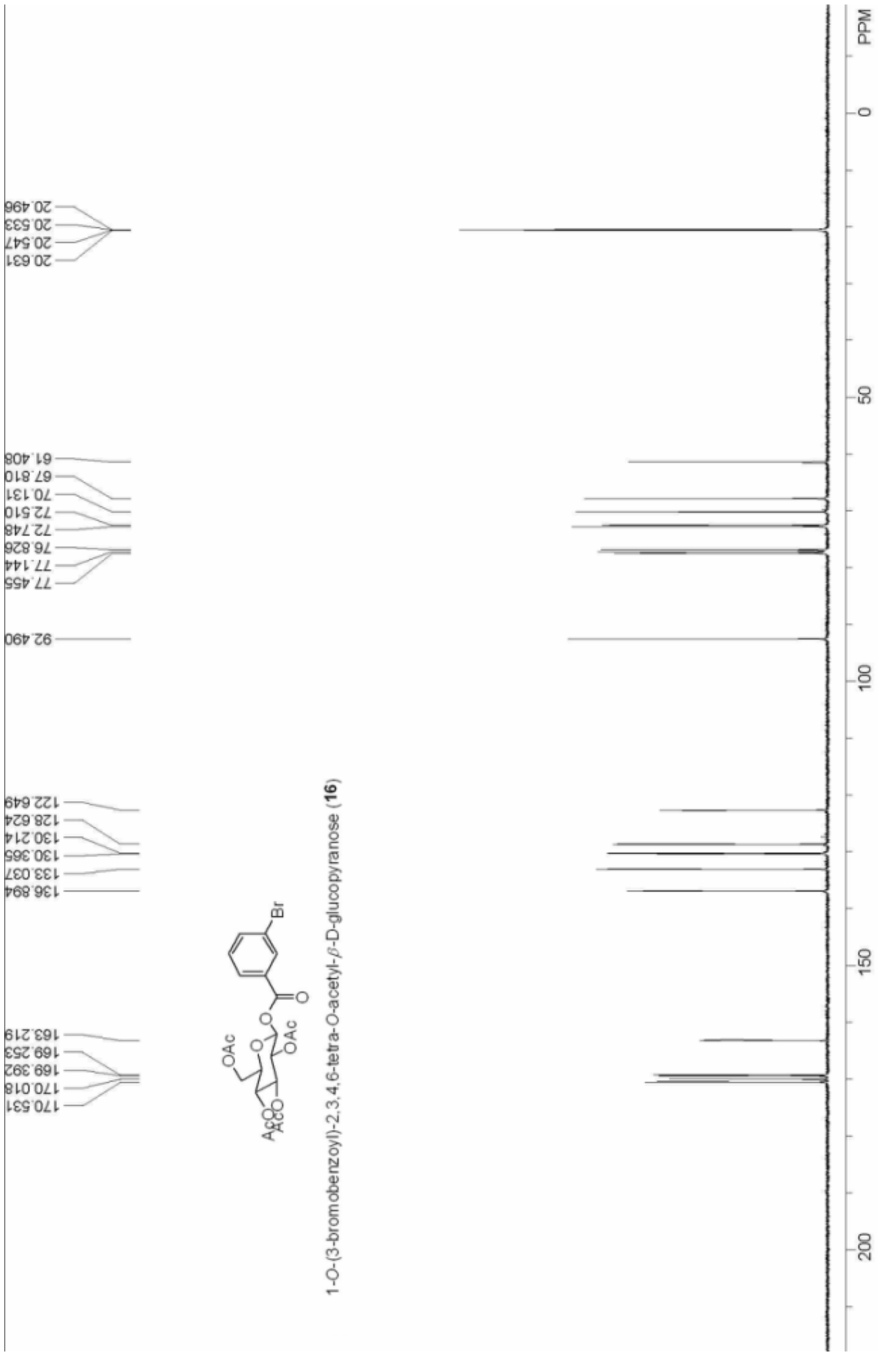

Figure S14. ^{13}C NMR spectrum of compound **16**

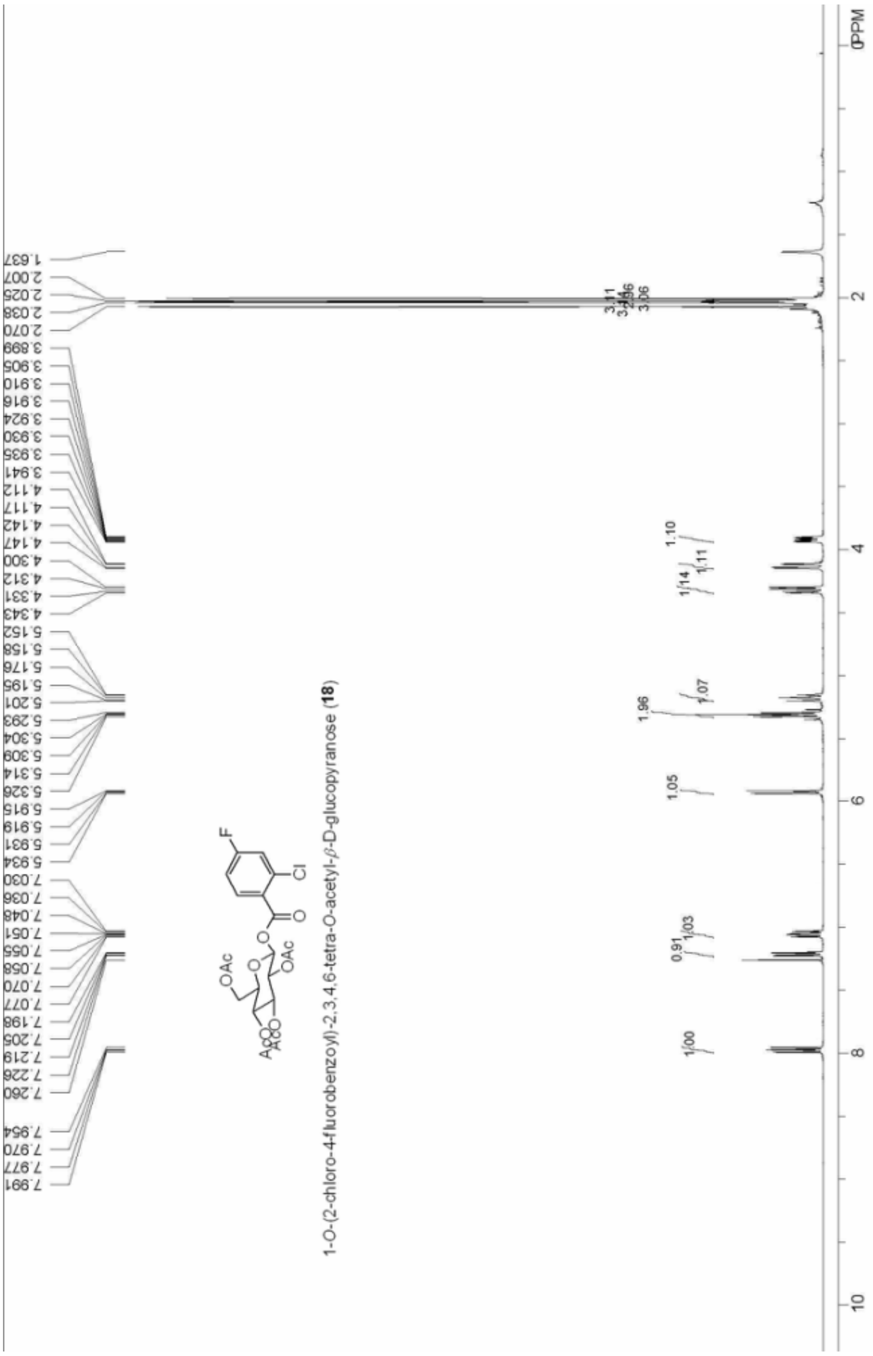

Figure S15. ^{1}H NMR spectrum of compound **18**

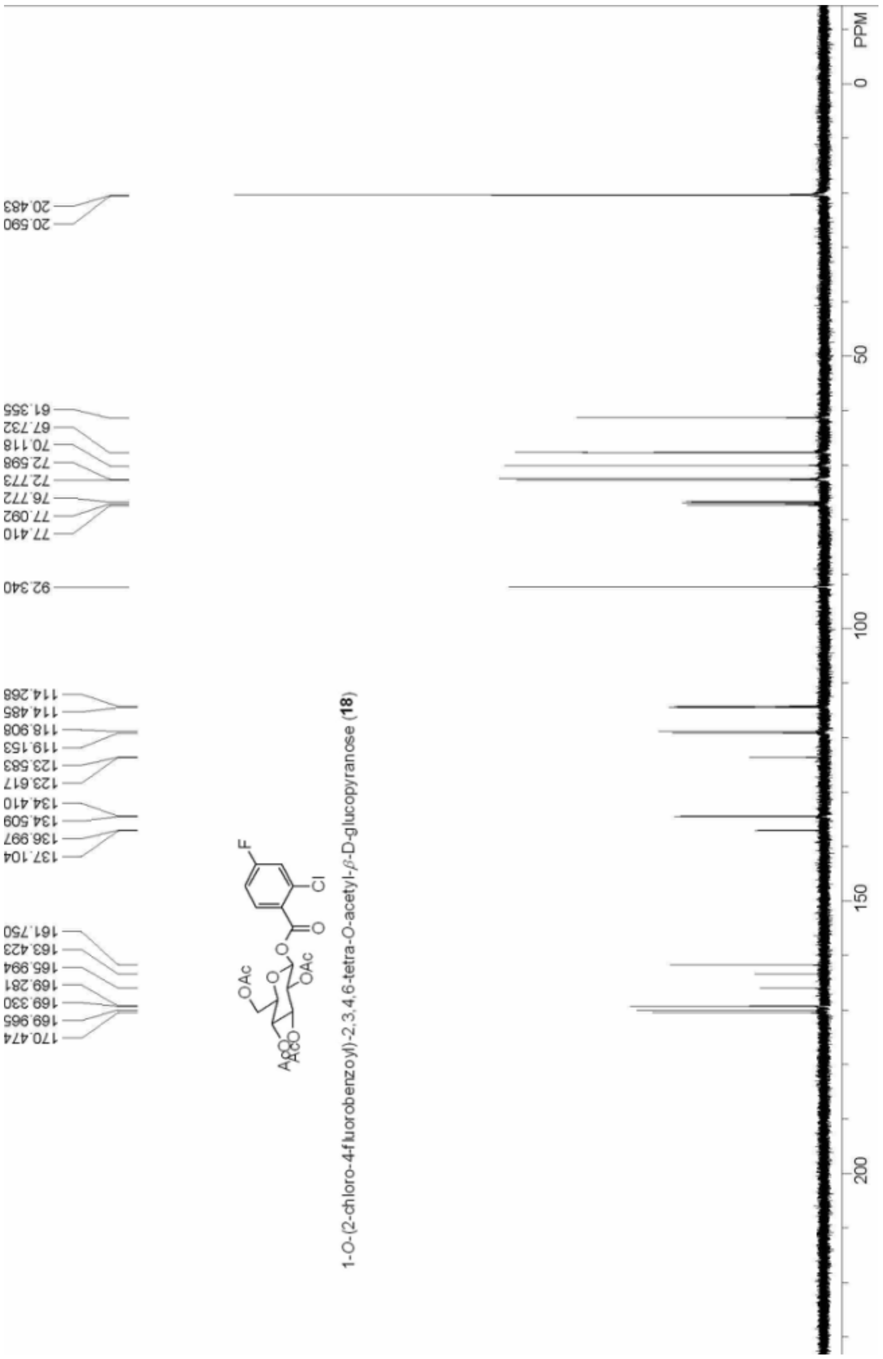

Figure S16. ^{13}C NMR spectrum of compound **18**

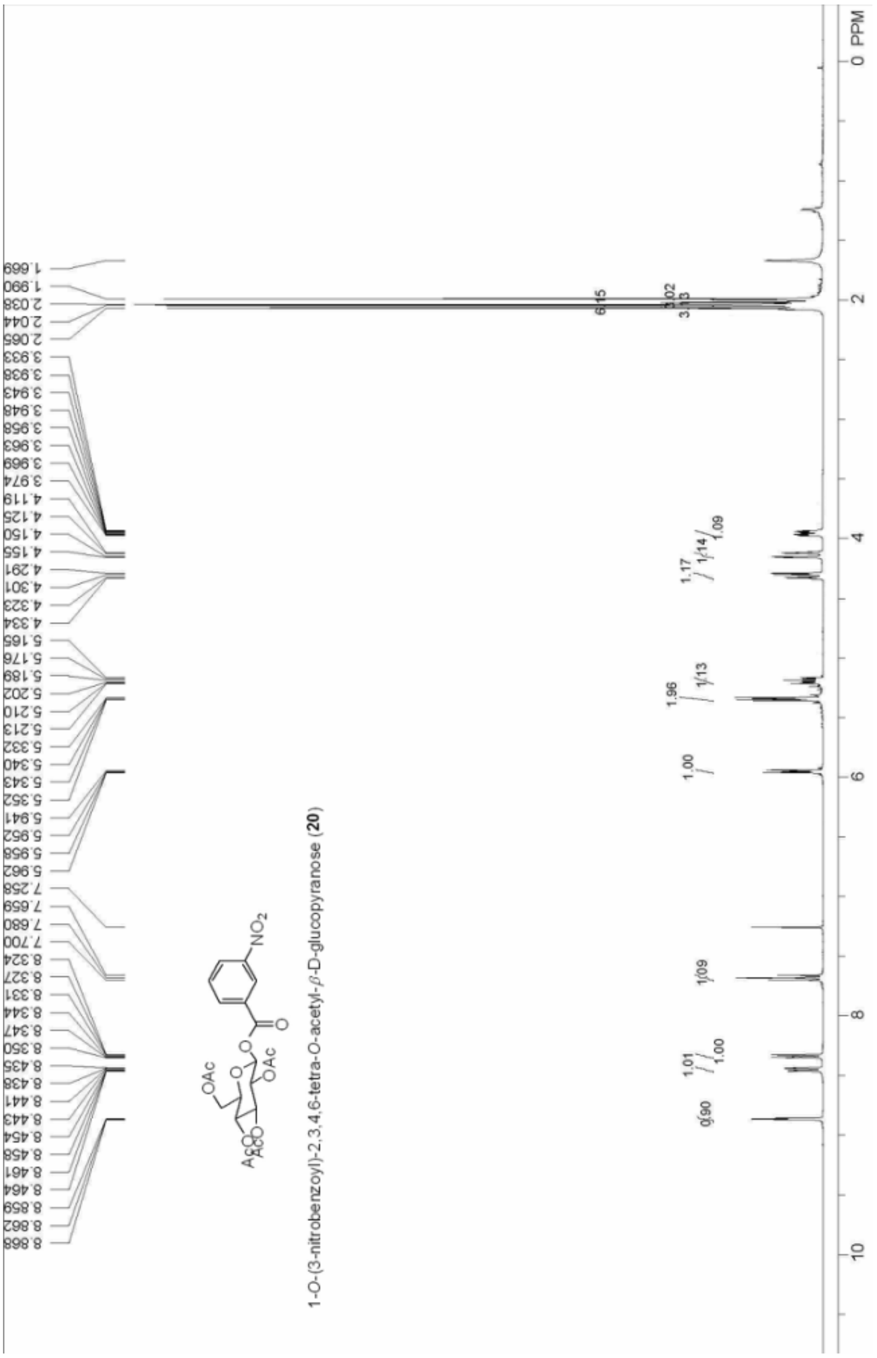

Figure S17. ^{1}H NMR spectrum of compound **20**

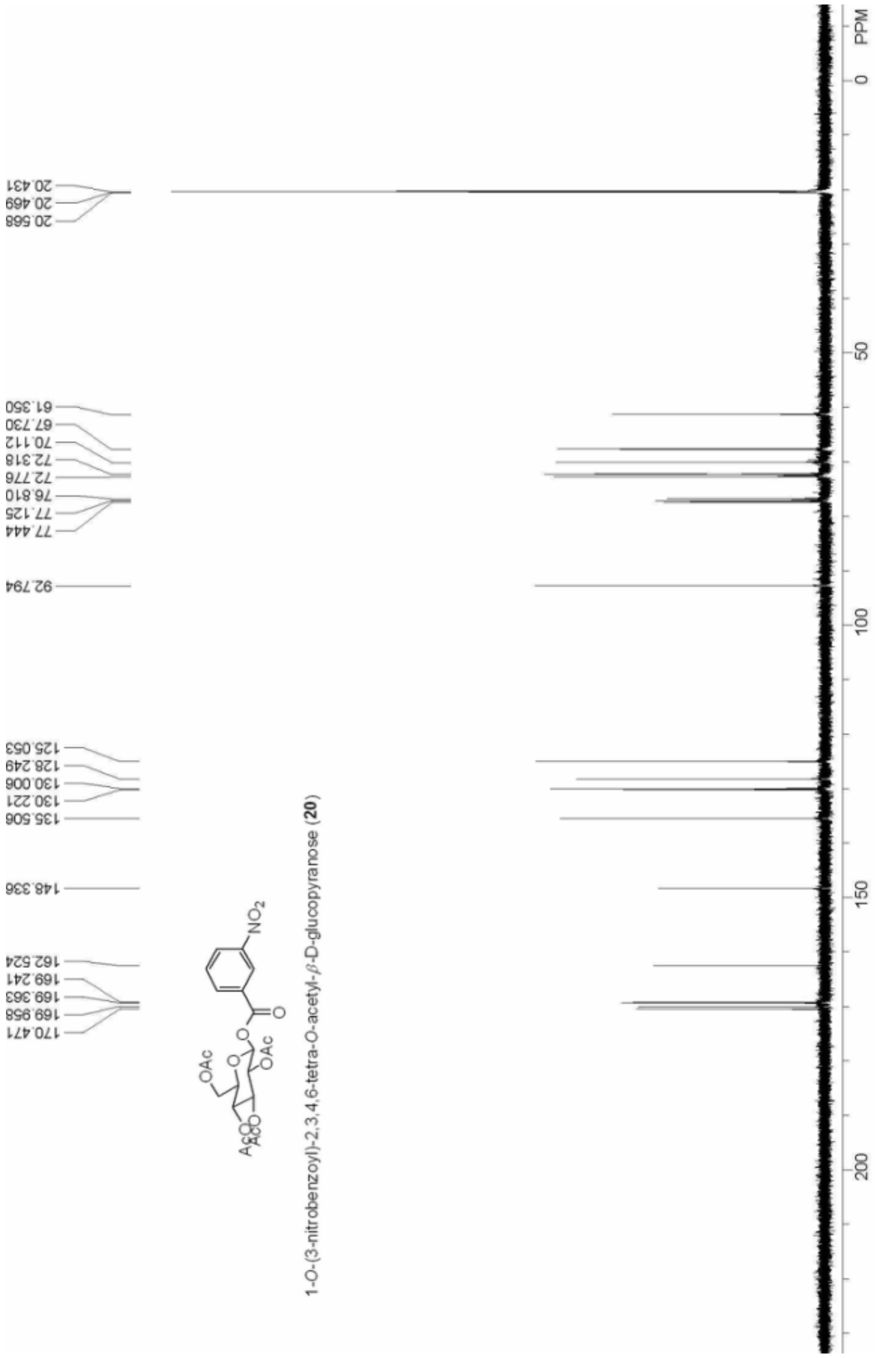

Figure S18. ^{13}C NMR spectrum of compound **20**

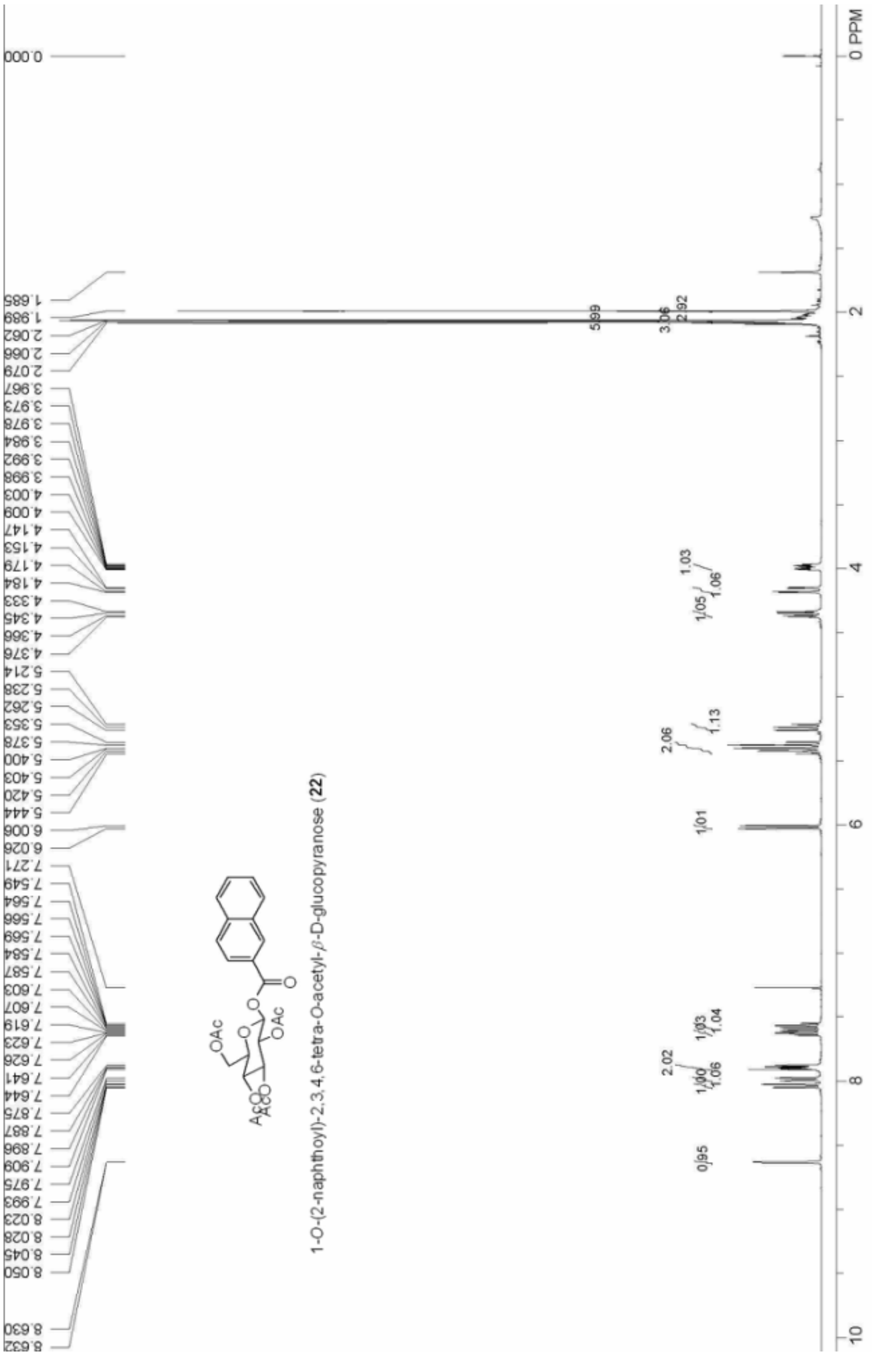

Figure S19. ^{1}H NMR spectrum of compound **22**

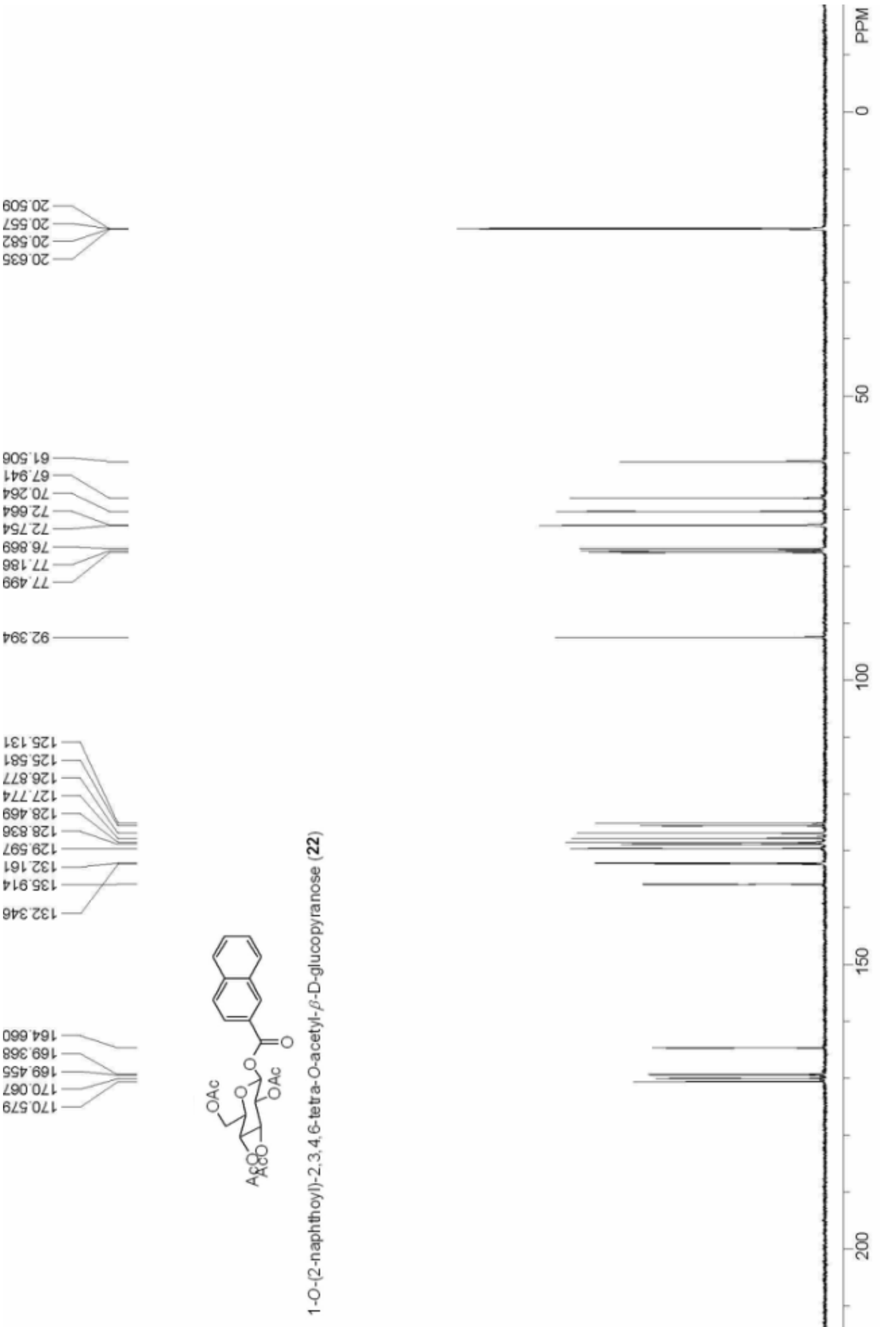

Figure S20. ^{13}C NMR spectrum of compound **22**

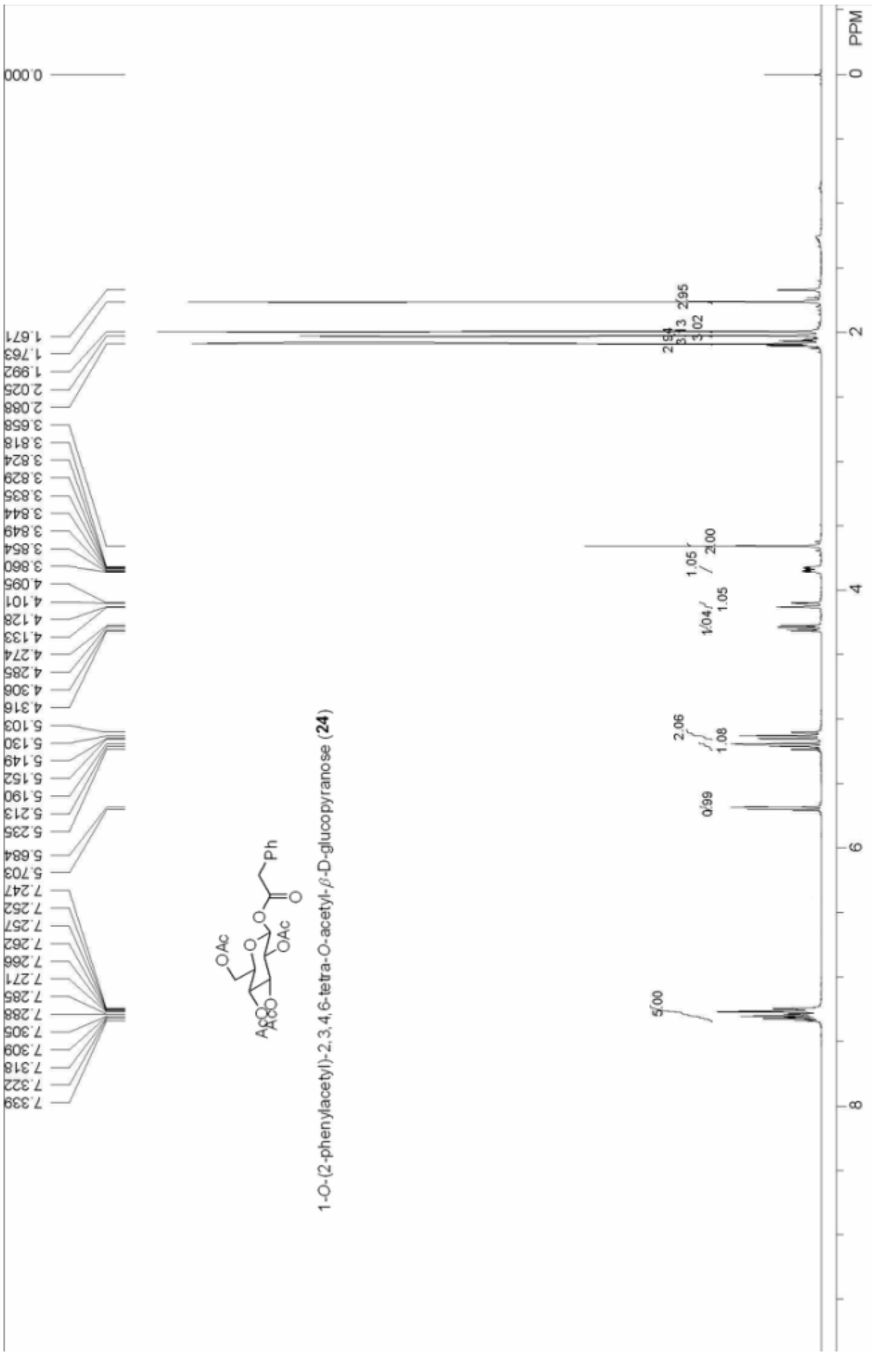

Figure S21. ^{1}H NMR spectrum of compound **24**

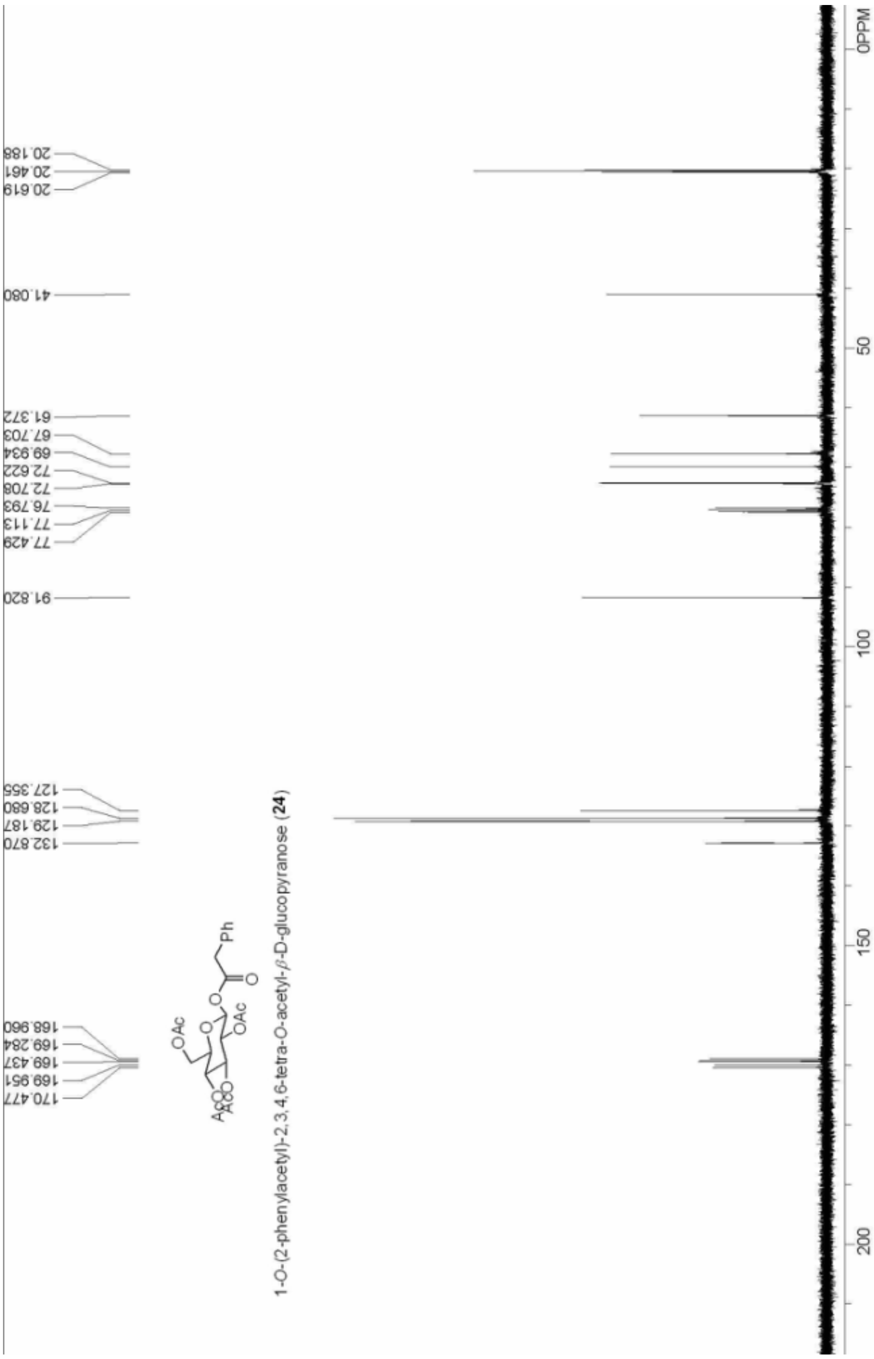

Figure S22. ^{13}C NMR spectrum of compound **24**

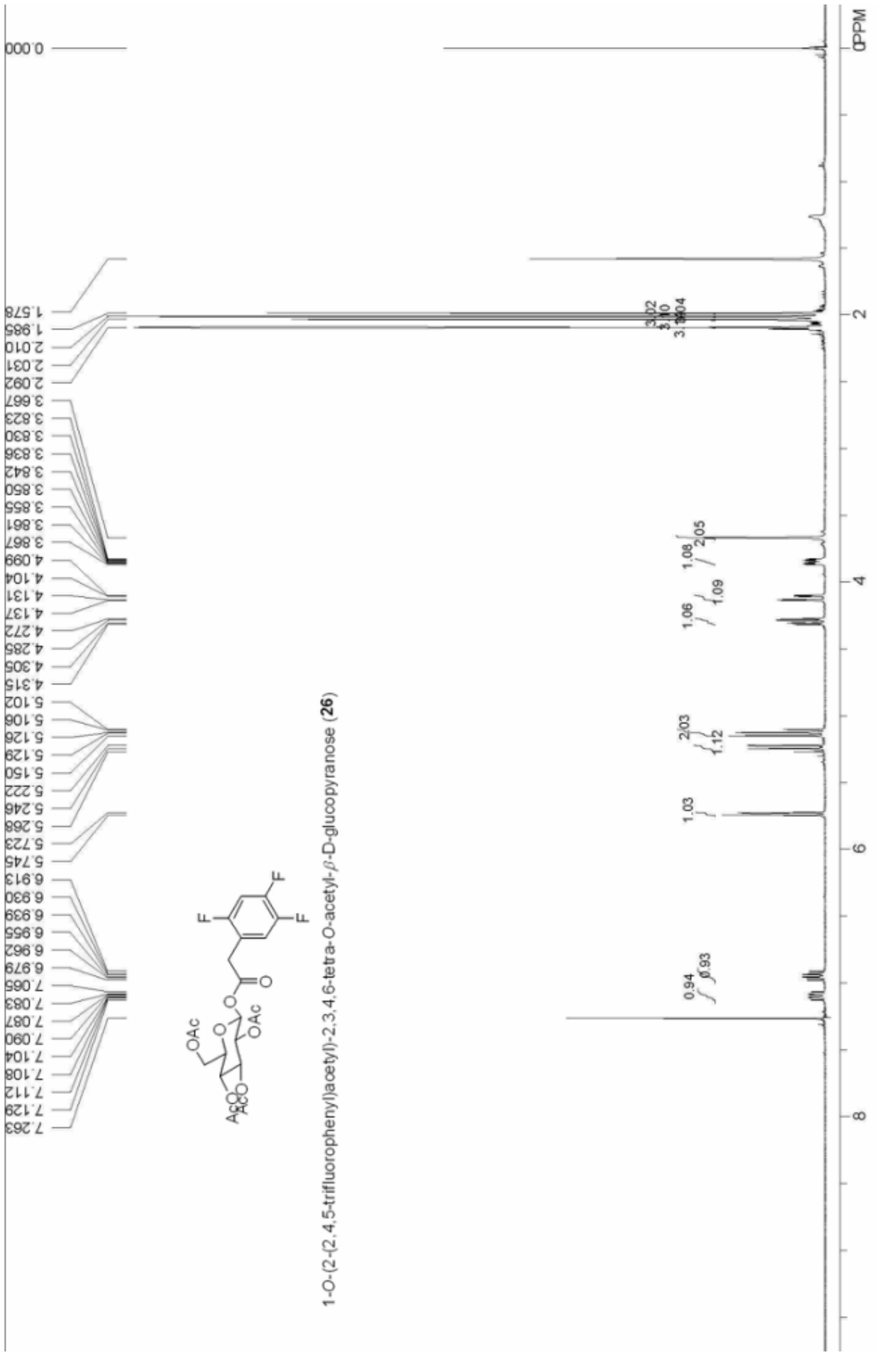

Figure S23. ^{1}H NMR spectrum of compound **26**

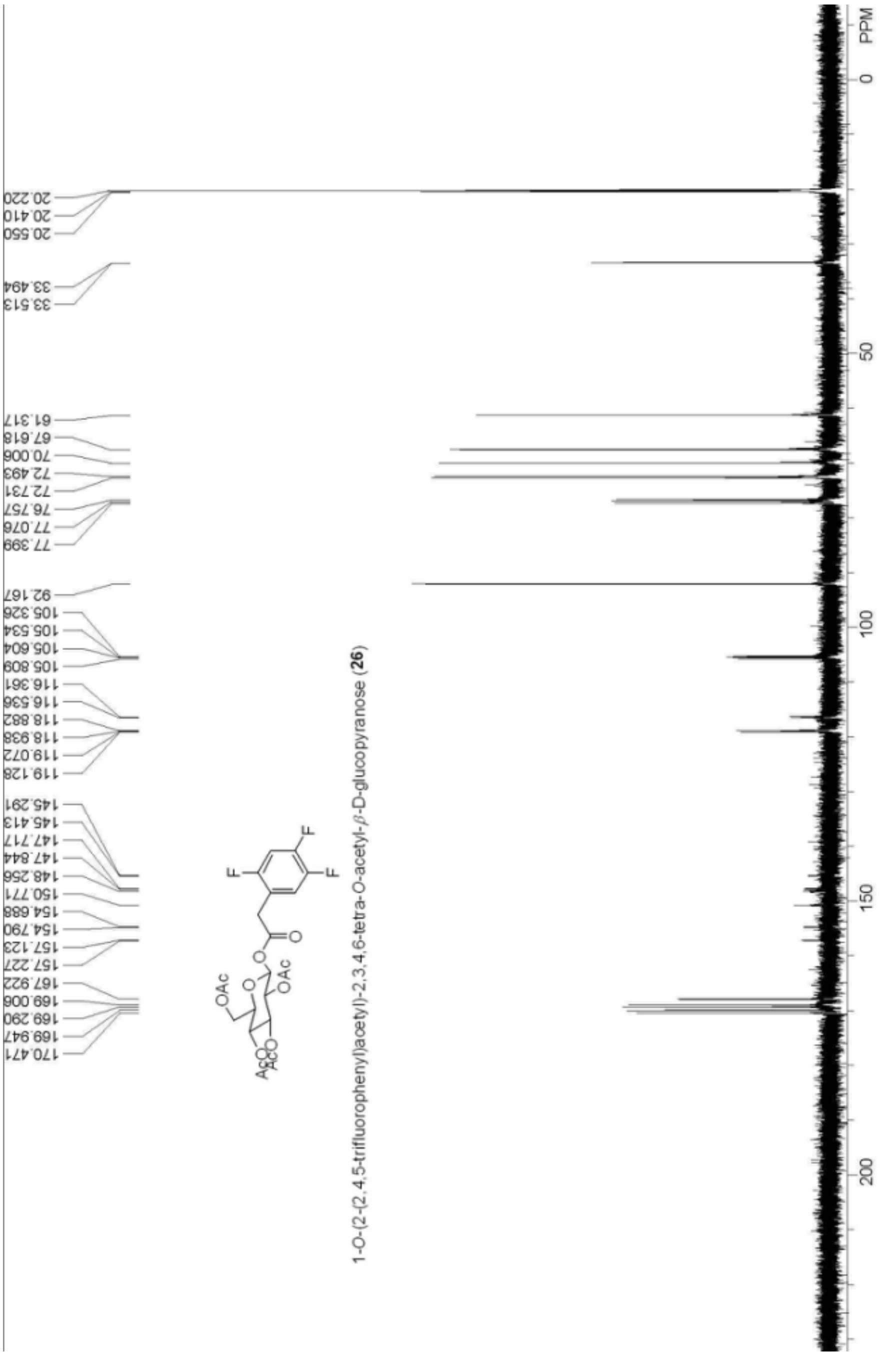

Figure S24. ^{13}C NMR spectrum of compound **26**

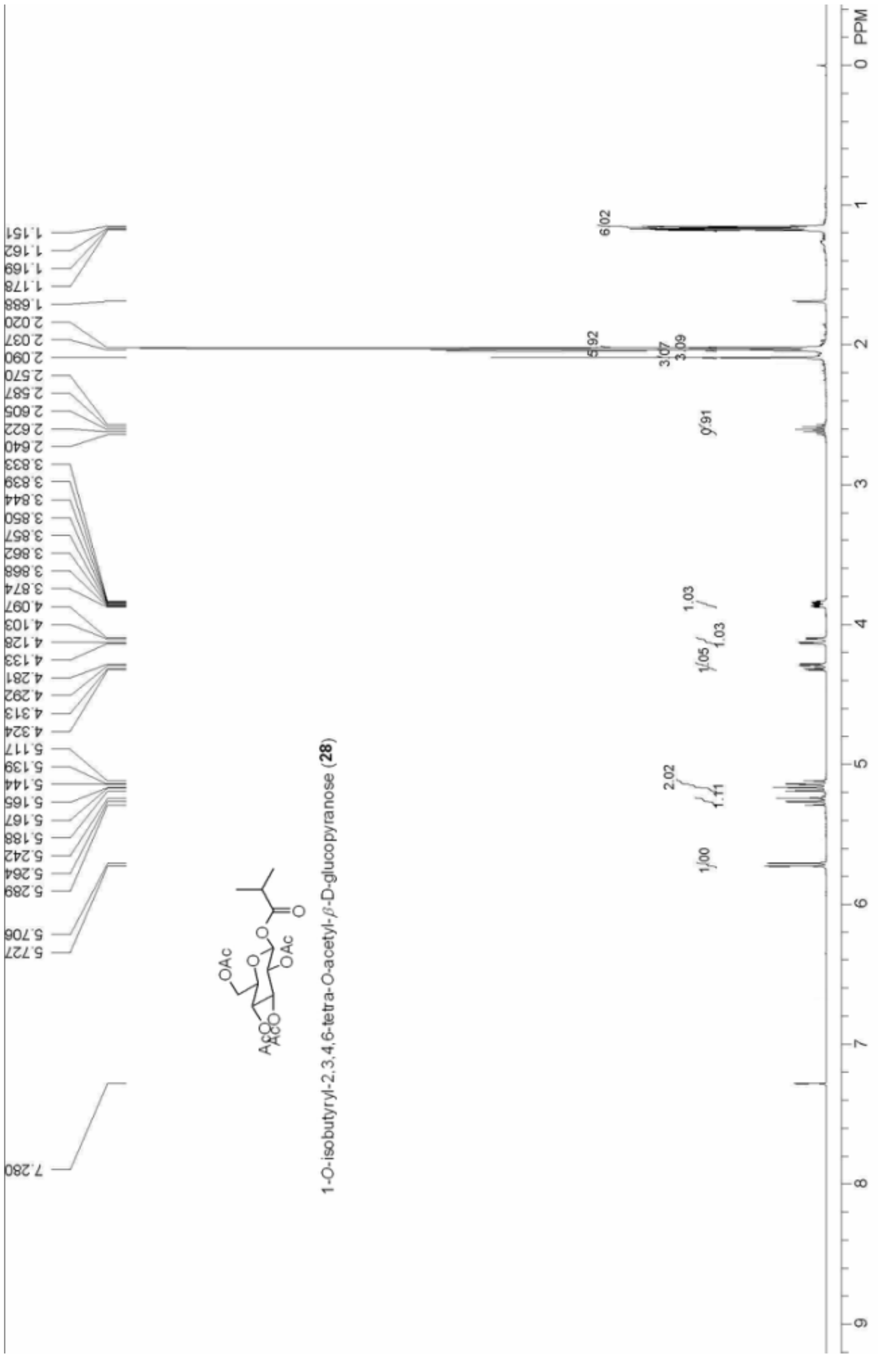

Figure S25. ^{1}H NMR spectrum of compound **28**

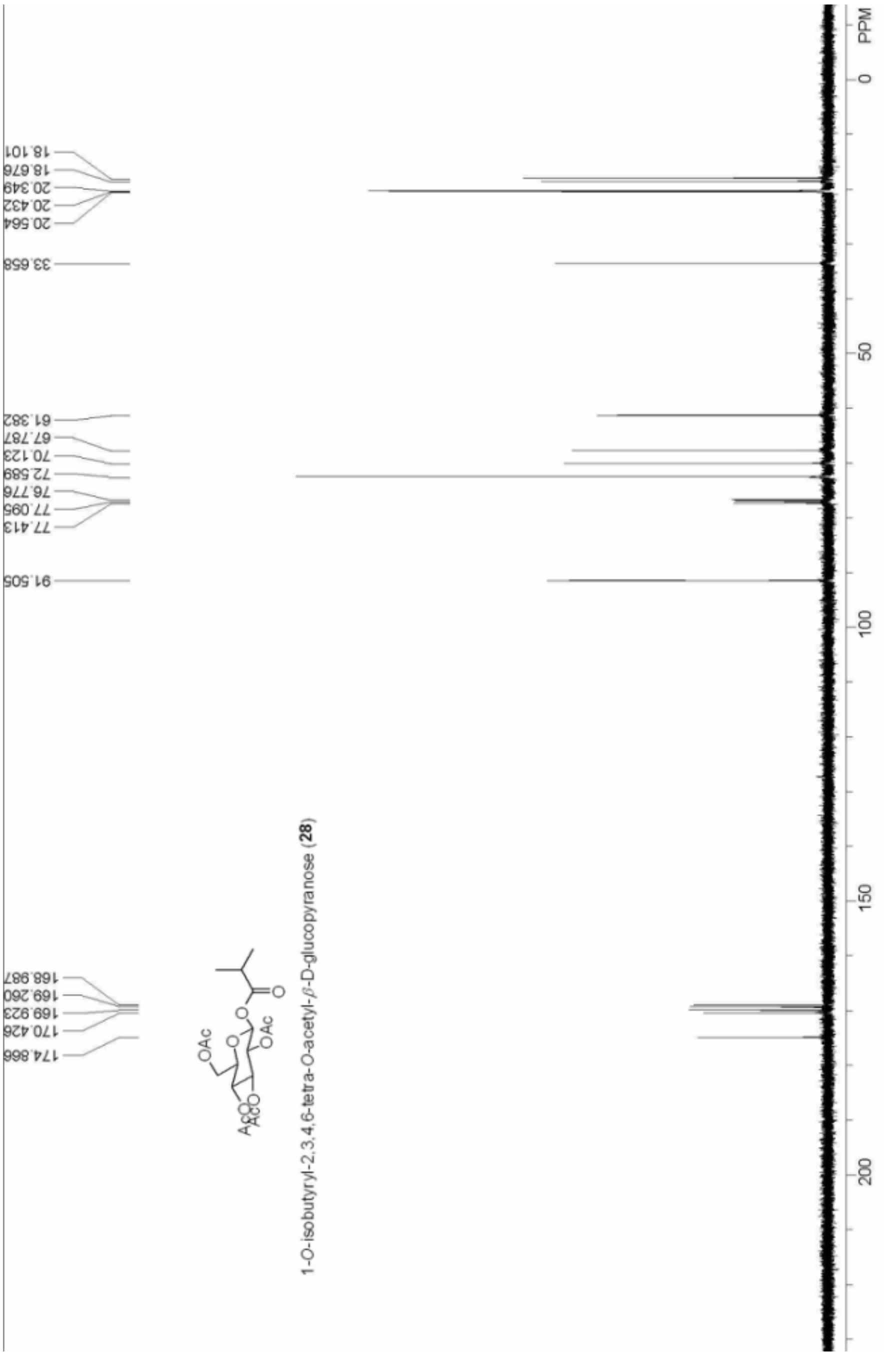

Figure S26. ^{13}C NMR spectrum of compound **28**

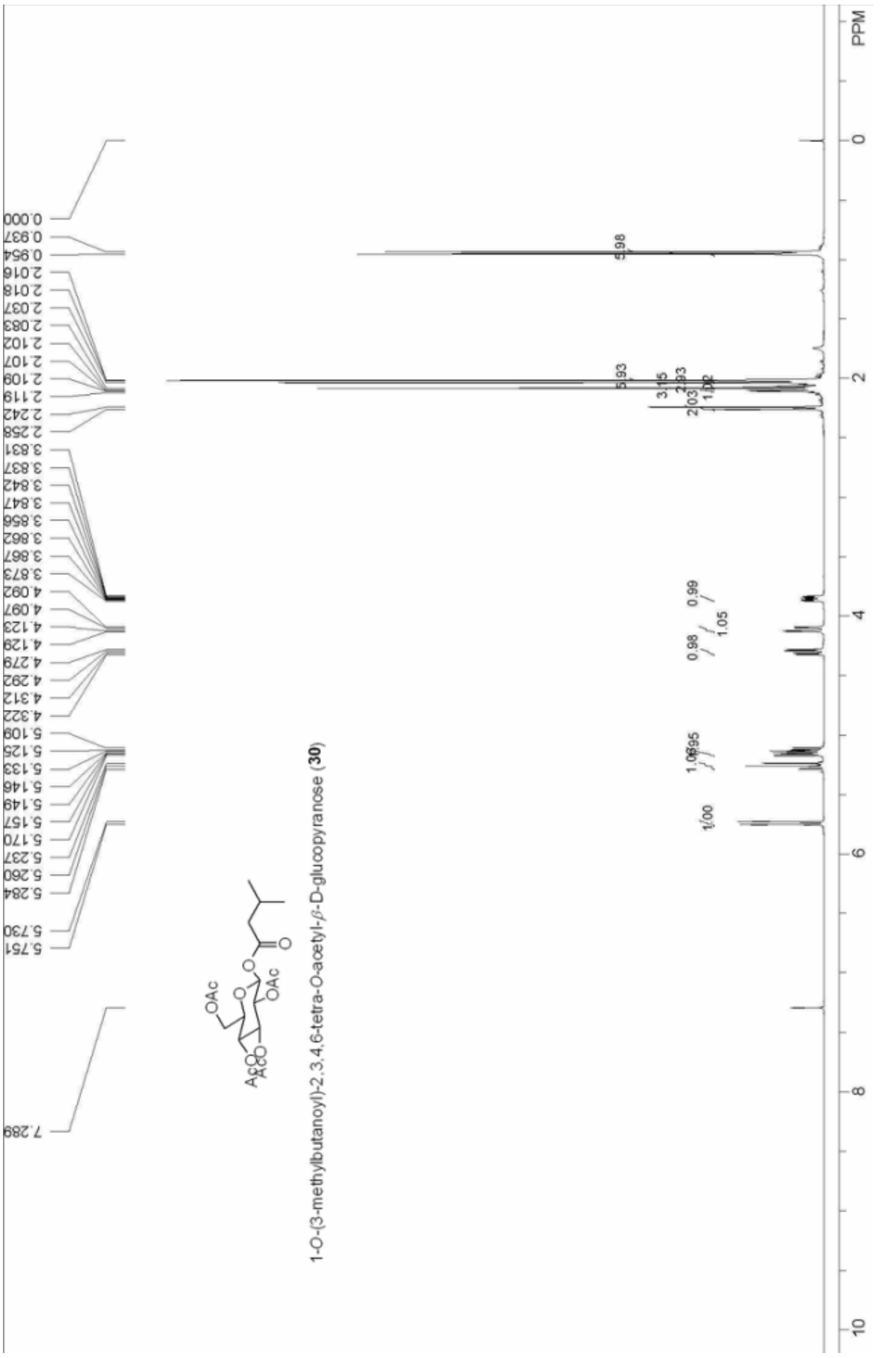

Figure S27. ^{1}H NMR spectrum of compound **30**

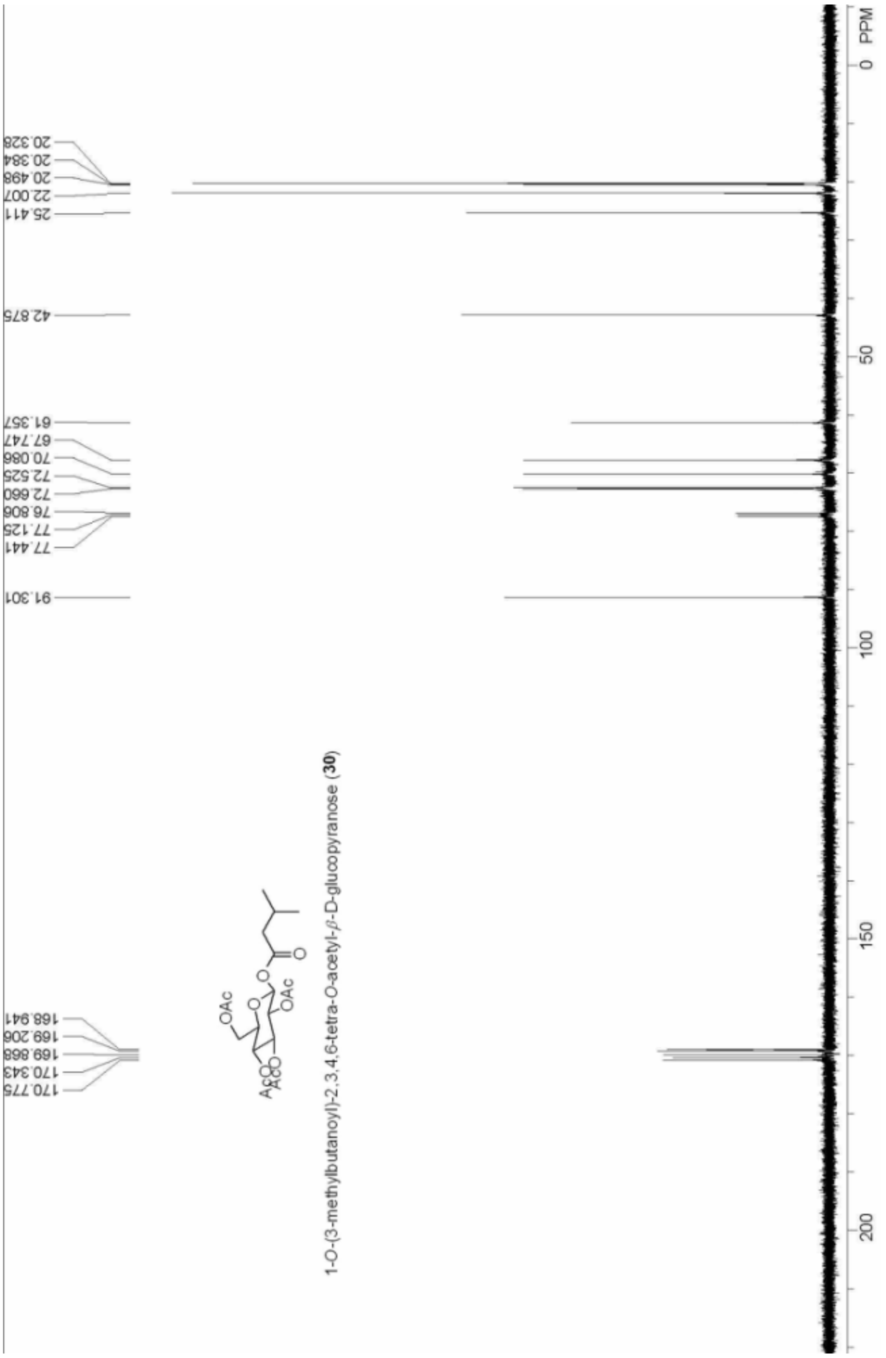

Figure S28. ^{13}C NMR spectrum of compound **30**

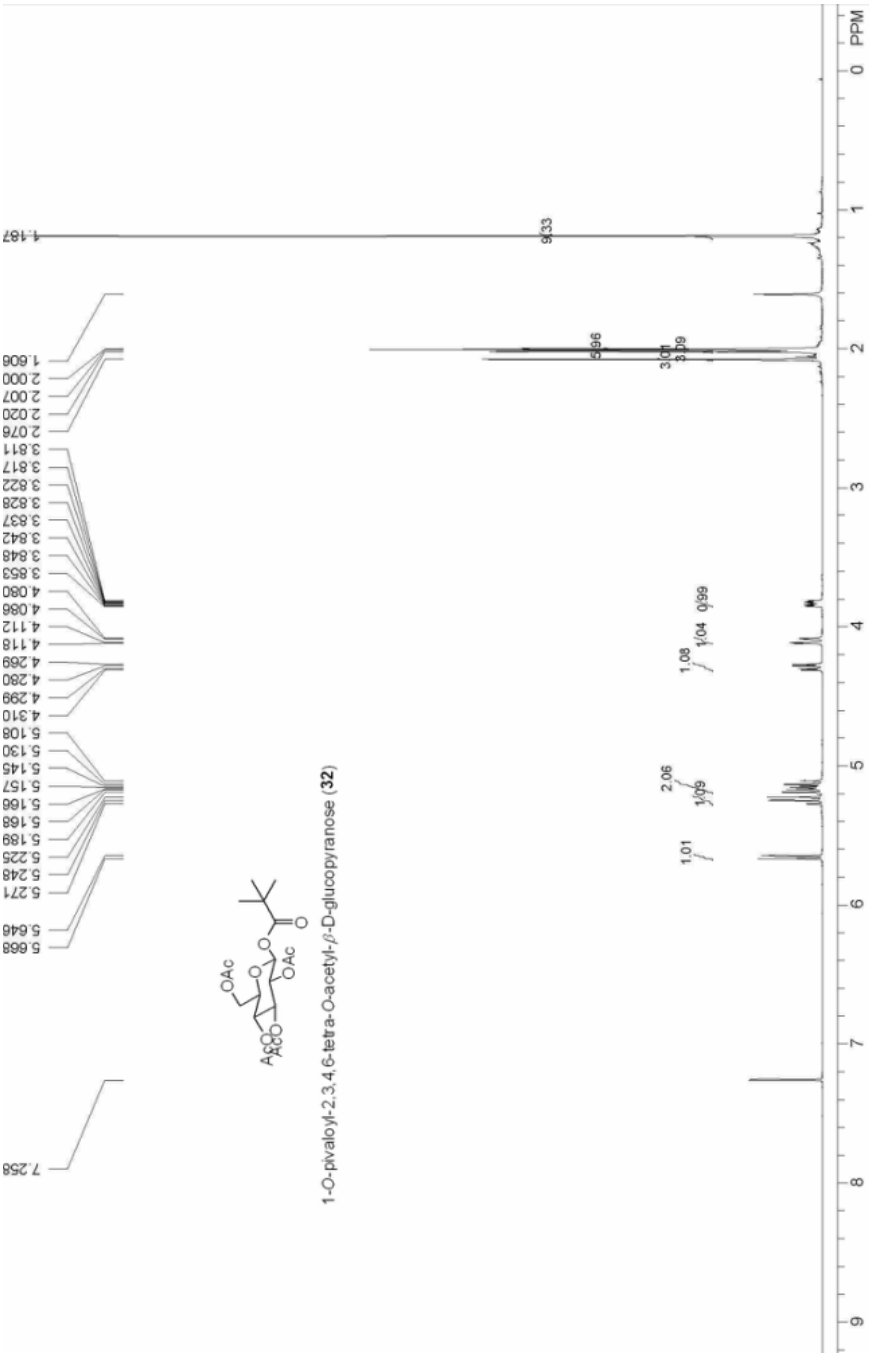

Figure S29. ^{1}H NMR spectrum of compound **32**

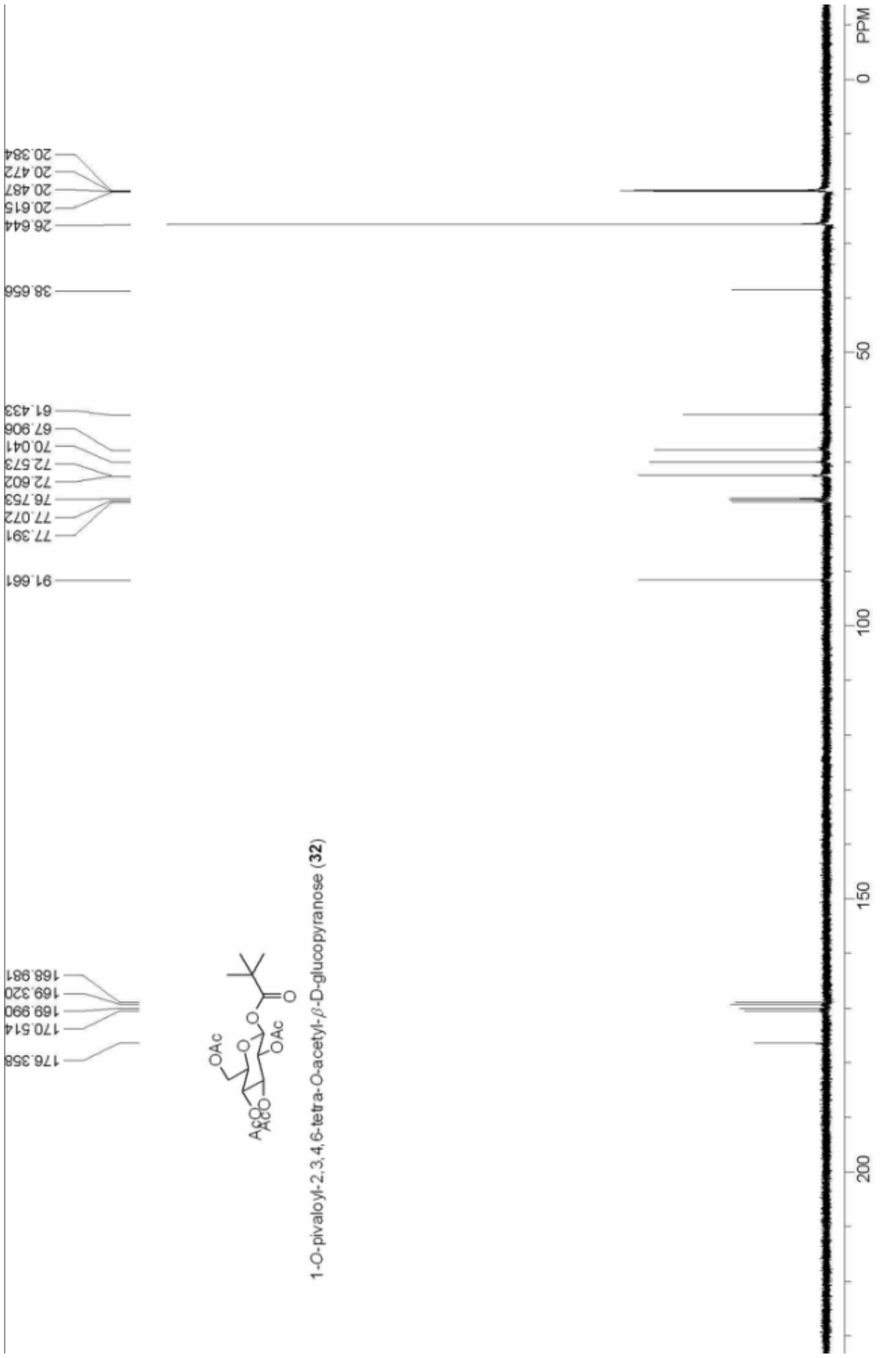

Figure S30. ^{13}C NMR spectrum of compound **32**

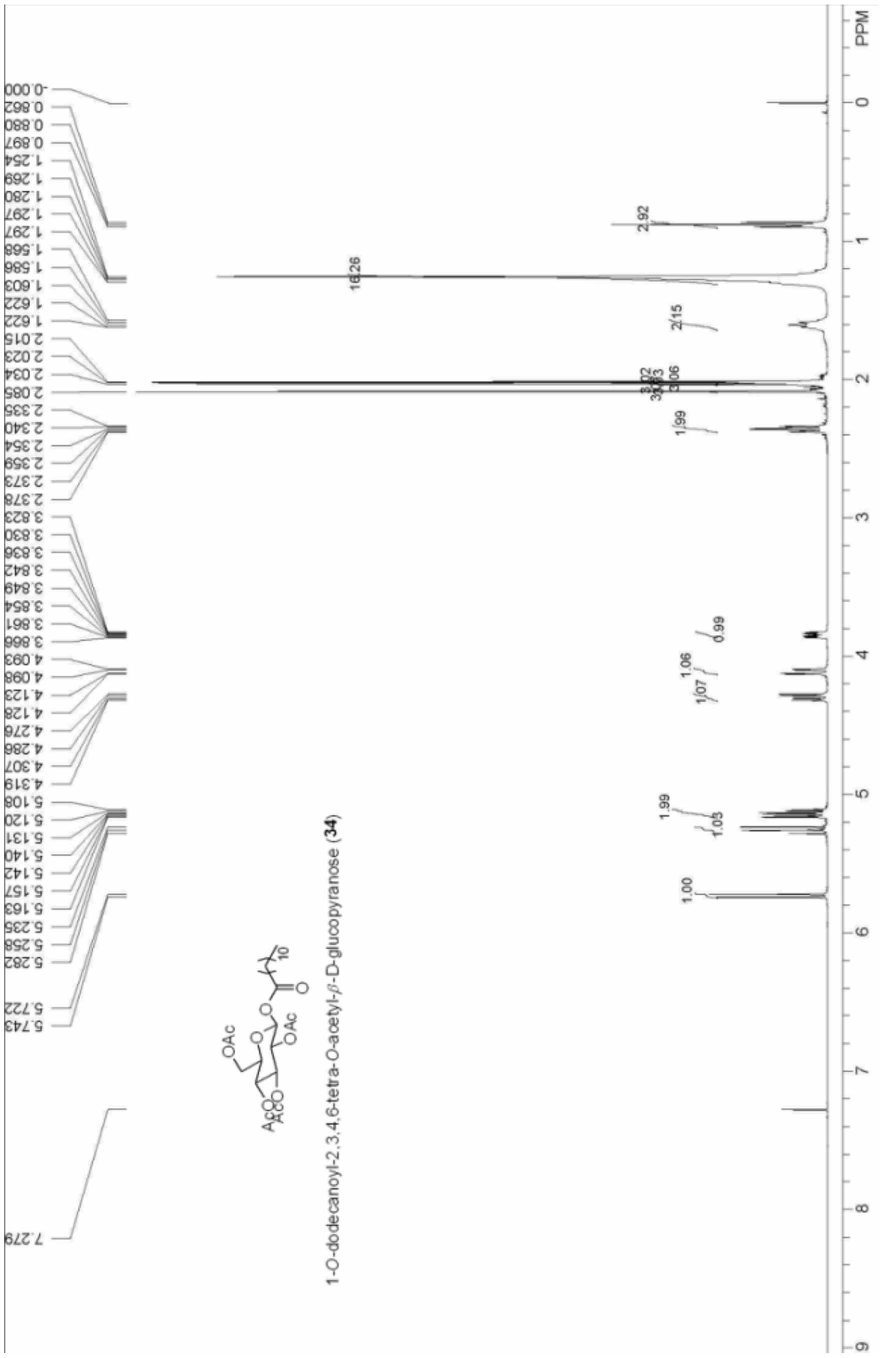

Figure S31. ^{1}H NMR spectrum of compound **34**

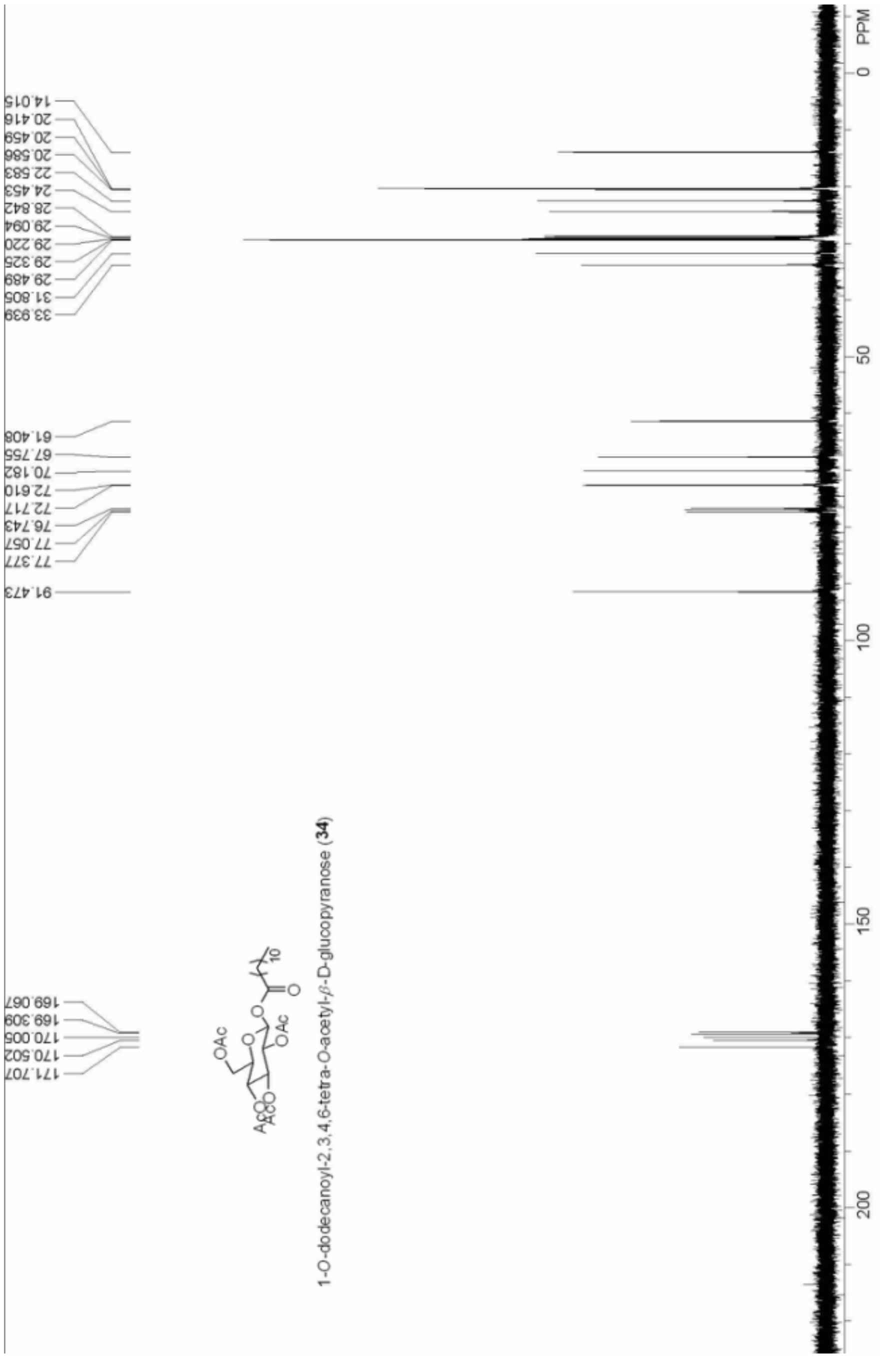

Figure S32. ^{13}C NMR spectrum of compound **34**

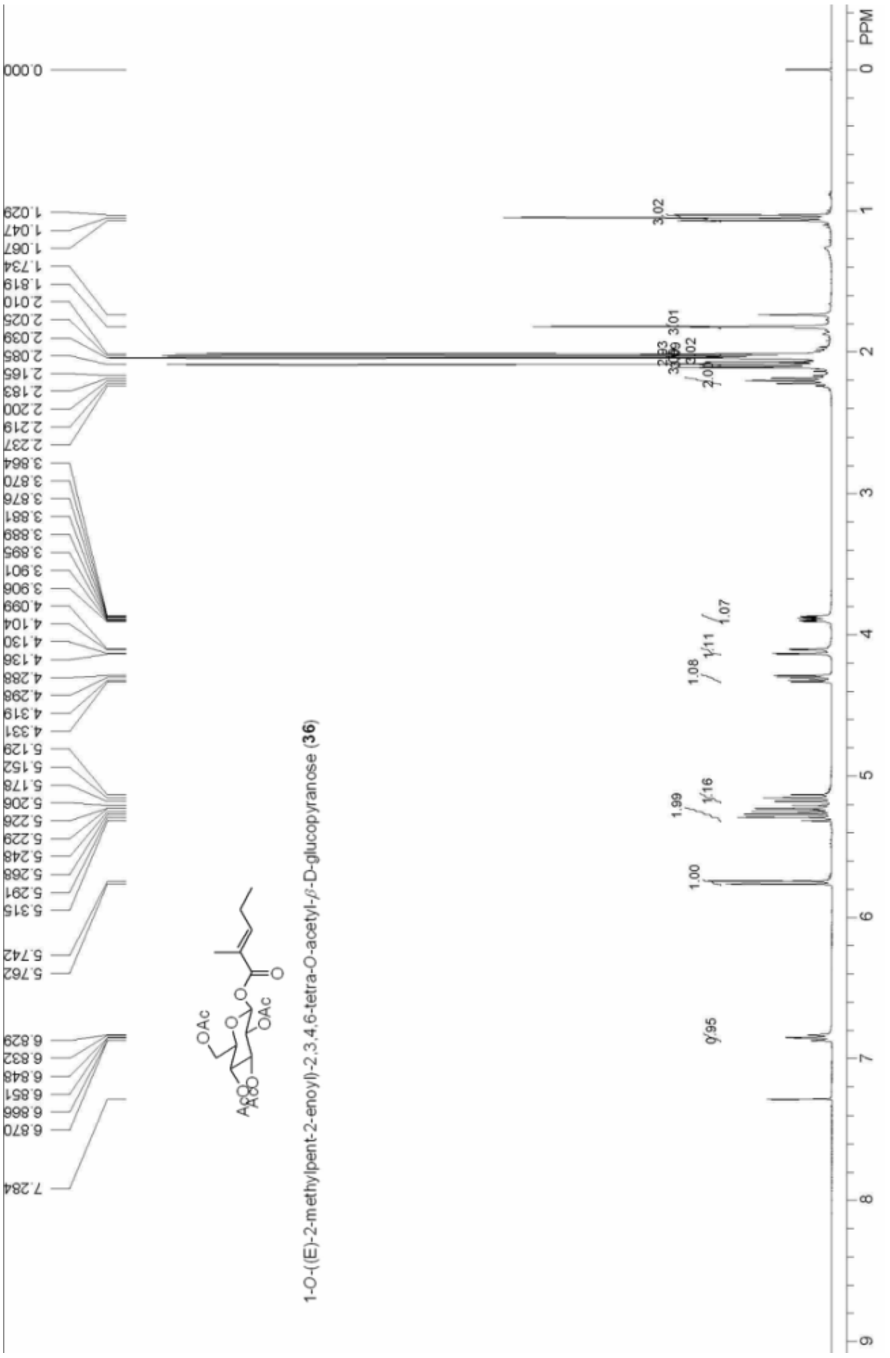

Figure S33. ^{1}H NMR spectrum of compound **36**

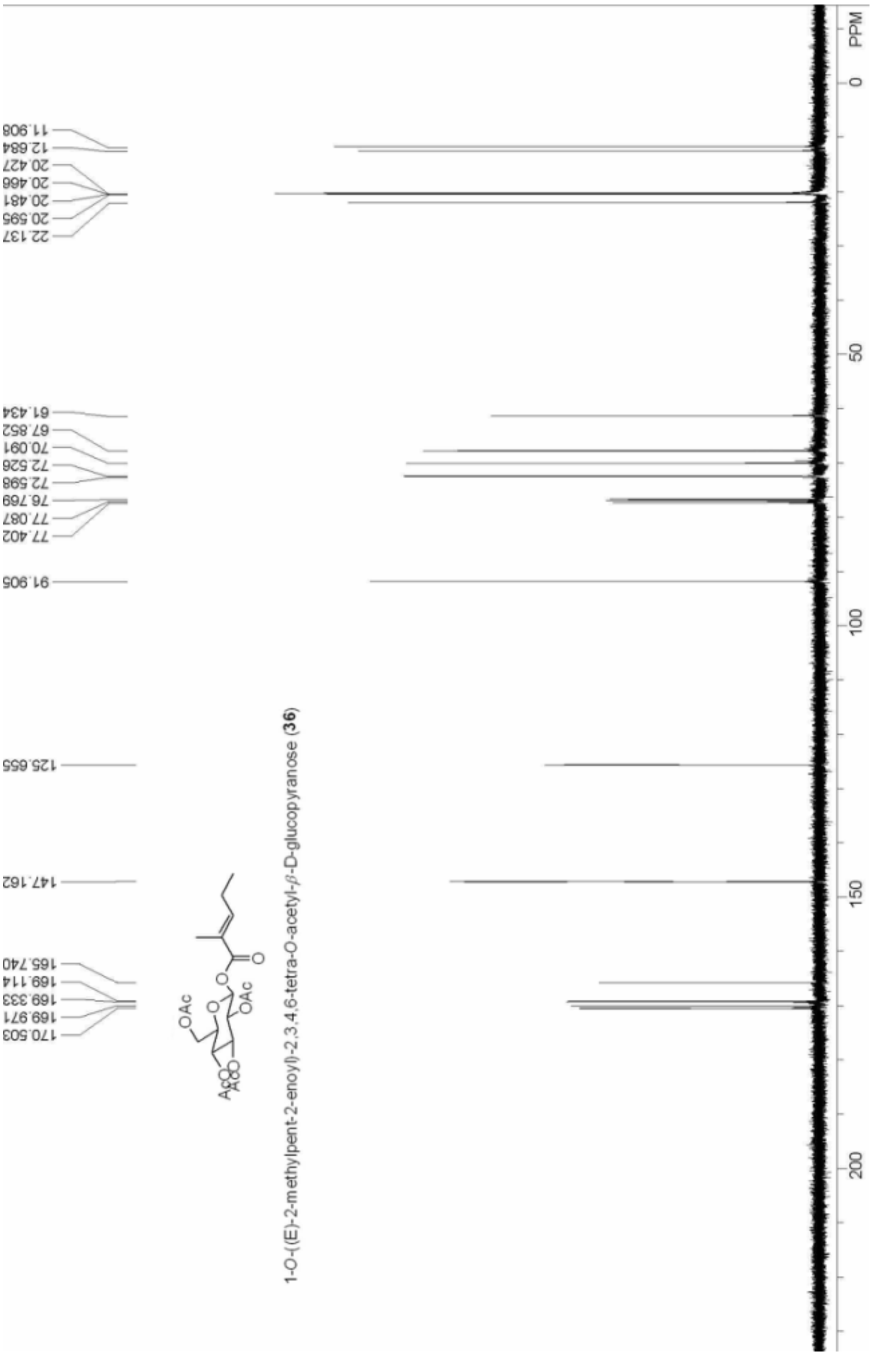

Figure S34. ^{13}C NMR spectrum of compound **36**

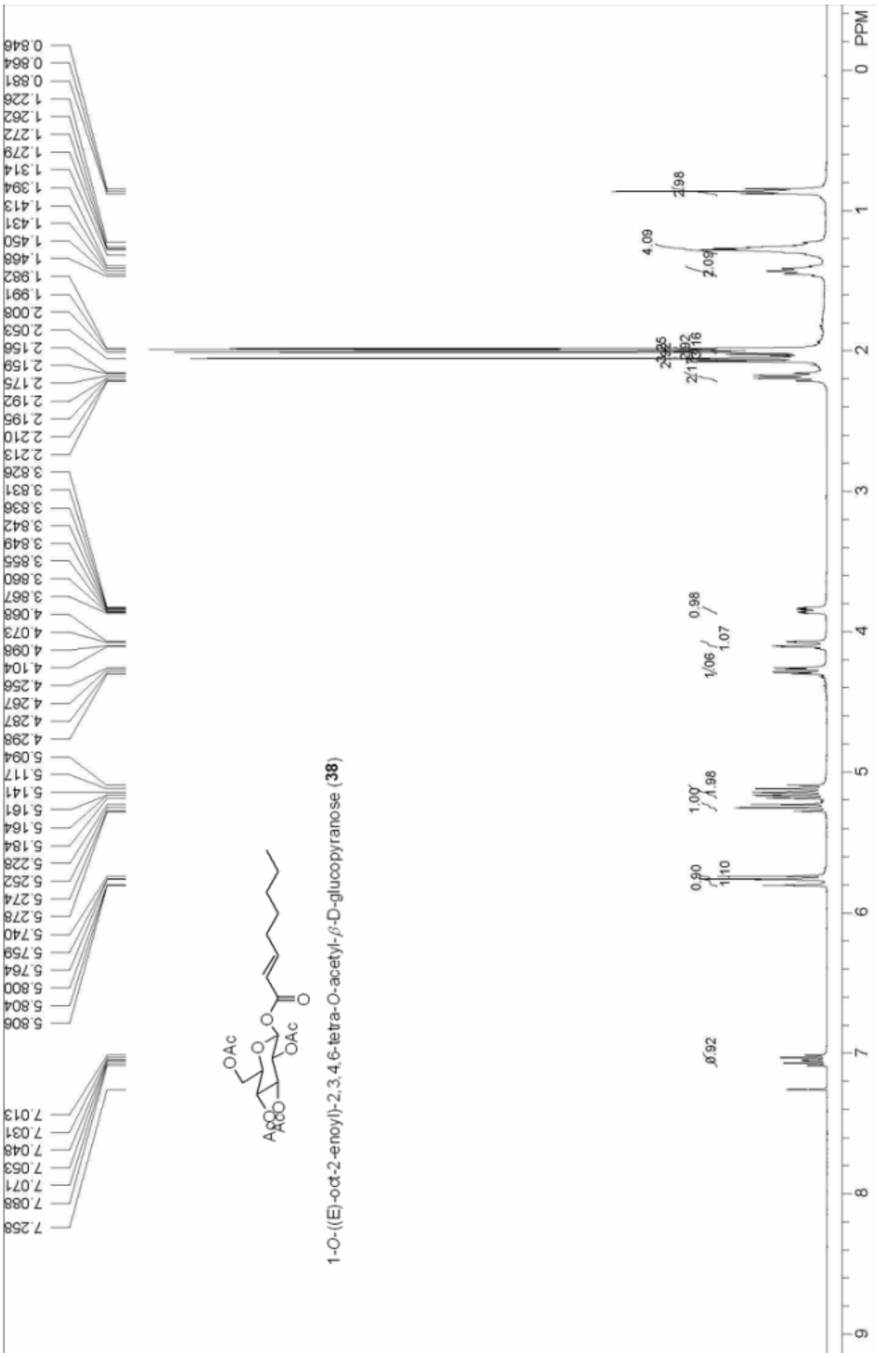

Figure S35. ^{1}H NMR spectrum of compound **38**

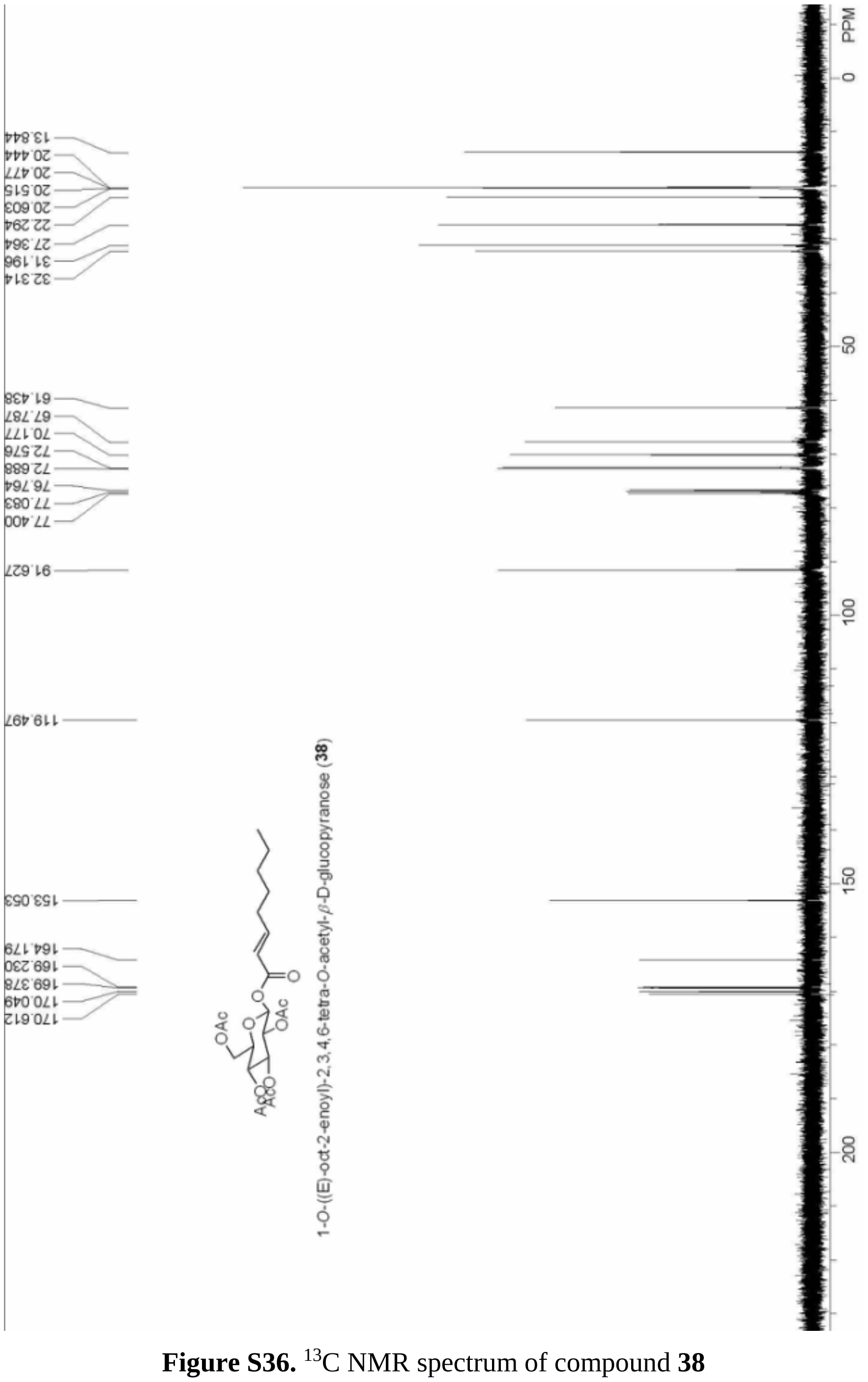

Figure S36. ^{13}C NMR spectrum of compound **38**

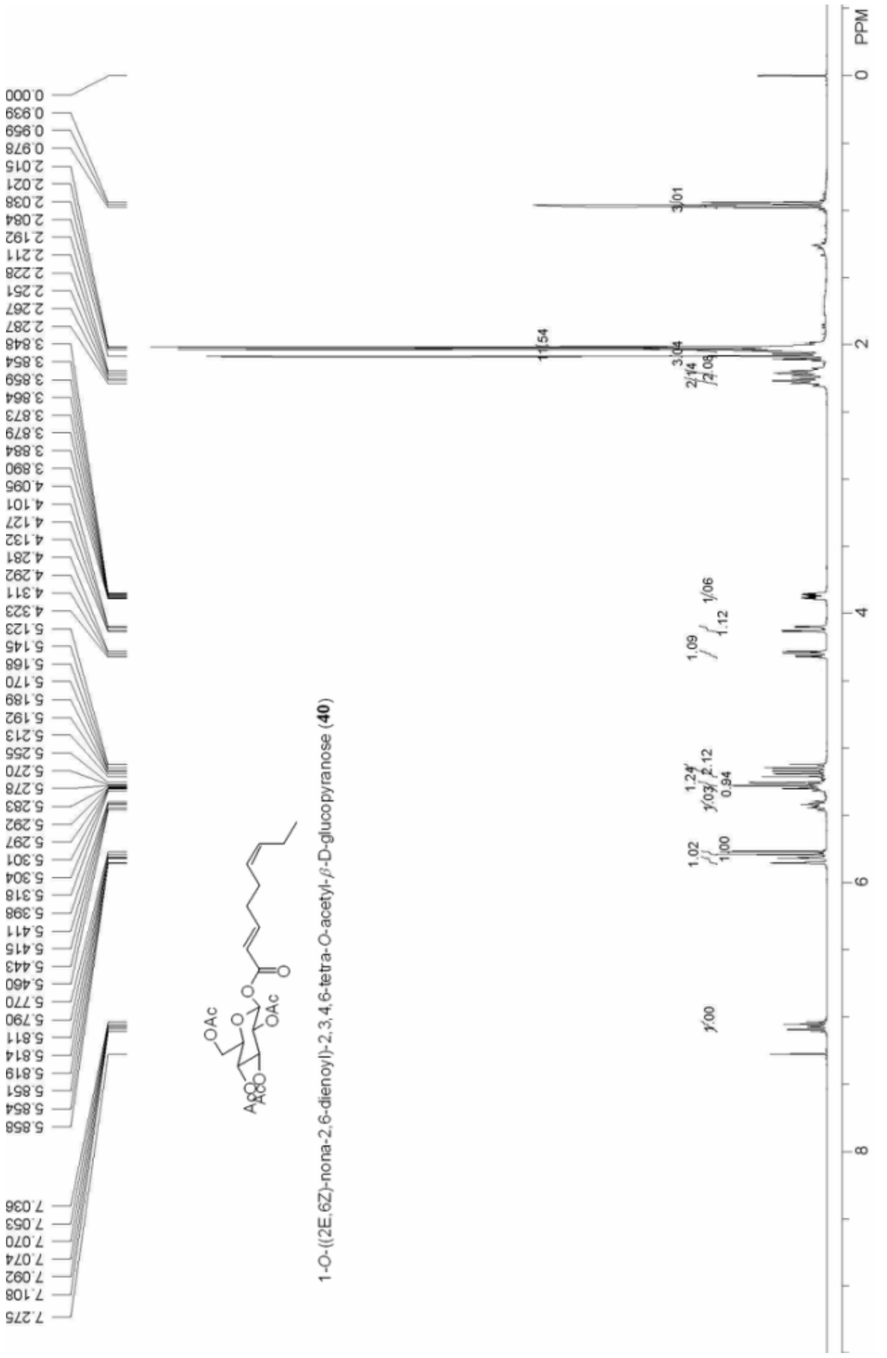

Figure S37. ^{1}H NMR spectrum of compound **40**

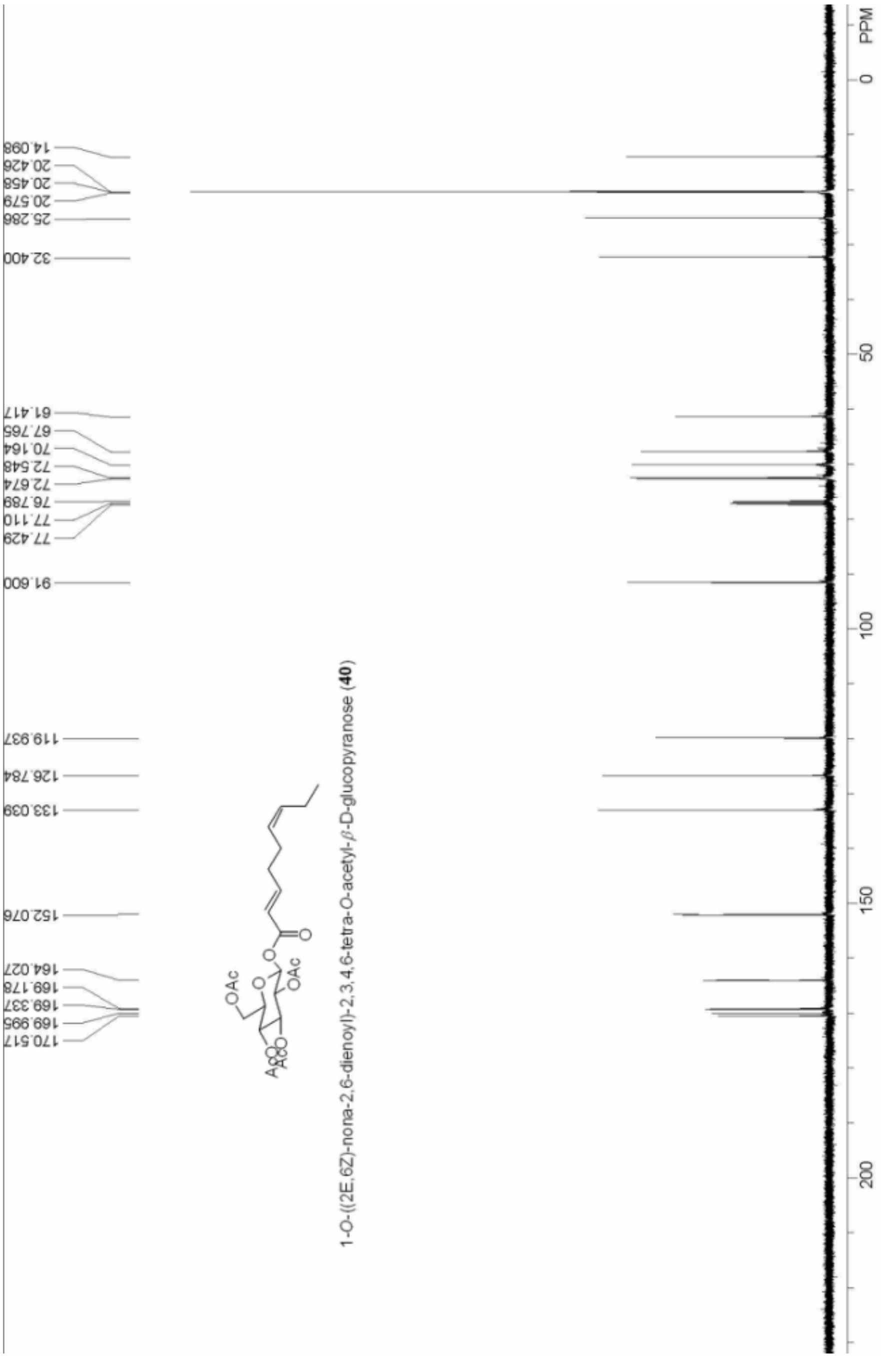

Figure S38. ^{13}C NMR spectrum of compound **40**

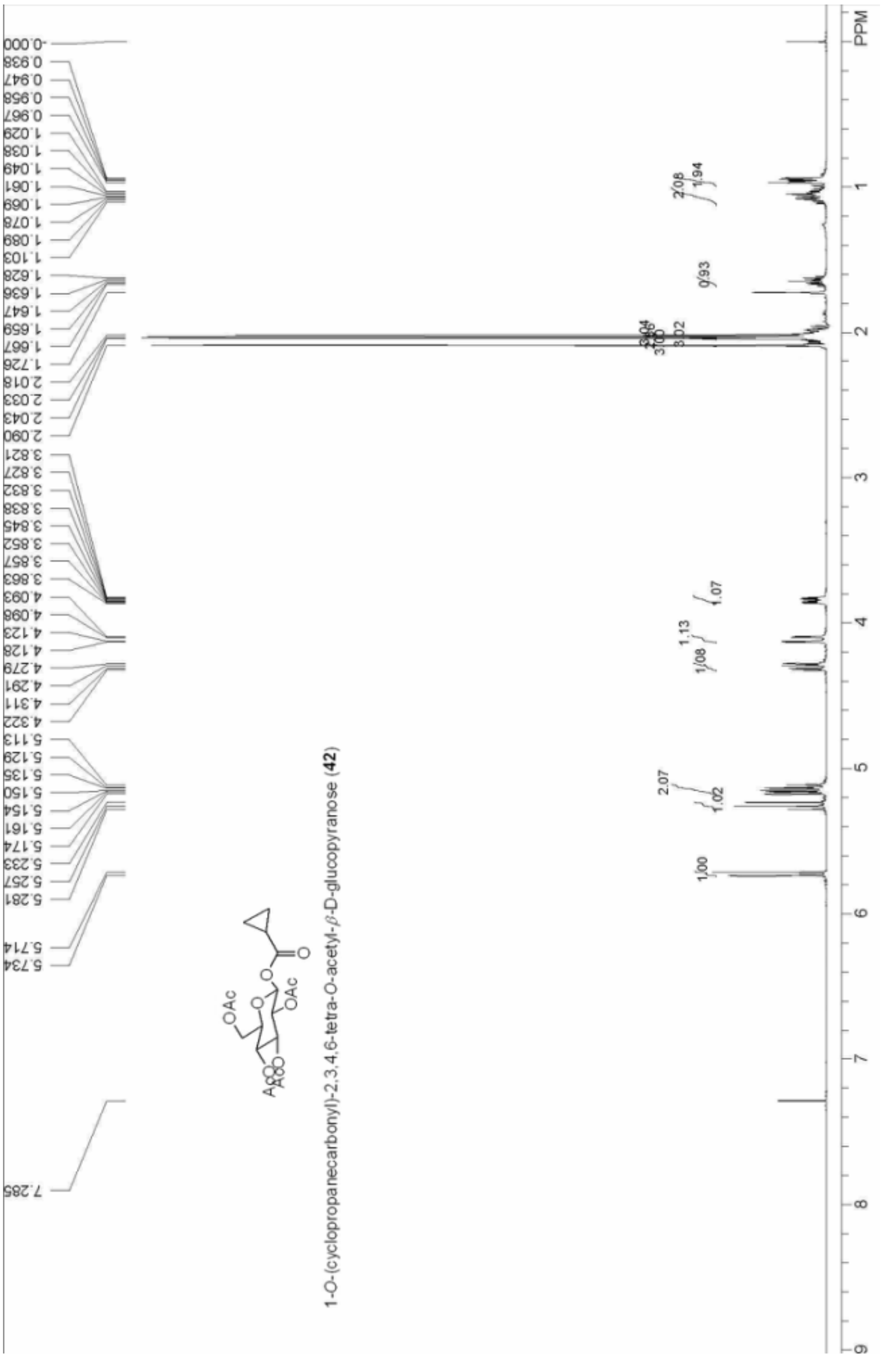

Figure S39. ^{1}H NMR spectrum of compound **42**

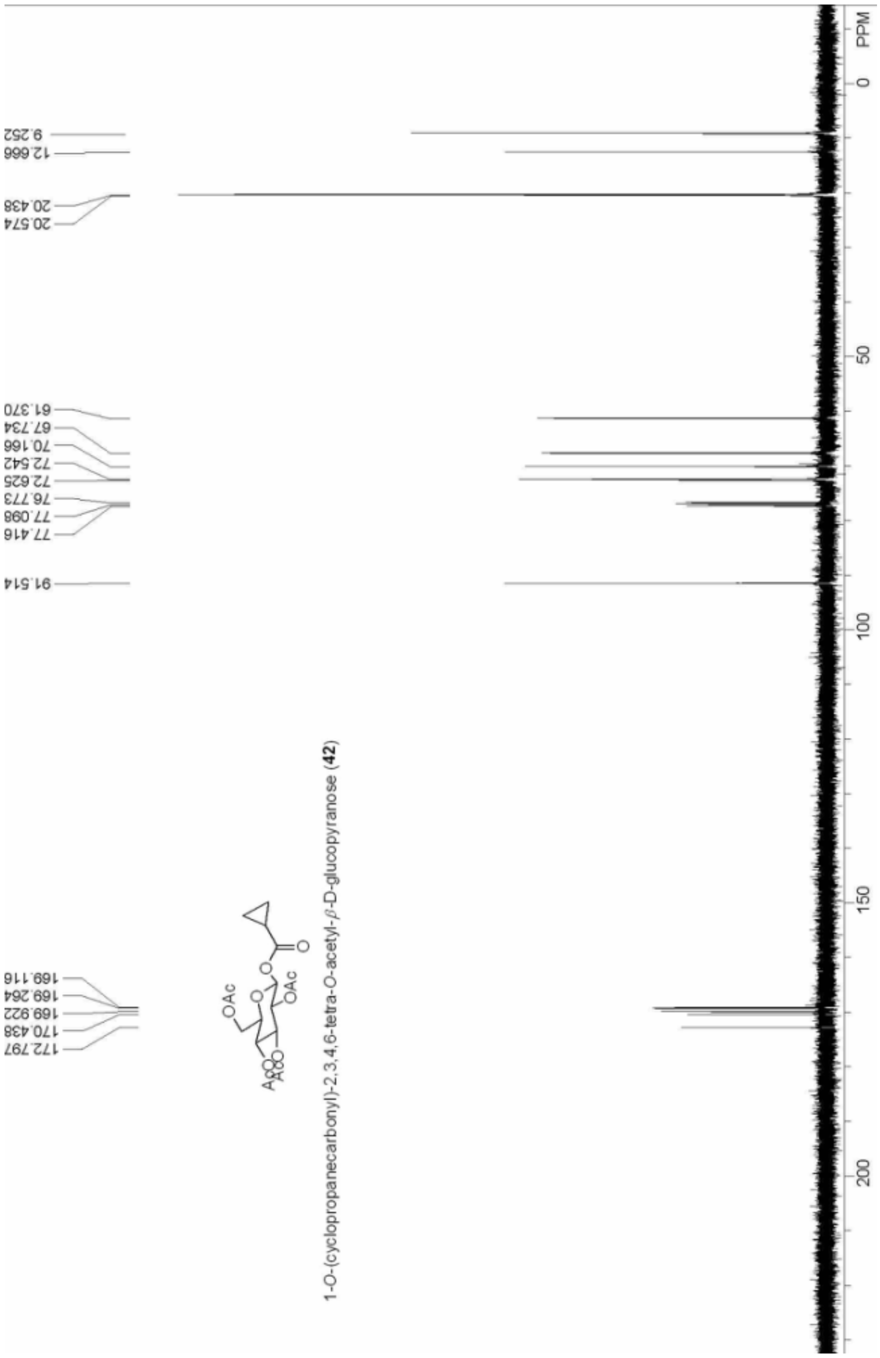

Figure S40. ^{13}C NMR spectrum of compound **42**

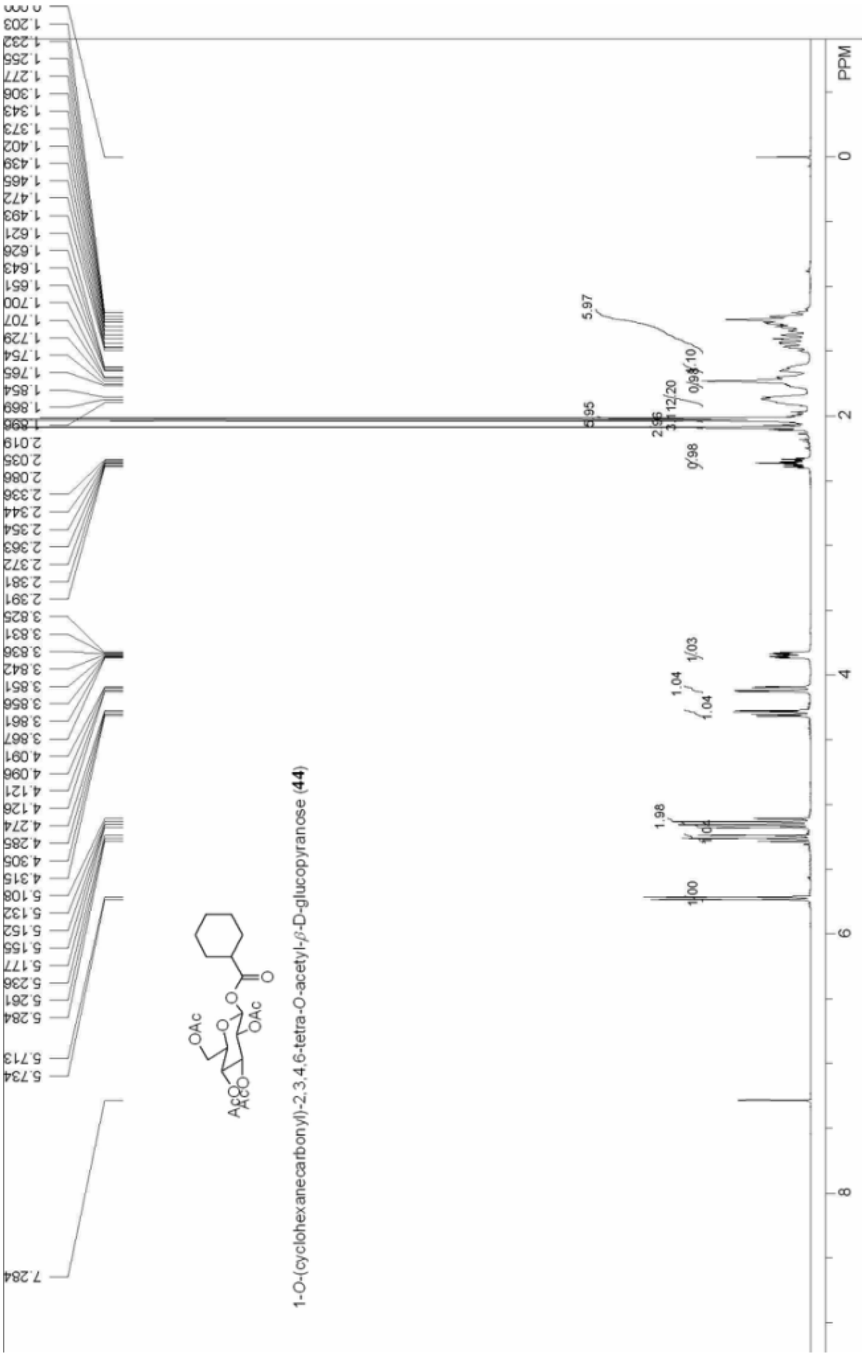

Figure S41. ^{1}H NMR spectrum of compound **44**

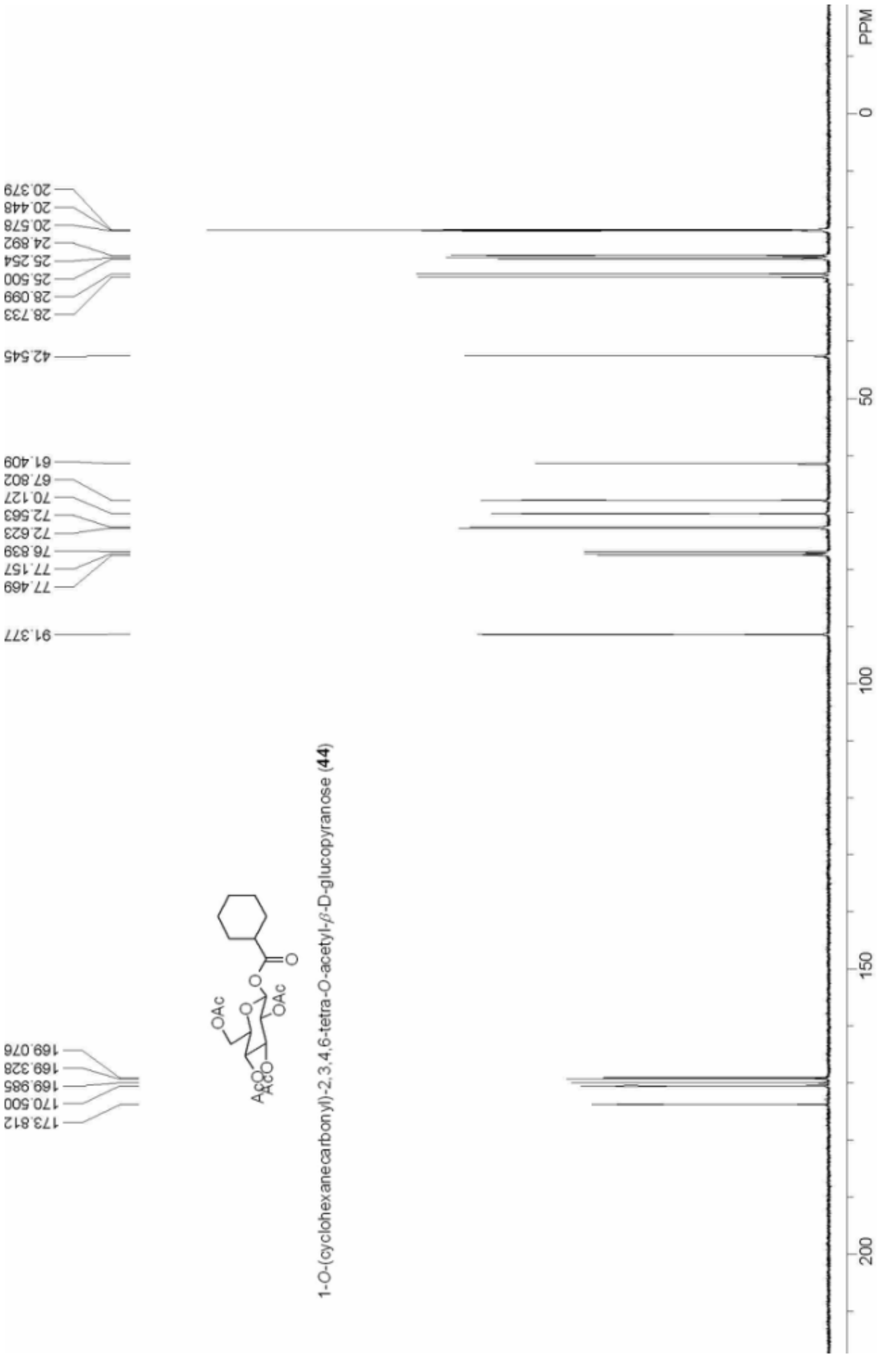

Figure S42. ^{13}C NMR spectrum of compound **44**

Copies of 2D NMR data of compound 8

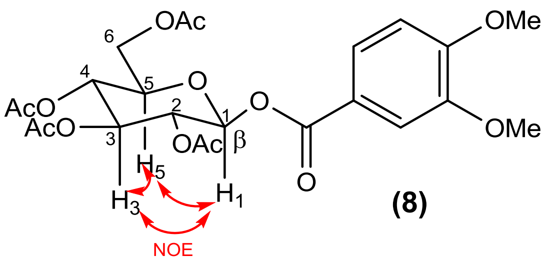

(8)

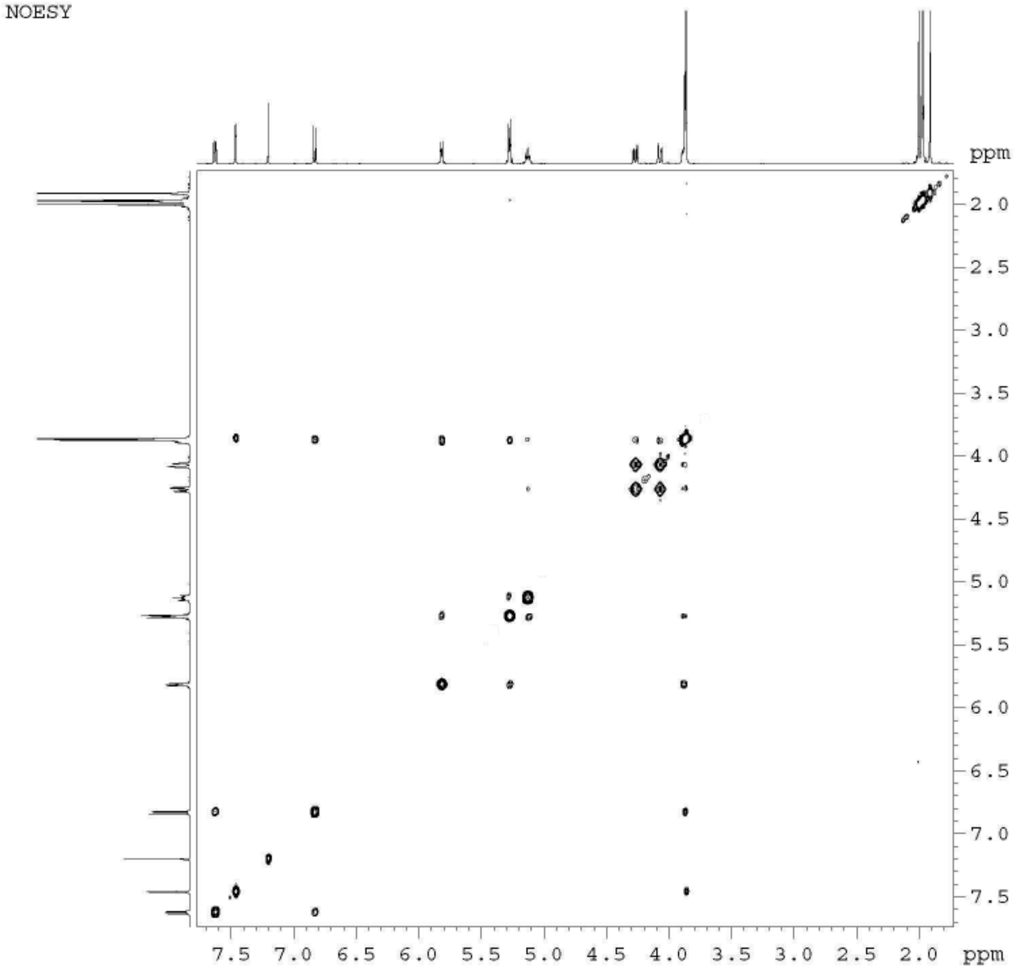

Figure S43. NOESY spectrum of compound **8**

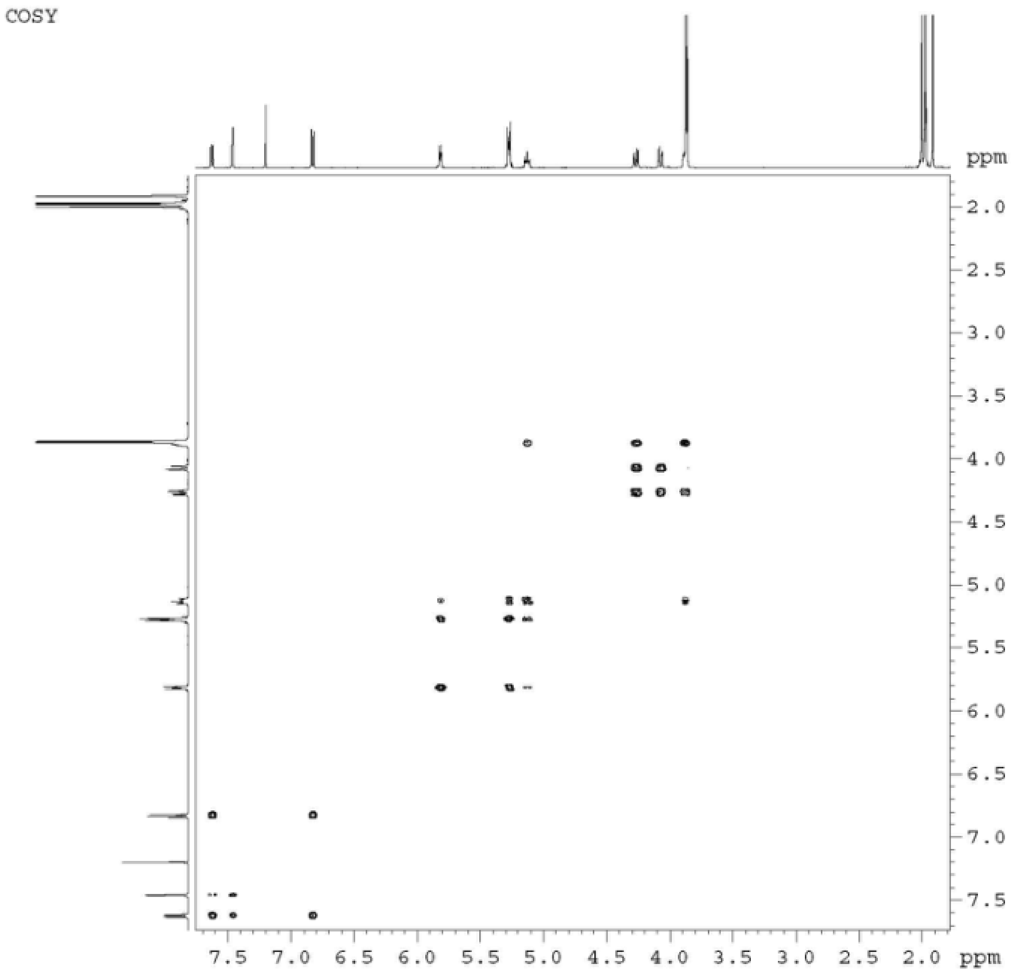

Figure S44. ^{1}H-^{1}H COSY spectrum of compound **8**

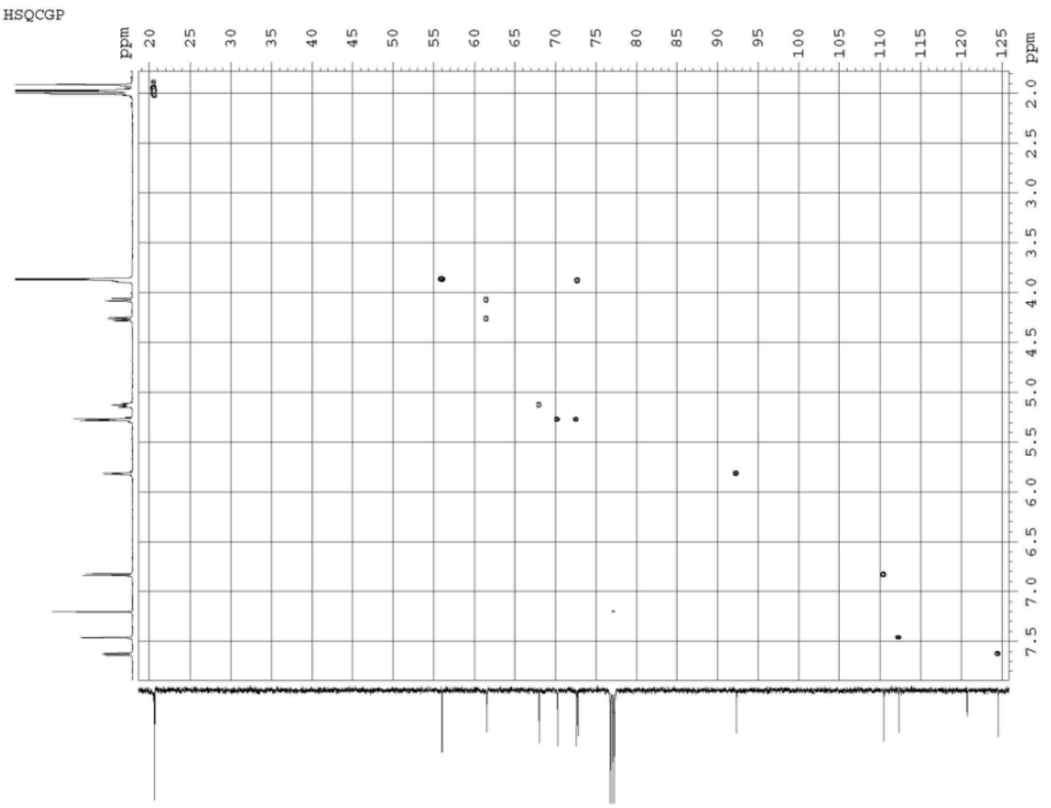

Figure S45. HSQC spectrum of compound **8**
